# Spatial transcriptomics reveals compartment-specific cytotoxic T cell crosstalk with AT2 and goblet cells in advanced COPD

**DOI:** 10.1186/s40364-026-00960-w

**Published:** 2026-06-25

**Authors:** Ayu Hutami Syarif, Bernhard F. E. Reiter, Alexander Avian, Katrin Milger, Elena Ellmeier, Gerald Hoefler, Clemens Aigner, Alberto Benazzo, Sophia Auner, Slaven Crnkovic, Stefano Angiari, Francesca Polverino, Grazyna Kwapiszewska, Leigh M. Marsh

**Affiliations:** 1 Otto Loewi Research Center, Lung Research Cluster, Medical University Graz, 8010 Graz, Austria; 2 Institute for Medical Informatics, Statistics and Documentation, Medical University of Graz, 8010 Graz, Austria; 3 Department of Medicine V, LMU University Hospital, Comprehensive Pneumology Centre (CPC), LMU Munich, Member of the German Centre of Lung Research (DZL), 81377 Munich, Germany; 4 Division of Respiratory Medicine, Department of Internal Medicine, Lung Research Cluster, Medical University of Graz, 8010 Graz, Austria; 5 Division of Pathophysiology and Immunology, Otto Loewi Research Center, Medical University of Graz, 8010 Graz, Austria; 6 Department of Thoracic Surgery, Medical University of Vienna, 1090 Vienna, Austria; 7 Institute for Lung Health, Cardiopulmonary Institute, Member of the German Centre for Lung Research, Justus-Liebig University Giessen, 35392 Giessen, Germany; 8 BioTechMed, Graz, Austria; 9 Department of Medicine, Baylor College of Medicine, TX 77030 Houston, USA

**Keywords:** Spatial Transcriptomics, COPD, Airway, Parenchyma, Immune profiles

## Abstract

**Background:**

Chronic obstructive pulmonary disease (COPD) is characterized by chronic inflammation and structural remodelling across different anatomical structures of the lung, including airways, parenchyma, and pulmonary vessels. However, compartment specific changes in immune cell composition and their communications with underlying structural cells remains incompletely defined.

**Methods:**

Our study sought to assess the spatially resolved cellular organization of healthy and advanced end-stage COPD lungs using 10X Visium spatial transcriptomics. Non-negative matrix factorization was applied to identify transcriptional programs within spatially distinct areas. Data was validated through an integrative multi-modal approach, including flow cytometry, multiplex immunofluorescence, open access GeoMx Nanostring spatial transcriptomics and scRNA-seq datasets, and proof of concept in vitro co-culture experiments.

**Results:**

Spatial mapping of advanced COPD lungs revealed compartment-specific immune and tissue remodelling programs. In the COPD parenchyma, molecular and immune signatures delineated macrophage-rich, remodelling–stress, and humoral B cell immune niches. Airway niches shifted from club cell/innate immunity in controls to goblet cell/adaptive immunity in COPD. Notably cytotoxic T cells colocalised with alveolar type 2 cells within the parenchymal remodelling–stress niche and goblet cells in COPD airways; predicted ligand–receptor interactions implicated these T cells in parenchymal cytotoxic injury and mucus-associated airway remodelling.

**Conclusions:**

Our findings delineate compartment-specific cellular microenvironments and interaction networks that may orchestrate tissue remodelling in COPD. This work advances our understanding of immune-driven pathogenesis and pinpoints the cytotoxic T-cell axis as a candidate for targeted therapeutic strategies.

**Supplementary information:**

The online version contains supplementary material available at https://doi.org/10.1186/s40364-026-00960-w.

## Introduction

Chronic obstructive pulmonary disease (COPD) is the third major cause of death globally and is associated with decreased quality of life [1]. Pathologically, COPD is characterized by chronic inflammation and variable structural changes, including proximal airway obstruction, small airway loss, and parenchymal destruction (emphysema) [2]. Additionally, some COPD patients suffer from concomitant pulmonary hypertension caused by vascular remodelling [3, 4].

Elevated immune cell infiltration characterizes COPD lungs, with several populations correlating with disease severity and progression. Variations in cell abundance often depend on compartment-specific localization [5–8]. For instance in the small airways, increased macrophages, neutrophils, CD8^+^ cytotoxic T cells, and NK cells have been associated with lung function decline [9–11]. On the other hand, cytotoxic T cells have also been observed in the parenchyma and pulmonary arteries, with their presence correlating with the degree of emphysema and arterial intimal thickening, respectively [7, 8].

Advances in single-cell technologies, such as flow cytometry and single-cell RNA sequencing (scRNA-seq), have enabled unbiased immune profiling by identifying and characterizing immune cell subsets in COPD using larger tissue pieces [12–20]. These techniques have given more granular information on immune subpopulations as well as potential cellular interactions. However, both technologies rely on tissue dissociation, which potentially biases isolated cell populations as well as loses information on tissue distribution and the spatial relationship between cell types. This can limit the insights into tissue or compartment specific biological processes and cellular interactions with surrounding tissues.

Recent developments in spatial transcriptomics have provided new opportunities to investigate the relationship between gene expression and tissue localisation by profiling hundreds to thousands of genes in situ. The first spatial profiling of healthy lungs revealed a distinct immune niche within airway submucosal gland areas that recruits IgA-producing plasma cells [21]. In COPD, spatial characterization of lymphoid follicles within emphysema-predominant lungs has identified a signature of B-cell activation that potentially associates with autoimmunity and emphysema progression [22].

In this study, we performed spatially resolved transcriptomics on downsized donor and advanced COPD lungs to investigate tissue architecture, compartmental cellular signalling, and disease-relevant processes. By integrating the spatial transcriptomics with scRNA-seq data and validation with multi panel flow cytometry, multiplex immunofluorescence, GeoMx Nanostring spatial transcriptomics, we identified disease associated spatial niches in the parenchyma, vasculature, and airways.

The spatial colocalisation and predicted cell-cell interactions between cytotoxic T cells and structural cells—Alveolar epithelial type 2 cells (AT2) in the parenchyma, goblet cells in the airway—potentially associate with cytotoxic response in the parenchyma and mucus-associated remodelling in the airway, along with pronounced myofibroblast abundance and extracellular matrix (ECM) associated pathways in perivascular region. Our results provide spatially resolved characterisation of compartment-specific niches that potentially contribute to tissue remodelling process in COPD.

## Materials and methods

### Experimental models and study participant details

For spatial transcriptomics, multiplex immunofluorescence, and flow cytometry, we utilised explanted COPD lungs and healthy control comparison tissue, non-tumorous, donor lung tissue, which was not transplanted due to size reduction from the Department of Thoracic Surgery, Medical University of Vienna, Austria, under protocols approved by the Institutional Review Board (IRB) of Medical University of Vienna (IRB number 976/2010, 1417/2022), and Medical University of Graz (IRB number 1291/2025). Written informed consent was collected from all subjects and clinical data of the patients were available in Tables 1, 2 and 3. Healthy control was obtained from downsized, non-transplanted excess donor lung tissues. Both control and COPD lungs were flushed via ante- and retrograde perfusion with Perfadex (XVIVO perfusion, Sweden) to remove any remaining blood before downstream processing.

**Table 1:** Patient characteristics of the Visium 10X spatial transcriptomics cohort

Patient ID	Age	Sex	Pack years	Quit years	FEV1 (%)	FVC (%)	FEV1/FVC (%)	TLC (%)	DLCOcSB (%)	mPAP (mmHg)	pO2 (mmHg)	Mean Interseptal Distance (µm)	Pathology	CT scan /X-Ray
COPD1	55	F	30	2	16.1	25.9	52.9	164	N/A	38	68.7*	552	Extensive emphysema, peribronchial lymphocytic infiltration, obliterated bronchial lumen, extensive airway remodelling	Severe emphysema predominant upper right lung, mild atelectasis
COPD2	57	M	60	2	19.5	38.1	41	68.5	14.2	45	51.3*	478	Mucous plugs, mild chronic inflammation, goblet cell hyperplasia, prominent inflammatory infiltrates around airways, advanced emphysema, anthracotic pigment deposition in macrophages	Bilateral centrilobular emphysema, post-inflammatory nodules S6R and S3L, dilated bronchi and wall thickening
COPD3	56	F	35	4	18.2	44.4	34.5	174	40.5	30	81*	301	Pan acinar emphysema, fibrosis and smooth muscle hyperplasia	Severe emphysema, “indication of panlobular”
COPD4	59	F	45	5	17	32	44	145	N/A	27	78.1*	418	Mucous plugs, central acinar emphysema, extensive goblet cell hyperplasia, peribronchial chronic inflammatory infiltration	Severe emphysema and central fibrosis (Chest X-ray)
COPD5	56	F	18	4	18.4	42.5	40.5	152	23	30	75*	392	Moderate emphysema, mild lymphocytic infiltration, airway muscle hyperplasia, mild fibrosis	Severe emphysema (Chest X-ray)
Control1	30	M	Never	N/A	N/A	N/A	N/A	N/A	N/A	N/A	N/A	268	Mild ventilator-induced lung injury	N/A
Control2	31	M	Never	N/A	N/A	N/A	N/A	N/A	N/A	N/A	N/A	258	Mild ventilator-induced lung injury	N/A
Control3	68	F	Never	N/A	N/A	N/A	N/A	N/A	N/A	N/A	N/A	459	Age-associated mild emphysema	N/A

Data are presented as mean [range], with n in () indicating the number of individuals from whom data were obtained. Abbreviations: LuTx, lung transplantation; F/M, female/male; pack years, packs of cigarettes smoked per year; quit years, number of years since smoking cessation; N/A, data not available; mPAP, mean pulmonary arterial pressure; mmHg, millimeter of mercury; FEV1, forced expiratory volume in 1 second; %predicted, percent predicted; FVC, forced vital capacity; DLCOcSB, single breath diffusing capacity of the lung for carbon monoxide corrected for haemoglobin; pO2, capillary partial pressure of oxygen; TLC, total lung capacity. Statistical comparison was not performed due to small sample size. *patient was under long-term oxygen therapy

### Spatial transcriptomics

#### Tissue preparation

Lung tissue samples were snap-frozen immediately after collection and stored at −80 °C prior to Spatial Transcriptomics (ST) workflow. Preserved lung tissues were screened for the presence of structural compartments (airways, vessels) using Haematoxylin and Eosin (H&E) staining and to select tissues with good RNA quality (RIN values ≥ 6). In parallel, formalin-fixed paraffin-embedded tissue sections obtained from the same lobe were stained with H&E to evaluate lung morphology and to confirm underlying histopathological features.

#### Permeabilization optimisation for human lung tissues

Frozen tissues were embedded in optimal cutting temperature medium (Tissue-Tek) and were sectioned and placed to fit in capture areas on the Visium Spatial Optimization Slide (Catalogue no. 3,000,394, 10X Genomics) and the Visium Spatial Gene Expression Slide (Catalogue no. 2,000,233, 10X Genomics). Positive control used reference RNA from 2 µL of 1 µg/µL Cervical Adenocarcinoma (HeLa-S3) Total RNA (Catalogue no. AM7852, Thermo Fisher Scientific). Capture areas were scanned using High Content Screening (HCS) Nikon eclipse Ti2 microscope with C2 confocal system (Nikon Instruments, Japan). Optimal permeabilization time (12 minutes) was selected as it resulted in the strongest, most intact fluorescence signal indicating optimal RNA transfer and cDNA synthesis within spots.

#### Sample processing

Cryosections (10 µm-thick) were fixed in methanol at −20 °C and stained with H&E per manufacturer’s protocol. The brightfield images were taken on Olympus VS120 Slide Scanner (Olympus, Japan) at 40X resolution to be used for downstream tissue alignment and gene mapping. Slides were inserted into cassettes to avoid cross leakage among samples. Tissues were permeabilized with Permeabilization Enzyme (Catalogue no. 2,000,214, 10X Genomics) for 12 minutes at 37 °C. RT master mix (10X Genomics) was added into the cassette well and samples underwent reverse transcription. Spatially barcoded cDNA was generated, and the second strand was synthesized using Second Strand Mix (10X Genomics). The cDNA from individual samples were transferred, amplified, and purified using SPRIselect reagent (Beckman Coulter, USA) to isolate optimal cDNA peaks (250–600 base pair). Quantification and quality control of prepared libraries were performed using Bioanalyzer (Agilent). Samples were sequenced on an Illumina NovaSeq S1 Full Flow Cell.

#### Sequence alignment and histological annotation

Tissue alignment was independently performed to manually select tissue areas using Loupe Browser v6.1.0. Sequencing output (Fastq files), alignment json file, and H&E images were processed on Spaceranger v1.3.1 to align the reads with reference genome (GRCh38-2020-A), and to generate count matrix. Count matrix that contains only barcodes associated with tissue areas were used for downstream analysis. Spatial output including tissue positions were loaded in Loupe Browser v9.0.0 to manually annotate spots corresponding to airway, vessel, and areas outside as parenchyma. To improve the quality of manual annotation, compartment-specific signature genes were used. Specifically, annotation was guided by high expression of structural gene markers identified in the integrated scRNA-seq dataset (GSE136831/GSE171541, as described below); for airways, SCGB3A2, KRT17, CAPS, and BPIFB1, and for vascular regions by ACTA2, SFRP2, and ENPP2. Areas negative for these markers and negative for macroscopically visible structures (airways, vessels) were designated as parenchyma.

#### Data analysis

A graphical representation of the data analysis workflow is available in Supplementary Figure S1. To account for tissue heterogeneity, each individual sample was treated as an individual batch. The Spaceranger output (gene matrix and tissue image) was loaded as a Seurat object in the R environment and all analysis steps performed in R (v4.4.1) [23].

#### Quality control and filtering

Data were subjected to quality control by assessing number of unique genes detected, number of UMI counts, and percentage of mitochondrial RNA. Spots with low library size (<100–150 nCount), expressed fewer than 100–150 genes, and contained more than 20% mitochondrial transcripts were excluded. To generate biologically relevant information, we subset the feature genes to retain only the protein coding genes identified from EnsDb.Hsapiens.v86 package [24].

#### Data transformation and normalization

Each sample was transformed, normalized, and scaled individually using *SCTransform()* from Seurat package. All samples were merged and processed using SCT assay. Layers of merged objects were joined, normalized, and scaled across spot level gene matrix (Spatial assay).

#### Pseudo-bulk differential expression analysis

For differential expression analysis, we aggregated the spatial gene expression of pooled spots from all samples by grouping disease or manually annotated compartments niches and individual samples using *AggregateExpression*() function from Seurat. Calculation using the aggregated gene expression was performed to adjust for unbalanced number of spots across compartments and disease. Count matrices and metadata containing individual identity numbers, spatial niches, and disease were taken and loaded into *DESeqDataSetFromMatrix*() function from DESeq2 package [25]. Size factors and dispersions were estimated, and negative binomial Wald test was applied to model the gene expression data. Comparison between COPD and control or pairwise comparison among spatial niches were calculated on aggregated individual expression data and multiple comparison was adjusted with Benjamini-Hochberg method. Only significant differentially expressed genes (adjusted *p*-value ≤ 0.05) were highlighted in colours in volcano plot created by ggplot2 package. Significant genes were further used to calculate pathway enrichment scores.

#### Spot deconvolution

Deconvolution was performed on raw count of individual samples with *create.RCTD()* function using the following parameters: UMI_min: 50, counts_min: 5, max_cores: 8, test_mode: 8. We ran the RCTD using full doublet mode to allow identification of multiple cell types within a spot. Cell type proportion was then summed to one for each spot to provide relative composition of a given cell type within spots. To avoid the contribution of background signal as false positive and account for sample heterogeneity, we retained only top 50% of the values for each cell type in each individual samples. Filtered cell type proportion for each spot was integrated into the metadata and was referred as cell-type-associated transcript abundance. Deconvolution quality was verified by 1) comparing scRNA-seq derived gene marker expression for all deconvoluted subpopulations with deconvolution results, and 2) comparing scRNA-seq derived gene marker expression and deconvolution results across manually annotated compartments. Cell-type-associated transcript abundance was compared across disease and niches. Abundance values above zero was log_10_(x + 0.00001)-transformed and was used as input for linear mixed model applied in lmerTest and emmeans package [26] to account for multiple data per individual sample, with the following formulas: 1) cell abundance ~ niches+(1|individual sample) and 2) cell abundance ~ niches*diagnosis+(1|individual sample). Niches and diagnosis were fixed effects whereas individual sample was assigned as random factor. To assess pairwise contrast of each variable component, post-hoc comparisons were performed using False Discovery Rate (FDR) correction. Estimated means were calculated using emmeans package and plotted with standard error.

#### Non-negative matrix factorization

Layers of merged Seurat object were joined, normalised, and scaled using Spatial assay containing spot level gene matrix. Seurat object was converted to version 3 and Spatial assay was renamed to “RNA”. To allow for reproducibility, seed was set at 123. Non-negative matrix factorization (NMF) was computed using *RunNMF*() function from singlet package [27] with the following parameters: assay:”RNA”, slot:”data”, reps: 5, k: c(5,10,15,20,25,30,35,40). We selected 10–20 as the optimal k factor with relatively lower test set error and accurate representation of morphological lung compartments.

To better understand the spatial heterogeneity across niches, we isolated their most active spots denoted by top 5% spots expressing any given niches. We pooled active spots from all niches and performed maximum-values categorization to assign exclusively each spot to a niche (Fig. S11A-B). For parenchyma associated niches, control-NMF was constructed by pooling spatial transcriptomic spots assigned to NMF-derived niches (NMF_2, NMF_6, and NMF_11), which were exclusively identified in control samples, exhibited a high degree of similarity and consistent enrichment of the control associated genes and pathways.

#### Gene gradient analysis

Tissue coordinates, tissue image, and scale factors as outputs from spaceranger were loaded to create Staffli object using STutility (v1.1.1) [28] and semla package (v1.1.6) [29]. Staffli object was embedded into the merged Seurat object. Radial distance was calculated using “airway” or “parenchyma” annotation as region of interest. Spatially disconnected spots were excluded, and pixel distance was converted to microns and to a square root of the distance for better visualization. Spatial heatmap of radial distances were plotted using *MapFeatures*() function.

#### Pathway enrichment analysis

Gene ontology (GO) enrichment was performed using *enrichGO*() function from clusterProfiler package [30] using all detected genes as background, org.Hs.eg.db as database [31], “BP” or “CC” ontology, *p*-value adjustment of Benjamini-Hochberg, and qvalue of 0.05. Parent ontologies from all significant pathways (p_adj_ ≤ 0.05) were calculated using rrvgo package by calculating score similarity matrix using relevance “Rel” method. GO terms were summarized based on their semantic similarity and scores using default similarity threshold of 0.7. Top parent pathways from each niche, were selected based on their representative scores.

#### Pathway correlation with cell deconvolution

Module scores within spots were calculated from selected top parent GO biological process and cellular component pathways using *AddModuleScore*() function from Seurat package. Pearson correlation between deconvolution of certain cell types and specific module scores were calculated. Top correlations were visualised using ggplot2 package. 

### H&E staining and quantification

Lung tissues from the same lobe used in the Visium experiment were fixed in formalin, embedded in paraffin, and sectioned at 2.5 µm thickness. The sections were subjected to H&E staining and scanned with VS120 Slide Scanner (Olympus, Japan). Unbiased design-based stereology was applied using new Computer Assisted Stereological Toolbox (newCAST, Visiopharm, Denmark), as described elsewhere [4, 17]. Mean interseptal distance was used as a statistical estimate of emphysema and was quantified in at least 50 systematically selected random areas per lung. 

### Multiplex immunofluorescence staining

Formalin-fixed paraffin-embedded tissue sections with a thickness of 2.5 µm were subjected to an adapted OPAL (Akoya Biosciences, USA) dye-based multi-immunolabelling protocol. For each panel 3 Donor and 3 COPD patients were used (Table 2).

**Table 2:** Patient characteristics of the immunofluorescence cohort

Parameters	Overview Panel		Parenchyma Panel		Airway Panel	
	Control (n = 3)	COPD (n = 3)	Control (n = 3)	COPD (n = 3)	Control (n = 3)	COPD (n = 3)
Age at LuTx	43/71/53	68/64.9/65.7	43/58/55	59.4/59.4/54.3	43/71/55	63.6/56.5/59.4
Sex (F/M)	F/F/F	M/F/M	F/F/F	F/M/F	F/F/F	M/F/F
Pack years	35/18/4	45/60/30	-/Never/Ex	30/37.5/20	35/18/Ex	70/-/45
Quit years	-/-/-	-/-/-	-/-/5	5/-/7	-/-/5	3/13/5
mPAP, mmHg	N/A	21/22/36	N/A	27/45/40	N/A	30/36/27
FEV1, %predicted	N/A	26.8/25/16	N/A	17/51.7/18	N/A	16/19/17
FVC, %predicted	N/A	53.9/63/47	N/A	32/83/29	N/A	51/53/32
FEV1/FVC (%)	N/A	49/34/27	N/A	44/-/-	N/A	24/31/44
DLCOcSB, %predicted	N/A	33.4/-/29	N/A	-/9.66/-	N/A	35.2/-/-
TLC, %predicted	N/A	116.4/139/63	N/A	145/112.7/119	N/A	102/230.2/145
pO_2,_ mmHg	N/A	88.2/72/80.8	N/A	78.1/36/61.8	N/A	64.7/51.5/78.1

Data are presented as mean [range], with n in () indicating the number of individuals from whom data were obtained. Abbreviations: LuTx, lung transplantation; F/M, female/male; pack years, packs of cigarettes smoked per year; quit years, number of years since smoking cessation; N/A, data not available; mPAP, mean pulmonary arterial pressure; mmHg, millimetre of mercury; FEV1, forced expiratory volume in 1 second; Never, never smoker; Ex, Ex-smoker; %predicted, percent predicted; FVC, forced vital capacity; DLCOcSB, single breath diffusing capacity of the lung for carbon monoxide corrected for haemoglobin; pO2, capillary partial pressure of oxygen; TLC, total lung capacity. Statistical comparison was not performed due to small sample size. *patient was under long-term oxygen therapy

Slides were deparaffinized by melting the paraffin at 60 °C for one hour, followed by a 10-minute incubation in Roticlear (Carl Roth, Germany). Subsequent rehydration was performed stepwise using a series of decreasing ethanol concentrations (96% to 50%). The staining protocol consists of a repeating cycle starting with a heat-induced antigen retrieval step at pH6 or pH9 (DAKO by Agilent Technologies, Denmark) using a pressure cooker at 80 kPa for 15 minutes, followed by 20-minute cooling on ice. Tissues were then blocked for 10 minutes with Antibody Diluent/Block (ARD1001EA, Perkin Elmer, USA) and primary antibody incubation overnight at 4 °C in a humidity chamber. The following day, slides were washed with TBST and incubated with secondary ImmPRESS® HRP anti- mouse or -rabbit (MP-7402 and MP-7401, Vector Laboratories, USA), respectively, for 10 minutes at room temperature. Following a rinse and wash cycle with TBST, the slides were incubated with a 1:300 dilution of the OPAL substrate for 20 minutes at room temperature. Finally, slides were treated with DAPI or Hoechst 33342 (1:1000, Thermo Fisher Scientific, USA) for 10 minutes at room temperature. A comprehensive overview of the antibody specification, combination, staining sequence, and pH specification can be found in Supplementary Table S1.

Slides were mounted with fluorescence mounting medium (S3023, DAKO by Agilent Technologies, Denmark). Image acquisition was performed using Olympus VS200 Slide Scanner with an HC PL APO CS2 20x/ 1.25 NA glycerine immersion objective (Olympus, Germany). All OPAL dyes were acquired using hybrid detectors in the following configuration: 488 nm excitation with 520/20 nm emission for OPAL 520; 552 nm excitation with 568/20 nm emission for OPAL 570, and 614/20 nm emission for OPAL 620; 638 nm excitation with 695/20 nm emission for OPAL 690. Final postprocessing of the image such as background reduction was performed in Qupath v0.5.1 and ImageJ 1.54 g with additional plugin from FigureJ v1.36.

### Multi panel flow cytometry

Lung pieces from downsized donors (*n* = 5) and explanted COPD lungs (*n* = 19) were used to identify structural compartments (airway and pulmonary arteries). Cohort demographics are provided in Table 3. Third- to fourth generation of airways and vessels from each lung were freshly isolated, weighed, and processed to create single-cell suspensions. Separated lung tissues from adjacent tissue area were further dissected to remove any macroscopically visible airways or vessels, as described previously [17, 32]. Cells in the suspensions were counted and stained with a viability dye and multi-panel antibodies using the protocol as previously described [17, 32]. A list of antibodies is attached in supplementary Table S2 (panel 1–4). Measurements were performed using Cytoflex SII Flow Cytometer (Beckman Coulter, Austria). The results were compensated using CytExpert Acquisition and Analysis Software version 2.5 (Beckman Coulter, Austria) and were subjected to quality control with FlowAI v1.28.0.

**Table 3:** Patient characteristics of the flow cytometry cohort

Parameters	Control (n = 5)	COPD (n = 19)
Age at LuTx	46.6 [21–57] (5)	62.8 [53.3–70.5] (19)
Sex (F/M)	2/3	8/11
Pack years	19 [4–35] (3)	53.3 [10–160] (19)
Quit years	N/A	N/A
mPAP, mmHg	N/A	26.8 [21–34] (13)
FEV1, %predicted	N/A	22.5 [10.2–82] (19)
FVC, %predicted	N/A	51.5 [21–117] (17)
FEV1/FVC	N/A	0.4 [0.20–0.70] (17)
DLCOcSB, %predicted	N/A	31.4 [23.1–51.5] (10)
TLC, %predicted	N/A	135.5 [63–262] (17)
pO_2,_ mmHg	281.05 [45.2–388] (4)	67.9 [51.9–88.2] (19)

Data are presented as mean [range], with n in () indicating the number of individuals from whom data were obtained. Abbreviations: LuTx, lung transplantation; F/M, female/male; pack years, packs of cigarettes smoked per year; quit years, number of years since smoking cessation; N/A, data not available; mPAP, mean pulmonary arterial pressure; mmHg, millimetre of mercury; FEV1, forced expiratory volume in 1 second; %predicted, percent predicted; FVC, forced vital capacity; DLCOcSB, single breath diffusing capacity of the lung for carbon monoxide corrected for haemoglobin; pO2, capillary partial pressure of oxygen; TLC, total lung capacity. Statistical comparison was not performed due to small sample size

The datasets were analysed using FlowJo v10 (FlowJo, LLC, USA) and the populations were gated using the strategy available in Supplementary Figure 7A. A total of 11 populations were identified and quantified from 4 panels (Table S3). The parent population i.e., CD3^+^ cells were excluded to avoid redundancy. The results from two lung parenchyma regions from the same patient were averaged. All populations were presented as a percentage of CD45^+^ (%CD45). The cell proportion matrix was then used for the downstream analysis.

Data was LOG transformed using log_10_(%CD45+0.00001) to improve the normality of the data. Cell proportion was compared across disease and compartments. Proportion values were used as input for linear mixed model to account for multiple sampling per individual lung, with the following formula: cell percentage ~ compartment*disease+(1|individual sample) [33]. Compartment and disease were fixed effects whereas individual lungs were assigned as random factor. To assess pairwise contrast of each variable component, post-hoc comparisons were performed using FDR method. Estimated means were calculated using emmeans package and plotted with standard error.

### Co-culture experiments

#### CD8^+^ cytotoxic T cell culture

Peripheral blood mononuclear cells were isolated as previously reported [34] from buffy coats of healthy donors (*n* = 3), naïve CD8^+^ T cells purified using the EasySep™ Human Naïve CD8^+^ T Cell Isolation Kit II (STEMCELL Technologies) and stored in liquid nitrogen until use.

For all experiments, T cells were thawed, washed, counted, and activated in 96-well U-bottom using plate-bound anti-human CD3 and anti-human CD28 antibodies (1.0 µg/ml and 2.0 µg/ml, respectively; BioLegend) in X-VIVOTM 15 medium (Lonza) for 4 days. For intracellular cytokine analysis, activated CD8^+^ cells were restimulated with Cell Activation Cocktail (0.5X; BioLegend) containing phorbol 12-myristate 13-acetate (PMA) and ionomycin (Iono) for 4.5 hours with addition of Brefeldin A (BFA;10 µg/ml; BioLegend) after 2 hours. Cells were stained with Fixable Viability Dye (FVD) eFluor™ 780 (Thermo Fisher Scientific), fixed, permeabilized (BioLegend), and stained against IFN-γ (Table S2 panel 5; BioLegend). Cells were acquired on a CytoFLEX LX Flow Cytometer (Beckman Coulter). Unstimulated and BFA controls were used to set the gates and analysis was performed using FlowJo (BD Biosciences).

#### Epithelial – T cell co-culture

Human epithelial A549 cells (ATCC) were cultured in complete medium (DMEM/F-12 with 10% FBS, 1% glutamine, 0,1% PenStrep, Dulbecco; Thermo Fisher Scientific, USA). For all experiments, A549 cells were seeded in 24 well plate one day before co-culture (200,000 cells/well). After 24 hours, pre-washed, pre-activated CD8^+^ cytotoxic T cells in complete media and IL-2 (50 U/mL, PeproTech) were added into the well (200,000 cells/well) for 30 minutes. Additional controls with A549 cells only or inhibition with additional IFN-γ antibody (10 µg/mL; BioLegend) or IgG1 Isotype control (10 µg/mL; Abcam), pre-incubated with CD8^+^ cytotoxic T cells before co-culture. Cells were fixed with intracellular (IC) fixation buffer (Thermo Fisher) followed by detachment using TrypLE Select Enzyme (Gibco) at 37 °C for 10 minutes, followed by permeabilization with 90% methanol (Merck), stained with antibodies (Table S2 panel 6), and measured in Cytoflex SII Flow Cytometer. The results were compensated using CytExpert Acquisition and Analysis Software

#### Immunoblotting blotting

A549 cells were stimulated with different concentration of IFN-γ (0,1,10,100 ng/mL) for 15 minutes. Cells were detached, washed, and collected in RIPA and sonicated for cell lysis. SDS-PAGE and wet immunoblotting was performed using the Bio-Rad Mini Trans blot system. Nitrocellulose membranes were blocked with 5% BSA in TBST, and stained with antibodies (Table S2 panel 7). Western Blot was visualised and quantified with Image Lab 6.1 (Bio-Rad).

### Open access scRNA-seq dataset

#### Cohort and data analysis

A graphical representation of the data analysis workflow is available in Supplementary Figure S2. GSE171541 [19] dataset consisted of control (*n* = 6) and COPD (*n* = 3) lungs from untreated, primary, non-metastatic lung tumour patients undergoing curative surgery as previously described. GSE136831 [20] dataset was subsetted to include controls (*n* = 15) and advanced COPD (*n* = 17) lungs undergoing lung transplantation. GSE171541 and GSE136831 were concatenated to form the reference scRNA-seq dataset. GSE173896 [18] dataset comprised of GOLD1-2 COPD lungs (*n* = 9) and controls (*n* = 7) of patients undergoing lobe resection for localized lung cancer. Clinical information about the datasets is available in the original publications [18–20]. To account for sample heterogeneity, each individual sample within each dataset was treated as an individual batch. The count matrix, cell barcodes, and gene names were loaded as a Seurat object in R environment.

#### Quality control and filtering

For GSE136831, multiplet annotation and rare cell type i.e., Mesothelial were removed. Cells underwent quality control by assessing number of unique genes expressed (nFeature_RNA), library size (nCount_RNA), and percentage of mitochondrial gene content. For GSE136831 and GSE171541, cells were filtered using the following thresholds: 500 ≤ nCount_RNA ≥40000 and 250 ≤ nFeature_RNA ≥7000. Samples with low number of cells (≤100) were removed. For GSE173896, only cells with percentage of mitochondrial genes ≤ 20 and 500 ≤ nFeature_RNA ≥8000 were retained for downstream analysis.

#### Data transformation, normalization, and dimensionality reduction

Independently, each dataset was split by samples and subjected to transformation using *SCTransform*() function applied in Seurat package. Individual samples within each dataset were merged and underwent PCA using features identified with *SelectIntegrationFeatures*() function in Seurat. Individual samples as batches were integrated using Harmony package with PCA embedding. Uniform Manifold Approximation and Projection (UMAP) was performed using harmony [35] embedding with 30 dimensions for GSE136831 and 15 dimensions for GSE171541 and GSE173896.

#### Dataset integration

Layers of GSE171541 and GSE136831 dataset was joined using RNA assay and transformed using SCTransform [36]. Integration features were determined using *SelectIntegrationFeatures*() function with predefined number of features of 3000. Datasets were integrated using anchor set defined by *FindIntegrationAnchors*() function using SCT assay, integration features, and GSE171541 as a reference dataset. Dimensionality reduction (PCA and UMAP) was performed on the integrated dataset.

To unify the cell annotation, we map the cell annotation from GSE136831 as it contains comprehensive structural and immune sub types. We used transfer anchors using GSE136831 as the reference and GSE171541 as the query dataset with 30 dimensions.

All annotation labels from GSE171541 and GSE173896 were unified, where possible, following cell annotations from GSE136831 except neutrophils, retained from original GSE171541 and GSE173896 dataset and was confirmed with specific neutrophil gene markers.

#### Deconvolution reference

Integrated scRNA-seq dataset (GSE136831, GSE171541) was used as a reference for cellular deconvolution. Cell types with presence in more than 50% samples were retained, except neutrophils which were detected only from GSE171541. To generate biologically relevant information and match genes in spatial data, we subset the genes to retain only the protein coding genes identified from EnsDb.Hsapiens.v86 package. We created scRNA-seq reference using RCTD package [37] with *Reference*() function from count matrix, cell type identity, and UMI counts.

#### scRNA-seq ligand receptor analysis

Weighted networks and ligand-target matrices used the default database from nichenetr package. Integrated scRNA-seq dataset was subsetted to include only cell types of interest. Potential ligands and receptors were defined as genes expressed in minimum 5% of the cell type clusters. Target gene sets were defined as differentially expressed genes between COPD and control in receiver cell types that reached statistical significance (p_adj_ ≤ 0.05 and log2fold change ≥ 1.5). Ligand activities were predicted using target gene sets and ligand-target matrix. Potential ligands, and background expressed genes, calculated from detected ligand genes in receiver cell types. Ligand-receptor pairs were prioritized if: 1) the ligands and receptors were upregulated in sender or receiver cell type relative to other cell types, 2) ligands or receptors were differentially expressed between COPD and control, 3) the ligands and receptors showed higher average expression in sender or receiver cell types, and 4) the ligands have corrected area under the precision-recall value > 0. In case of multiple senders or receivers, we applied additional prioritization to infer specific interactions based on average ligand or receptor expression in each cell type. Furthermore, we retained only significantly expressed ligands (p_adj_ ≤ 0.05) between COPD and control in the corresponding cell type.

#### Spatially resolved ligand receptor analysis

Ligand-receptor gene pairs identified in scRNA-seq analysis were subjected to spatial neighbourhood analysis independently for each sample. Specifically, for each ligand-receptor pair, the total number of receptor-expressing spots located within 200 µm of ligand-expressing spot was defined as the number of spatially feasible interactions. Ligand-receptor pairs without identified interacting spots were excluded and only experimentally validated pairs were retained according to ConnectomeDB2025 database [38]. The resulting gene pairs were subsequently used for additional receptor-ligand analyses based on spatial gene expression. Specifically, weighted networks, ligand receptor network, and ligand target matrix were downloaded from nichenetr package [39] database. Downstream target genes were predicted using pooled of differentially expressed genes between control and COPD within respective niche and across niches. Ligand activities were performed using downstream target genes, background genes from all detected genes, the ligand-target matrix, and spatially relevant ligands. The resulting ligand activity matrix was filtered to exclude non-significant ligands (corrected area under the precision-recall < 0). Ligand receptor network table was subset further to retain only spatially relevant ligand-receptor pairs, and functionally relevant, literature supported ligand-receptor pairs with secreted ligands were selected and visualised using circos plot modified from circlize package [40].

#### Transcription factor network

List of transcription factor genes and their associated target genes were downloaded from Dorothea [41] and OmnipathR package [42] using *dorothea*() function with following parameters: resources: (‘DoRothEA’, ‘TFactS_DoRothEA’,“TFactS_CollecTRI”), organism: 9606, and Dorothea_levels: (‘A’, ‘B’). The TF table was filtered to include unique pairs of TF and target genes, which were subset to retain ligand genes, shown in Fig. 3C. Visualisation of network was performed using igraph package [43].

#### Gene expression correlation

Integrated scRNA-seq Seurat object was subset to AT2 cells. Gene expression of AT2 cells was normalised and only cells from COPD lungs were included and aggregated by individual lung. Pseudo-bulk gene expressions of FOS and FAS were correlated using pearson method and visualised using ggplot2 package.

### Open access GeoMx Nanostring spatial transcriptomics dataset

#### Cohort and data analysis

A workflow of data analysis steps is available in Supplementary Figure S3. Gene expression of GeoMx Nanostring spatial transcriptomic datasets (GSE292993) [22] were downloaded and subset to include airway and parenchyma region of interests. This dataset consisted of control (*n* = 16) from patients undergoing lung resection for a peripheral nodule and advanced COPD (*n* = 10) lungs from patients who either underwent lung transplantation or lung volume reduction surgery (Table S4), as also described previously [22].

#### Quality control

ROIs with low reads (raw sequencing reads < 1000, % aligned reads < 80%, % trimmed reads < 80%, % stitched reads < 80%, % sequencing saturation < 50%), low negative probes (<1), low number of detected genes (<1%), and small ROI areas (<1000) were excluded. Feature genes above the limit of quantification in ≥10% of the samples were retained as reported elsewhere [22].

#### Data transformation, normalization, and dimensionality reduction

Filtered gene expression data was subjected to normalization performed in GeoMxTools package [44] and batch corrected for processing time and fixation methods as outline in the supplementary materials of the original publication [22]. Batch corrected values were log-transformed and used for downstream analysis. Principal component analysis was conducted using scater package [45] using transformed data, visualised with ggplot2 package, and coloured by variables (patient ID, disease group) to examine any outliers.

#### Gene set enrichment analysis

Log-transformed, batch-corrected gene expression values and list of top 100 driving genes (Fig. S9) of all niches were used to run gene set enrichment analysis. Driving genes were subset to exclude ribosomal genes and retain only those detected in the GeoMx data. The resulting enrichment score matrix was subjected to further filtering by removing ROIs with negative enrichment scores and outliers based on PCA.

#### Differential expression analysis

Differentially expressed genes were calculated using log-transformed gene expression values using linear mixed model applied in lmerTest package [46], with the following formulas: 1) gene expression ~ ROI group+(1|individual sample), 2) gene expression ~ disease+(1|individual sample). Significance was adjusted with FDR method and estimated difference values were plotted against p_adj_ as volcano plot using ggplot2 package.

#### Pathway enrichment analysis

Differentially expressed genes (p_adj_ ≤ 0.1) between parenchyma of COPD and control were used as input to calculate significantly enriched pathways using PathfindR R package with gene ontology of biological process as a reference gene set [47]. This package identifies enriched pathways by leveraging protein-protein interaction networks and considers the connectivity of deregulated genes and prioritizes clusters of moderately significant, functionally related genes rather than relying solely on individual gene overlap. Only significantly enriched pathways (p_adj_ ≤ 0.05) were subsequently compared with deregulated pathways identified in the Visium parenchyma dataset.

#### Region of interests (ROIs) deconvolution

Reference matrix was created from integrated control and COPD scRNA-seq dataset by subsetting gene expression of 200 cells per cell type. Feature genes of scRNA-seq dataset were filtered to only include detected genes from GeoMx dataset. Normalised, batch corrected values were used and any cells with zero gene expression were removed. The reference matrix with genes as rows and cell identities as columns was uploaded to CIBERSORTx platform [48]. Signature matrix was generated with the following parameters: kappa of 999, q-value of 0.01, 300–500 barcode genes, min. expression of 1, replicates of 5, and sampling of 0.5 and not filtering non-haematopoietic genes. Log-transformed, batch-corrected gene expression from all ROIs was uploaded as mixture matrix. Together with signature matrix, mixture matrix was utilised to estimate cellular proportions within each ROI with the following parameters: enabled batch correction, batch correction S-mode, absolute run mode, and 100 permutations. Estimated scores were downloaded, normalised with log_10_(proportion+0.00001), referred to cell-type-associated transcript abundance, and subjected into differential abundance analysis with linear mixed model applied in lmerTest package with formula for a given cell type: cell percentage ~ ROI group+(1|individual sample).

### Statistical analysis

Data visualisation and statistical analysis were performed with R v4.4.1 (using the packages tidyverse [49], RColorBrewer [50], ggplot2 [51], ggpubr [52], ggrepel [53], pheatmap [54], dendsort [55], emmeans [26], lmertest [46], ggplotify [56], rstatix [57], as stated at the respective sections. The statistical details including test and *p* values are described in the figures and the corresponding figure legends.

## Results

### Spatial transcriptomics of healthy and COPD lungs revealed distinct transcriptional profiles across lung compartments

**Fig. 1:**
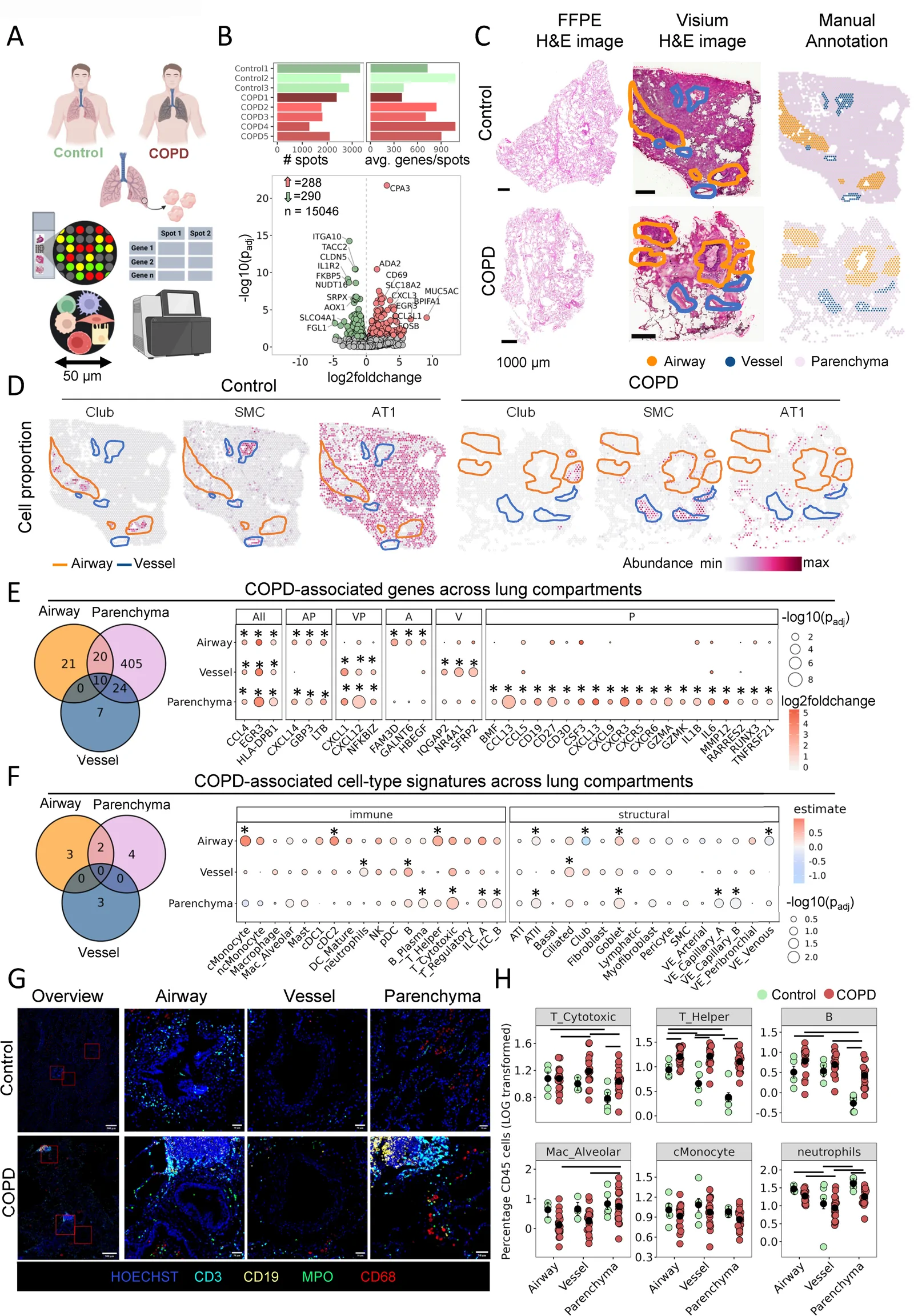
Immune and tissue remodelling response characterize COPD compartments (**A**) Study workflow schematic. Fresh frozen lung sections from unused downsized healthy donor (control) and explanted COPD lungs were placed onto oligonucleotides-layered Visium slides (50-µm capture areas). (**B**) Comparison of spot numbers and average genes per spot across samples. A volcano plot shows differential gene expression between COPD and controls based on pseudobulk expression across all spots. Colours indicate individual samples. Significantly deregulated genes (p_adj_ ≤ 0.1, Benjamini–Hochberg adjusted) are highlighted, with the top 10 genes labelled. (**C**) H&E images of formalin fixed paraffin embedded (FFPE) and spatial sections with corresponding spatial maps of manually annotated lung compartments: airway, vessel, and parenchyma. Airway and vessel regions are marked in orange and blue. (**D**) Spatial maps of deconvoluted structural cell abundances in representative control and COPD samples, AT1 = alveolar type 1 cells, SMC = smooth muscle cells. Colour indicates deconvolution score; airway and vessel regions are outlined. (**E**) Venn diagram showing significantly upregulated genes in COPD compared to controls across lung compartments (p_adj_ ≤ 0.1, log2 Fold change > 0). A dot plot displays selected upregulated genes, with colour representing log2 Fold change and dot size indicating statistical significance. (**F**) Venn diagram of significantly increased cell associated transcript abundance in COPD across compartments (p_adj_ ≤ 0.1), identified using linear mixed models with individual samples as random effects and diagnosis and compartment as fixed effects, followed by FDR correction. The accompanying dot plot shows estimated transcript abundance differences of immune and structural cell types between COPD and controls. A, P, and V denote airway-, parenchyma-, and vessel-specific genes, respectively; AP and VP indicate shared compartments; *p_adj_ ≤ 0.1. (**G**) Representative multiplex immunofluorescence images of neutrophils (MPO^+^), B cells (CD19^+^), T cells (CD3^+^), and macrophages (CD68^+^) across compartments in control and COPD lungs. Scale bar is 50 µm. (**H**) Flow cytometry comparing log-transformed relative proportions of immune cell populations within CD45^+^ cells between COPD and controls across compartments. Dots and error bars represent estimated means ± standard errors; lines indicate p_adj_ ≤ 0.1

To generate spatially resolved whole transcriptome signatures, we applied 10x Visium spatial transcriptomics on fresh frozen lung samples from end-stage COPD lungs (*n* = 5) and healthy donor lung tissue as controls (*n* = 3) (Fig. 1A-B, Table 1). Reference FFPE sections showing typical COPD pathology are shown in Fig. 1C and S4A and described in Table 1. On average, 2500 spots (range: 1314–3604) per lung section were analysed with ~700 genes (range: 80–5432) sequenced per spot (Fig. 1B), resulting in ~97–216 million sequencing reads per lung. The spatial distribution of the number of unique genes sequenced and library size was comparable across macroscopically detected lung structures (Fig. S4B-D). Initial comparison of the aggregated gene expression between COPD and control lungs, identified increased expression of genes coding for metalloproteases (*CPA3*), mucosal immunity (*MUC5AC*, *BPIFBA1*), immune-cell-homing protein and T cell activation (*CD69*), as well as chemokines (*CXCL3*, *CCL3L1*), indicating the increase of immune recruitment and responses in COPD. Genes with decreased expression in COPD included those encoding tight junction proteins (*CLDN5*), DNA repair proteins (*NUDT16*), and xenobiotic metabolizing proteins (*AOX1*) (Fig. 1B)

To provide structural context to the gene signatures, we manually annotated the associated H&E-stained images highlighting known macroscopical structures, additionally using scRNA-seq derived gene markers for guidance (Fig. 1C, S4E-G). Cell-type-associated transcript abundance within each spot was then deconvoluted based on reference scRNA-seq gene derived from control and COPD lungs (GSE171541 [19] and GSE136831 [20], Fig. S5A-B). Cellular deconvolution revealed the expected correlation of the manual annotation labels with structural cell inferred abundance (Fig. 1D S5C-D).

### COPD compartments exhibited distinct tissue remodelling signatures and immune cells infiltration

We next sought to understand how the cellular response in COPD potentially differs according to structural compartment. Compared to controls, COPD parenchyma exhibited the highest number of upregulated genes (*n* = 459), followed by airways (*n* = 51), and vessels (*n* = 41) (Fig. 1E). Parenchyma showed an exclusive upregulation of genes associated with immune activation (*CD27*), recruitment (*CXCR3*, *CXCR6*), and tissue remodelling (*MMP12*, *GZMA, GZMK*) (Fig. 1E). In contrast, airway exclusively upregulated genes associated with increasing mucin-type O-glycosylation (*GALNT6*) and growth factor (*HBEGF*), while vessels upregulated Wnt signalling modulator (*SFRP2*). Interestingly, all compartments shared an upregulation of chemoattractant (*CCL4*) and antigen presentation genes (*HLA-DPB1*) (Fig. 1E). The comparison of deconvoluted structural and immune cell types exhibited distinct regulation patterns across lung compartments (Fig. 1F). Compared to control groups, COPD airways possessed more helper T cells, cDC2, and classical monocytes, but decreased club cells. While the relative abundance of B cells and neutrophils were increased in COPD vessels and the proportion of cytotoxic T and plasma B cells was significantly higher in the COPD parenchyma (Fig. 1F).

Cell type-associated transcript abundance was then validated on the protein level via immunofluorescence and flow cytometry (Cohort demographics Tables 2 and 3). Multi-colour immunofluorescence revealed marked CD3^+^ T and CD19^+^ B cells infiltration in the COPD parenchyma (Fig. 1G, S6A-B). Consistent with our deconvolution and immunofluorescence results, flow cytometry of freshly isolated lung compartments (Fig. S7A) revealed that the proportion of cytotoxic T and B cells was increased in parenchymal tissue, while helper T cells were enriched in all compartments (Fig. 1H, S7B). In the airway, the abundance of macrophages and mast cells were inversely correlated with diffusion capacity (DLCOcSB). Airway macrophage abundance increased with age, while in the parenchyma, macrophage numbers correlated with smoking exposure (Fig. S7C). Altogether these results indicate distinct chemokine and tissue remodelling gene signatures within COPD compartments. The abundance of specific immune cells also suggests the ongoing, persistent recruitment and activation of the inflammatory response and tissue remodelling in advanced COPD (Fig. S8).

### Identification of lung spatial niches

To identify distinct microenvironments with similar underlying transcriptional patterns we applied non-negative matrix factorization (NMF) [58]. All spots could be broadly assigned to one of the 20 identified niches (NMF_1-20), each niche characterized by distinct patterns of key driving genes (Fig. S9). Individual niches were associated with both manual annotations (Fig. S10A) and cellular deconvolution results (Fig. S10B). Niches assigned to the same tissue compartment showed similar transcriptional patterns (Fig. S10C), reflecting core gene expression signatures. Specifically, NMF_7, NMF_19, and NMF_20 were linked to airway compartments; NMF_13 and NMF_14 were vascular-related; and the remaining 15 niches were associated with the lung parenchyma (Fig. S10B-C). To ensure each spatial spot associated with a single niche, we focused on the most active regions, which displayed the highest scores for a given niche (above the 95th percentile) and across all niches (Fig. S11A-B).

### Identification of emphysema-associated parenchymal niches linked to macrophage, stress, and immune responses

**Fig. 2:**
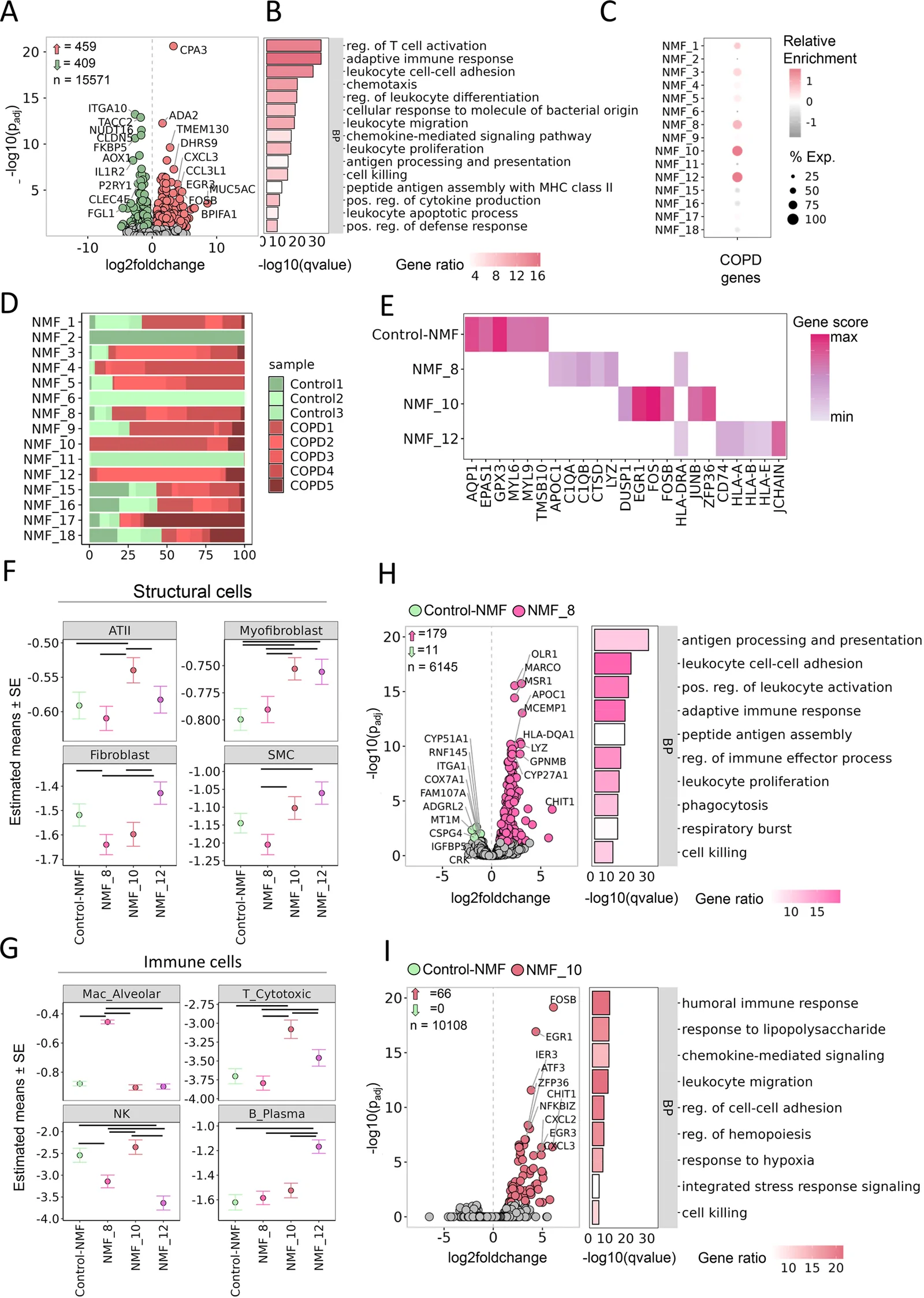
Parenchymal remodelling-stress niche is defined by stress responses and increased AT2, NK, and cytotoxic T cells. (**A**) Differentially expressed genes between COPD and control parenchyma. Genes from all parenchymal regions were aggregated and used for differential expression analysis. Significantly deregulated genes (p_adj_ ≤ 0.1) are highlighted, with the top 10 most significant genes labelled. (**B**) Pathway enrichment analysis of COPD parenchyma. Enriched pathways were identified from significantly upregulated genes in COPD parenchyma (p_adj_ ≤ 0.1, log₂ Fold change > 0) using the clusterProfiler R package. Only the most significant pathways (qvalue ≤ 0.1) are shown. BP denotes gene ontology biological process. (**C**) Enrichment of COPD-associated genes across parenchymal niches. Scores for upregulated genes in COPD parenchyma were calculated using *AddModulescore()* in Seurat. Dot size indicates the proportion of niche spots expressing the score, and colour represents relative enrichment scores across niches. (**D**) Proportion of selected parenchymal niches across individual control and COPD samples, normalized to total parenchymal area per sample. Colours indicate individual samples. (**E**) Top driving genes defining selected parenchymal niches. Colour indicates gene scores within each niche. NMF_2, NMF_6, and NMF_11 were combined to generate a reference control-NMF. (**F**) Quantification of selected structural and (**G**) immune cell associated transcript abundance across control-NMF and emphysema-associated niches. Significance was assessed using linear mixed models with individual samples as random effects and diagnosis and niche as fixed effects. *p*-values were adjusted using FDR; results are shown as estimated means ± standard errors, with lines indicating p_adj_ ≤ 0.1. (H–I) differential gene expression between the macrophage-enriched niche (NMF_8) or remodelling-stress niche (NMF_10) and control-NMF. Differential expression was performed on pseudobulk data using DESeq2 with Wald tests and Benjamini–Hochberg correction. Significant genes (p_adj_ ≤ 0.05) are highlighted, with the top 10 labelled. Pathway enrichment of upregulated genes was performed using clusterProfiler, with significance defined as qvalue ≤ 0.05

We first focused on the lung parenchyma and performed differential gene expression analysis comparing COPD and controls. This resulted in 459 upregulated genes enriched for pathways including adaptive immune response and T cell activation, and 409 downregulated genes associated with cell adhesion and wound healing (Fig. 2A–B, S12A). To validate these global transcriptional changes, we compared our results with a publicly available GeoMx Nanostring spatial transcriptomic parenchyma dataset (Cohort details Table S4) [22, 59]. Relative to non-disease controls, COPD patients possessed significantly higher degree of emphysema and airflow limitation while no difference in smoking status (Fig. S13A). Comparison of controls and COPD samples and identification of highly connected deregulated genes revealed a partial overlap in the resulting enriched pathways (Fig. S13B-E) with protein phosphorylation, regulation of inflammatory response, T cell co-stimulation, and response to interleukin-1 emerging as consistently deregulated processes.

Continuing with our higher resolution spatial dataset, we determined the specific localization of the transcriptional changes (Fig. 2A–B) within the spatially identified niches. Control-associated gene and pathway signatures were primarily enriched in three niches (NMFs_2/6/11, Fig. S12A-C). As NMF_2/6/11 were exclusively found in control samples (Fig. 2D) and shared high similarity of underlying driving genes as well as inferred cell types (Fig. S10C), these niches were combined to create one control reference niche, termed Control-NMF, and thereby simplify downstream COPD comparisons (Fig. S12D). Control-NMF was characterized by genes associated with water transport (AQP1), antioxidant activity (GPX3) (Fig. 2E, S12D), that correlated with the abundance of AT1 and AT2 cells (Fig. S14A)

In contrast, the strongest COPD gene score enrichment localised to NMFs_8/10/12 (Fig. 2C). These niches were either strongly enriched (e.g. NMF_8) or exclusive to COPD samples (NMFs_10/12; Fig. 2D). NMF_8 was driven by genes associated with complement activation (C1QA), which positively correlated with alveolar macrophage abundance; NMF_10 by stress and tissue remodelling genes (FOS, EGR1) and correlated with structural cells i.e., AT2, AT1 cells, myofibroblasts; and NMF_12 by immunoglobulin genes (JCHAIN), associated with immune cells e.g. plasma B cells and cDC2 (Fig. 2E, S14A). Therefore, we assigned these NMFs_8/10/12 as macrophage-enriched, remodelling-stress, and humoral immune niches, respectively.

Pathways analysis based on key driving genes revealed shared and niche-specific processes. While control-NMF was specifically enriched for transepithelial and gas transport, T cell activation and adhesion were observed across control- and emphysema-associated parenchymal niches with stronger enrichment in macrophage-enriched (NMF_8) and humoral immune (NMF_12) niches. In contrast, NMF_8 and the remodelling-stress niche (NMF_10) exhibited exclusive enrichment in receptor-mediated endocytosis and integrated stress signalling (Fig. S14B), respectively, indicating aberrant inflammatory and structural remodelling response within distinct COPD parenchymal regions.

### Disease associated cellular and molecular signatures within emphysema-associated parenchymal niches

To assess disease-associated cellular changes in the parenchyma, we compared the cellular deconvolution results. Relative to the Control-NMF, the remodelling-stress niche (NMF_10) cellular deconvolution revealed significantly higher proportion of inferred cytotoxic T, NK, and AT2 cells. A notable inferred-abundance of alveolar macrophage and plasma B cells was observed in macrophage-enriched (NMF_8) and humoral immune niche (NMF_12), respectively (Fig. 2F–G, S15A-B). DEG analysis on aggregated gene expression revealed that macrophage-dominated areas were enriched in antigen presentation pathways, as well as phagocytosis and respiratory burst (Fig. 2H) relative to controls. The remodelling-stress and humoral immune niches both showed upregulation of humoral immune responses and chemokine signalling; however, the remodelling-stress niche was characterized by enriched response to hypoxia, whereas the humoral immune niche demonstrated upregulation of MHC class II assembly and lymphocyte proliferation (Fig. 2I, S16A-C). Together, these findings highlight the heterogeneity of inflammatory responses with shared and unique cellular composition and signalling pathways within distinct parenchymal areas of COPD.

### Cytotoxic T cells colocalised with AT2 cells and are linked to AT2 stress response in parenchymal COPD

**Fig. 3:**
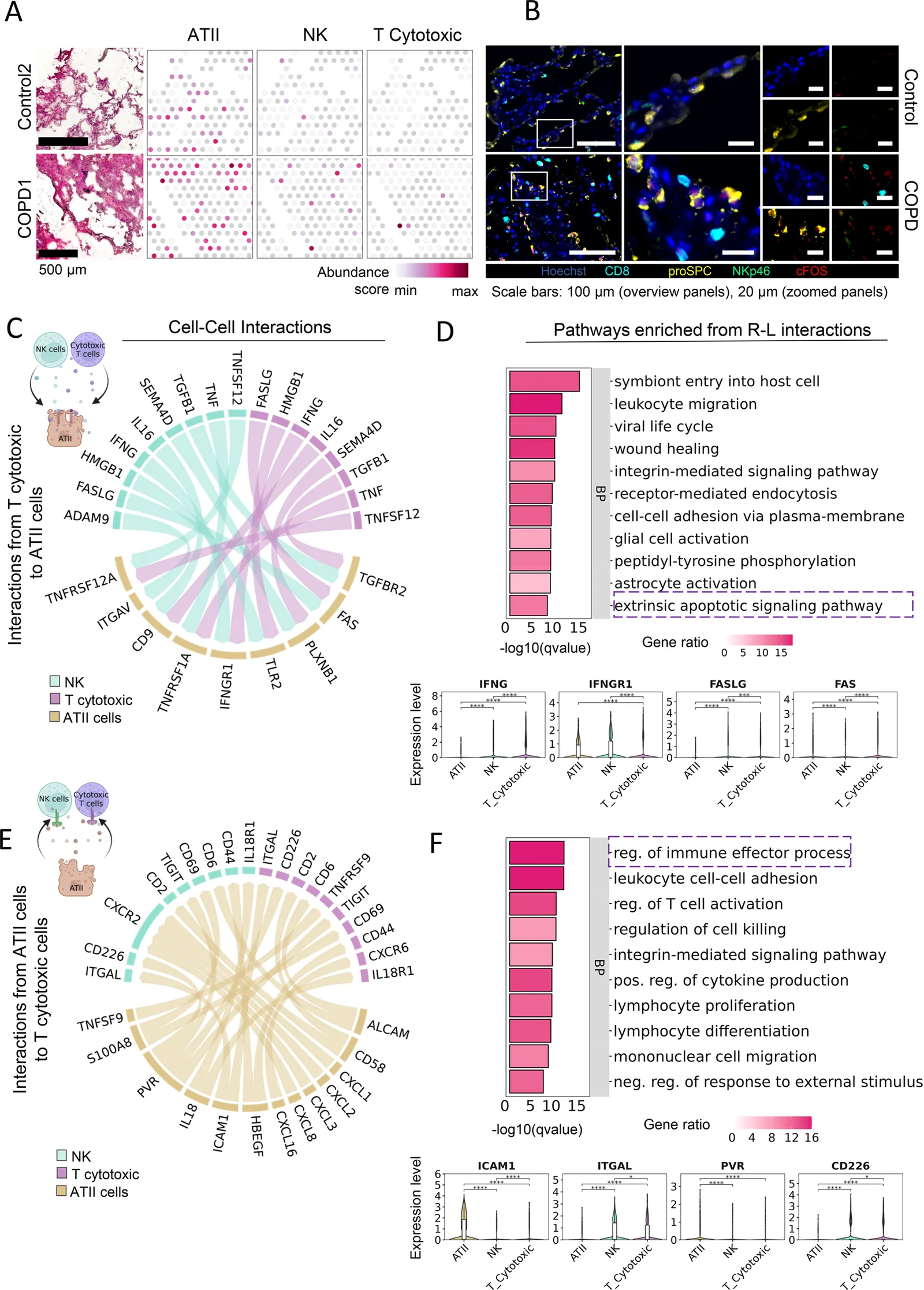
Cytotoxic T cells colocalise with AT2 cells, linking immune activation to stress responses in COPD parenchyma (**A**) Zoomed H&E images and spatial maps showing cell type associated transcript abundance of AT2, NK, and cytotoxic T cells within the remodelling-stress niche (NMF_10) in control and COPD lungs. Colour indicates deconvolution score. (**B**) Multiplex immunofluorescence staining of FOS (cFOS^+^, red) in AT2 cells (ProSPC^+^, yellow), cytotoxic T cells (CD8^+^, turquoise), and NK cells (NKp46^+^, green) within parenchyma of control and COPD lungs. Representative images from 3 controls and 3 COPD samples are shown. Scale bars: 100 µm (overview) and 20 µm (zoom). (**C**) Ligand–receptor interactions inferred using the nichenetr R package on integrated control and COPD scRNA-seq datasets (GSE136831, GSE171541). Circos plot showing prioritised ligands expressed by NK or cytotoxic T cells and their corresponding receptors on AT2 cells. Ligand–receptor gene pairs were filtered to retain spatially colocalised interactions involving secreted ligands within the remodelling-stress niche. Functionally relevant, literature supported ligand-receptor gene pairs were visualised. Colours indicate cell types; directed edges represent signalling direction from sender to receiver. (**D**) Bar plot of significantly enriched pathways (qvalue ≤ 0.05) derived from receptors and target genes identified in (**C**). Colour represents the ratio of expressed genes to gene set size, and the x-axis indicates significance. BP denotes gene ontology biological process. Violin plots show RNA expression of selected ligand or receptor genes across AT2 and cytotoxic T cells in COPD samples. Significance was assessed using Wilcoxon tests with FDR correction (* p_adj_ ≤ 0.05, ** p_adj_ ≤ 0.01, *** p_adj_ ≤ 0.001, **** p_adj_ ≤ 0.0001). (**E**) Circos plot of ligand–receptor interactions with AT2 cells as signal senders and NK or cytotoxic T cells as receivers, inferred using nichenetr and filtered for spatially colocalised within the remodelling-stress niche. (**F**) Enriched pathways (qvalue ≤ 0.05) derived from receptors and target genes identified in (**E**), with corresponding violin plots showing expression of selected genes across AT2 and cytotoxic T cells in COPD

In the remodelling-stress niche (NMF_10), cell-type-associated transcript abundance of AT2 cells were spatially colocalised with NK and cytotoxic T cells (Fig. 3A, S17A-C), a pattern independently confirmed by immunofluorescence showing pronounced colocalisation among FOS^+^ cytotoxic T and FOS^+^ AT2 cells (Fig. 3B, S17D). We next examined potential receptor-ligand interactions between AT2 and cytotoxic T cells using the combined scRNA-seq control and COPD dataset (GSE171541, GSE136831). Multiple prioritisation steps were applied to ensure the spatial context and biological relevance of the identified receptor-ligand pairs. Specifically, only receptor-ligand genes that were spatially colocalised and predicted to interact within NMF_10 were retained, defined as being located within a 200 µm radius from each other (Fig. S18A). Candidate pairs were further refined based on a curated database of experimentally validated interactions [39] and only spatially validated, functionally relevant, literature supported interactions involving secreted ligands were visualised (Fig. S18B-C).

Cytotoxic T cells were predicted to interact with AT2 cells via *IFNG*-*IFNGR1* and *FASLG*-*FAS*, which are connected to extrinsic apoptotic signalling pathway (Fig. 3C–D). In COPD lungs, *IFNG* and *FASLG* were significantly upregulated in NK and cytotoxic T cells relative to AT2 cells (Fig. 3D). Interestingly, *FAS* expression was regulated by *FOS* and positively correlated with FOS expression in AT2 cells (Fig. S18D-E). To substantiate the predicted receptor-ligand pairs we performed a proof-of-concept validation experiment where human epithelial A549 cells were co-cultured with isolated CD8^+^ cytotoxic T cells. Activated CD8^+^ T cells produced IFN-γ, and both direct IFN-γ stimulation or epithelial/CD8 cell co-culture led to rapid activation of the key IFN-γ signalling protein STAT1, which was strongly reduced in the presence of an IFN-γ blocking antibody (Fig. S19A-C).

Interactions from AT2 cells to cytotoxic T cells revealed genes and pathways associated with immune activation and recruitment. Certain AT2 ligands such as *ICAM1* and *PVR* were predicted to bind to cytotoxic T cells receptors including *ITGAL and CD226,* respectively (Fig. 3E). Pathway analysis on receptor, ligand, and target genes (Fig. S18F-G) revealed that *ITGAL* and *CD226* were highly expressed by NK and cytotoxic T cells compared to AT2 cells and associated with immune effector process and cell-cell adhesion (Fig. 3F). Together these findings highlighted the colocalisation and potential interactions between cytotoxic T, NK, and AT2 cells in promoting parenchyma remodelling and inflammatory response in COPD lungs.

To further investigate whether similar interactions may also be observed in earlier disease stages, we additionally analysed a third open-access scRNA-seq dataset (GSE173896 [18]) consisting of non-COPD controls (*n* = 7) and GOLD1-2 COPD (*n* = 9) from patients undergoing lung resection. The resulting receptor-ligand analyses identified similar interactions and pathways (Fig. S20A-C).

### Perivascular remodelling with myofibroblast expansion and ECM reorganization characterizes COPD vessels

**Fig. 4:**
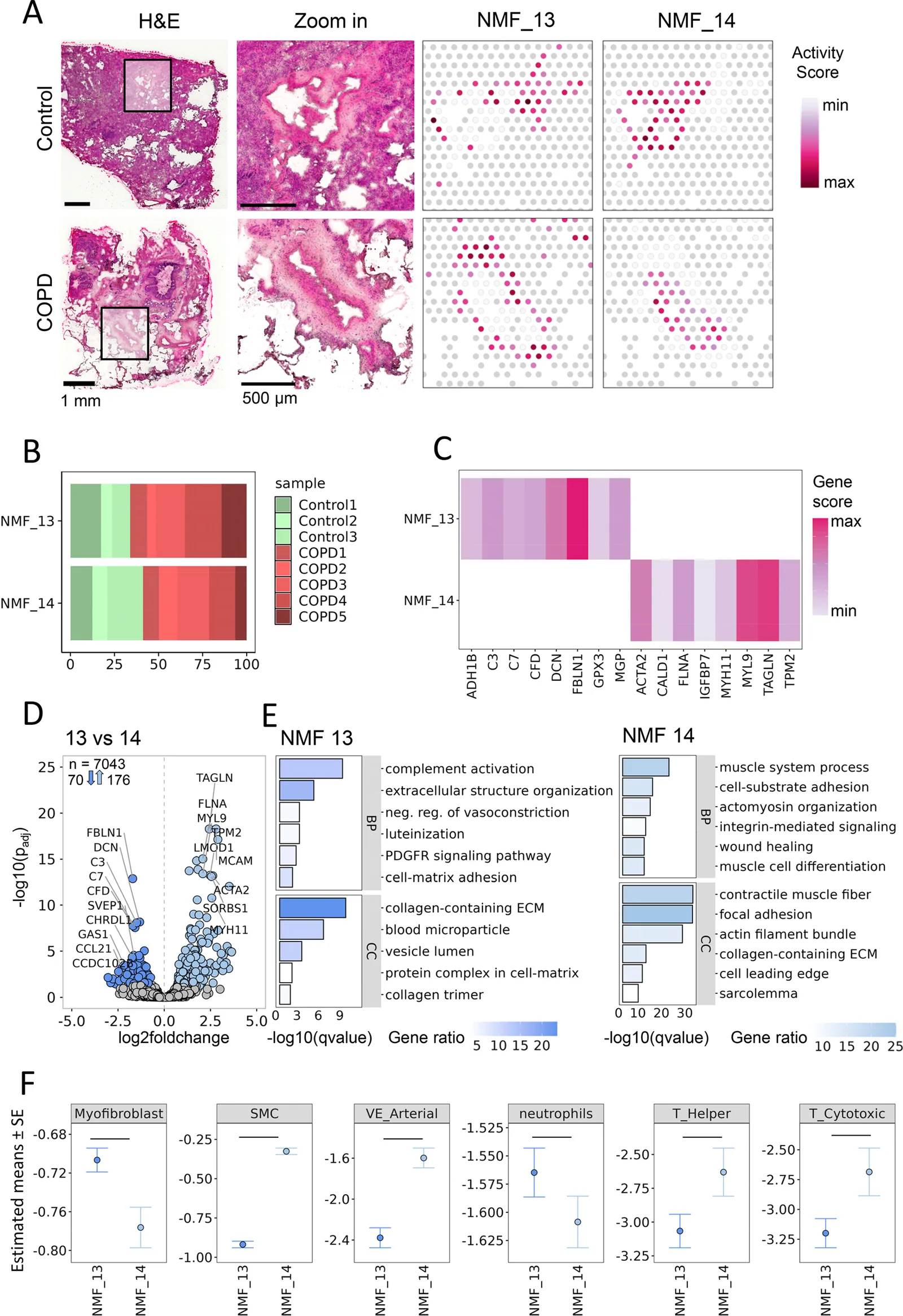
Perivascular myofibroblast expansion and ECM remodelling characterize COPD perivascular niche (**A**) Spatial map of vessel niches. Left panels showing full H&E image of lung sections and zoomed H&E image of representative vessels. Right panels depicting the localization of the niches on selected donor and COPD vessels. Colour represents the enrichment score of a given niche. (**B**) Bar chart showing the proportion of vessel niches across individual control and COPD samples relative to total vessel areas in a given sample, colour represents individual sample; NMF_13: perivascular niche, NMF_14: vascular niche. (**C**) Top driving genes underlying vessel niches; colour represents gene loading scores. (**D**) Differentially expressed genes across vessel niches. Gene expression was aggregated and grouped by individual samples and niches. Differentially expressed genes was calculated using DESeq2 R package. Only significant genes (p_adj_ ≤ 0.05), adjusted by Benjamini–Hochberg method, were coloured and top 10 genes based on significance and Fold change were labelled. (**E**) Bar plot of selected significantly enriched pathways (qvalue ≤ 0.05) of perivascular and vascular niches. Pathway enrichment analysis for each niche was performed on genes from (**D**), using clusterProfiler R package; BP: gene ontology biological process, CC: gene ontology cellular component. (**F**) Quantification of selected structural and immune cell associated transcript abundance across perivascular and vascular niches. Significance was calculated with linear mixed model with individual sample as a random effect and niches as a fixed effect. *p*-values were adjusted with FDR; lines indicate p_adj_ ≤ 0.05. Data was represented with estimated means ± standard errors

Our investigation into vascular regions revealed two niches aligned with manually annotated vessel areas: NMF_13 (perivascular) and NMF_14 (vascular) (Fig. 4A–B). The vascular niche contained genes related to contractility (*MYL9*, *TAGLN*) and was associated with SMC, while the perivascular niche was driven by ECM component genes (*DCN*, *FBLN1*) and associated with fibroblasts (Fig. 4C, S21A). Key driving pathways revealed shared upregulation in supramolecular fibre organization along with niche-specific pathways—regulation of peptidase in the perivascular and muscle system process in the vascular niche (Fig. S21B).

Transcriptional comparison between the two vascular niches confirmed the enrichment of extracellular structure organization in perivascular and muscle system process within vascular niche (Fig. 4D–E). Composition of deconvoluted cell types were shifted, including increased fibroblast and myofibroblast, associated with collagen containing ECM in perivascular region while an increase in SMC and arterial endothelial cells (VE_Arterial) associated with contractile muscle fibre and cell adhesion processes in vascular spots (Fig. 4F, S22–S23). Compared to controls, perivascular regions in COPD vessels exhibited significantly increased myofibroblast and neutrophils, and a higher number of DEGs (*n* = 62), including genes related to regulation of apoptosis (*NR4A1*) and peptidase (*PI16*) activity, with enrichment of chemokine signalling and collagen containing extracellular matrix (Fig. S24–S25). These changes were not observed in vascular niche, suggesting prominent immune-fibroblasts/myofibroblast crosstalk, and ECM remodelling in perivascular compartment in COPD.

### Specific airway niches define COPD and control airways

**Fig. 5:**
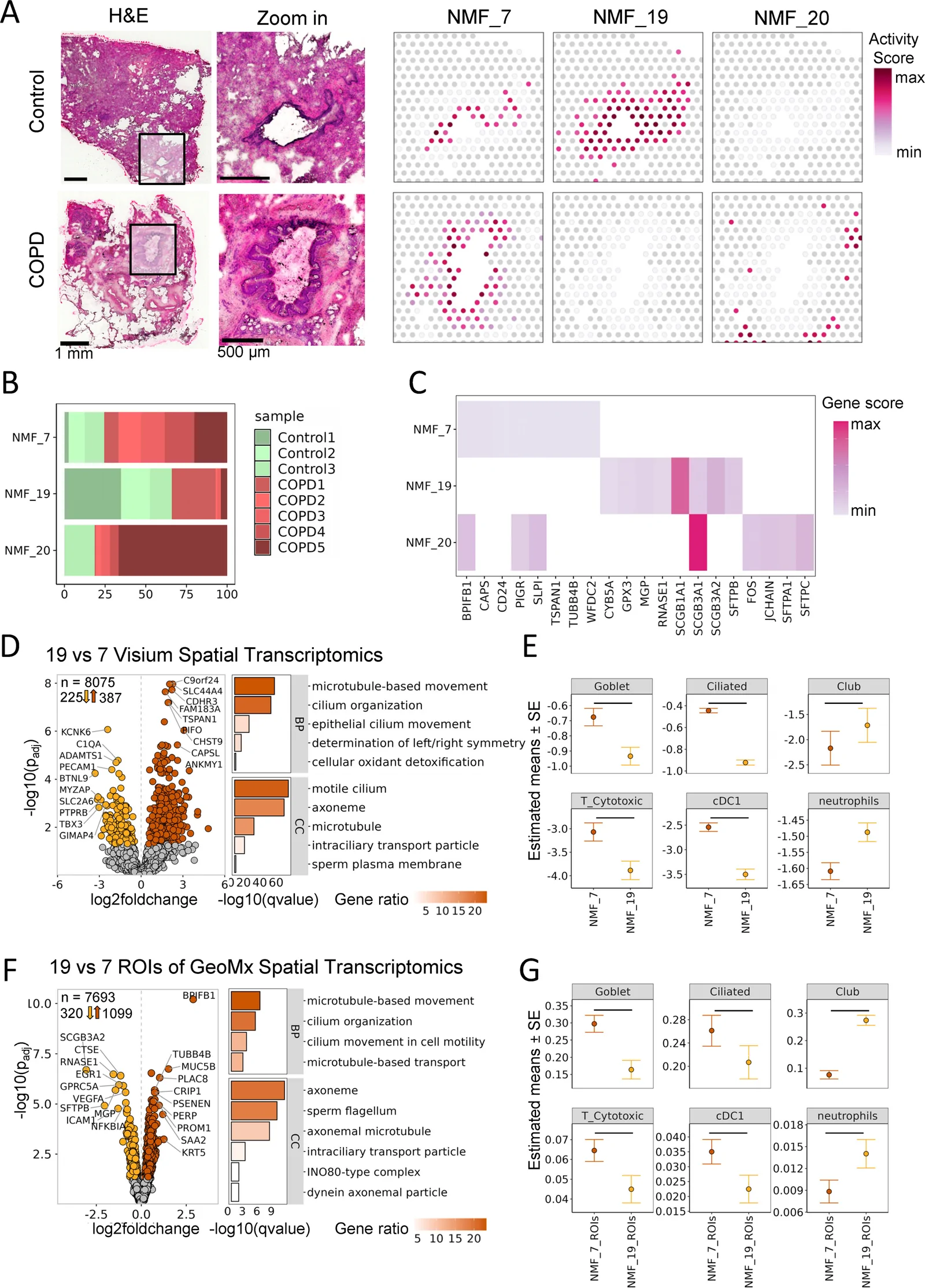
COPD endobronchial niche shows skewed transcriptional regulation toward epithelial differentiation with pronounced cytotoxic T–goblet associations. (**A**) Spatial maps of airway-associated niches. Left panels show zoomed views of representative airways. Right panels depict niche localization in selected control and COPD airways, with colour indicating niche enrichment scores. (**B**) Proportion of airway niches across individual control and COPD samples, normalized to total airway area per sample. Colours represent individual samples. NMF_7 denotes the COPD endobronchial niche, NMF_19 the control endobronchial niche, and NMF_20 the COPD peribronchial niche. (**C**) Top driving genes defining airway niches, with colour indicating gene loading scores. (**D**) Differential gene expression between COPD and control endobronchial niches. Gene expression was aggregated by individual samples and niches, and differential expression analysis was performed using DESeq2 with Benjamini–Hochberg correction. Significantly deregulated genes (p_adj_ ≤ 0.05) are highlighted, with the top 10 genes labelled. Bar plots show significantly enriched pathways (qvalue ≤ 0.05) for the COPD endobronchial niche, identified from upregulated genes using clusterProfiler. BP and CC denote gene ontology biological process and cellular component, respectively. (**E**) Quantification of selected structural and immune cell associated transcript abundance across airway niches. Significance was assessed using linear mixed models with individual samples as random effects and niche as a fixed effect. *p*-values were adjusted using FDR, and data are shown as estimated means ± standard errors; lines indicate p_adj_ ≤ 0.05. (**F**) Differential gene expression between airway ROIs enriched for COPD or control endobronchial signatures, derived from GeoMx NanoString spatial transcriptomics data. ROIs were classified based on enrichment scores, and differential expression was assessed using linear mixed models. Significant genes (p_adj_ ≤ 0.05) are highlighted, with pathway enrichment shown for upregulated genes. (**G**) Quantification of selected structural and immune cell associated transcript abundance across airway ROI groups using linear mixed models with FDR correction

Analysis of the airway-associated niches (NMF_7, NMF_19, and NMF_20) revealed distinct spatial patterns: NMF_7 and NMF_19 were predominantly located adjacent to the airway lumen, whereas NMF_20 was enriched in the surrounding airway tissue (Fig. 5A). The relative abundance of these niches also varied by disease state, with NMF_7 and 20 being more prevalent in COPD samples and NMF_19 enriched in controls (Fig. 5B). Based on their localization and COPD-specific distribution, we designated NMF_7 as COPD-endobronchial, NMF_20 as COPD-peribronchial, and NMF_19 as control-endobronchial.

The top contributing genes in the COPD-endobronchial (NMF_7) niche were involved in innate host defence (*BPIFB1* (BPI fold-containing family B member 1), *SLPI* (Secretory Leukocyte Peptidase Inhibitor), which highly correlated with the presence of goblet and ciliated cells (Fig. 5C, S26A). In contrast, control-endobronchial (NMF_19) spots were characterised by high expression of secretoglobins (*SCGB3A2*, *SCGB1A1*) and antioxidant-related genes (*CYB5A*), associated with club cells. COPD-peribronchial (NMF_20) spots were strongly associated with antibody secretion (*JCHAIN*) and plasma B cells. The expression of these top markers decreased rapidly with increasing distance from the airway lumen (Fig. S26B). Notably, four COPD samples exhibited elevated *BPIFB1* expression within the airways (Fig. S26B), reflecting a disease-associated, spatial transcriptional reprogramming of this gene within the COPD-endobronchial area.

Airway niches exhibited both distinct and shared signature pathways (Fig. S26C). The COPD-endobronchial niche was enriched with cell killing and epithelial differentiation processes, while the control-endobronchial niche was enriched in oxidative stress response and plasma membrane organization. Chaperone-mediated autophagy was consistently enriched across all niches. Together, these findings reveal the presence of disease specific endobronchial areas: a healthy endobronchial region, defined by club cell-associated genes and enrichment of antioxidant processes, and COPD endobronchial regions characterized by genes correlated with the abundance of goblet cells and enrichment of cell killing and epithelial differentiation processes, as well as humoral immune response (Fig. S26C).

### Independent spatial transcriptomic cohort validated the presence of endobronchial niches

To investigate the spatial differences in the transcriptome, we performed differential expression analysis between different airway niches. COPD-endobronchial (NMF_7) exhibited an upregulation in genes related to cilia formation (*C9orf24/CBE*), control-endobronchial (NMF_19) upregulated genes associated with epithelial ion transport (*KCNK6*) (Fig. 5D). Pathway analysis supported the enrichment of cilium movement and organization in COPD-endobronchial and cell junction in control-endobronchial (Fig. 5D, S26D).

In the COPD-endobronchial niche, the cell type-associated transcript abundance of goblet, ciliated, cytotoxic T cells, and cDC1 were significantly higher, while control-endobronchial was dominated by club cells and neutrophils (Fig. 5E, S27). We next sought to tissue organization with the cellular responses by associating the dominating cell types in each region with the pathway analysis results. Goblet cell-associated transcript abundance positively correlated with COPD-endobronchial associated pathways including epithelial cell differentiation but not to control-endobronchial niches pathways, while club cells and neutrophils showed a positive correlation with control-endobronchial pathways including actin organization and surfactant homeostasis (Fig. S28), highlighting the spatial association of specific immune and structural cell types within a specific airway niche.

To validate our airway niche results, we reanalysed GeoMx Nanostring spatial transcriptomic airway dataset on controls and COPD lungs (Table S4, Fig. S29, S30). Enrichment of niches driving genes confirmed the significantly increased scores of COPD-endobronchial and control-endobronchial in the subsets of airways (Fig. S29). Similar to our results, airway ROIs with high COPD-endobronchial scores (NMF_7_ROIs) exhibited upregulation of mucosal response–associated genes, including *MUC5B* and *BPIFB1*, together with significantly higher proportions of goblet, ciliated, and cytotoxic T cells, and cDC1 (Fig. 5F–G). In contrast, ROIs enriched for the control-endobronchial signature (NMF_19_ROIs) showed elevated expression of progenitor-associated markers such as *SCGB3A2* and an increased abundance of club cells (Fig. 5F–G). Overall, these findings provide evidence for distinct airway remodelling-associated and homeostatic programs within the airway microenvironment. The relative proportion of airways assigned to NMF_7 or NMF_19 in the GeoMx datasets is shown in Fig. S29I. To investigate the clinical relevance of control-endobronchial and COPD-endobronchial airway niches, we examined the correlation between niche proportion and clinical information including airflow limitation (FEV1%, ratio of FEV1/FVC) and smoking history (pack-years). NMF_7 proportion was negatively correlated with the FEV1/FVC ratio (*R* = −0.68, *p* = 0.015), and we observed a trend towards a positive correlation between NMF_19 proportion and FEV1% (*R* = 0.57, *p* = 0.11), suggesting potential associations between airway niche composition and disease severity (Fig. S30A-C).

### Distinct transcriptional profiles and cellular landscapes in COPD airways

We next examined how disease status affects the gene profiles and consequently molecular and cellular composition in each airway niche. In the COPD-endobronchial niche, COPD airways exhibited a significant increase in goblet, cytotoxic T, regulatory T cells, and non-classical monocytes, along with a decreased proportion of club cells (Fig. S31). This niche was characterized by the highest number of deregulated genes (*n* = 61), including *BPIFA1* and *MUC5AC,* and enrichment of pathways related to humoral response, antigen presentation, and cytotoxicity (Fig. S32A-C) relative to controls. In contrast, control-endobronchial niche upregulated genes associated with epithelial repair (*ELF3*), xenobiotic metabolism (*NCOA7*), and pathways related to response to lipopolysaccharide and leukocyte migration (Fig. S32D-F).

Overall, these findings demonstrate extensive reprogramming in COPD airway compartment, mediated in part by altered cell composition and transcriptional changes of airway resident cells, typical of COPD: loss of club cells, increased mucin production and chronic inflammation infiltrate.

### Cytotoxic T cells were colocalised with goblet cells and linked to airway remodelling in COPD

**Fig. 6:**
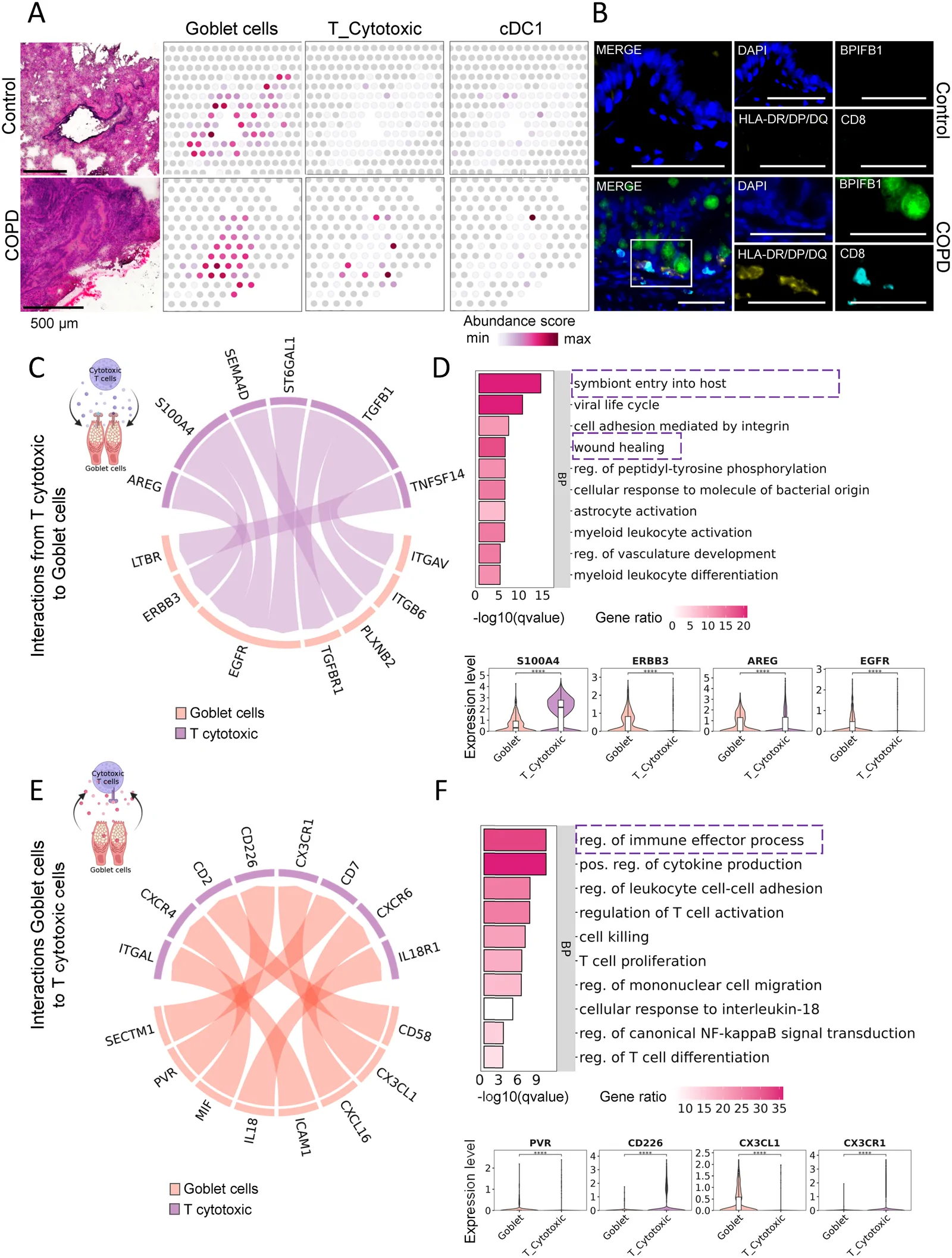
Colocalisation of cytotoxic T and goblet cells may contribute to airway remodelling in COPD (**A**) Zoomed H&E images of representative airways and corresponding spatial maps showing cell associated transcript abundance of goblet cells, cytotoxic T cells, and cDC1 within the COPD endobronchial niche (NMF_7) in control and COPD lungs. Colour indicates the deconvolution enrichment score of the niche. (**B**) Multiplex immunofluorescence staining of goblet cells (BPIFB1^+^, green), cytotoxic T cells (CD8^+^, turquoise), and antigen-presenting cells (HLA-DR/DP/DQ^+^, yellow) within airways of control and COPD lungs. Representative images from three control and three COPD samples are shown. Scale bar = 50 µm. (**C**) Ligand–receptor interactions inferred using the nichenetr R package on integrated control and COPD scRNA-seq datasets (GSE136831, GSE171541). Circos plot highlights prioritised ligands expressed by cytotoxic T cells and their corresponding receptors on goblet cells. Ligand–receptor gene pairs were filtered to retain spatially colocalised interactions involving secreted ligands within the COPD endobronchial niche. Functionally relevant, literature supported ligand-receptor gene pairs were visualised. Colours indicate cell types, and directed edges represent signalling from sender to receiver. (**D**) Bar plot showing significantly enriched pathways (qvalue ≤ 0.05) derived from receptors and predicted target genes identified in (**C**). Colour represents the ratio of expressed genes to total gene set size, and the x-axis indicates pathway significance. BP denotes gene ontology biological process. Violin plots show RNA expression of selected ligand or receptor genes across goblet and cytotoxic T cells in COPD samples. Statistical significance was assessed using Wilcoxon tests with FDR correction (* padj ≤ 0.05, ** padj ≤ 0.01, *** padj ≤ 0.001, **** padj ≤ 0.0001). (**E**) Reciprocal ligand–receptor interactions inferred using nichenetr, showing prioritised ligands expressed by goblet cells and corresponding receptors on cytotoxic T cells. Interactions were filtered for spatially co-expression within the COPD endobronchial niche and visualised as directed edges coloured by sender and receiver cell types. (**F**) Enriched pathways (qvalue ≤ 0.05) derived from receptors and target genes identified in (**E**), with accompanying violin plots showing expression of selected ligand or receptor genes across goblet and cytotoxic T cells in COPD

Given the transcriptionally inferred abundance of cytotoxic T cells, a similar pattern observed in the parenchymal remodelling-stress niche in the parenchyma, and their proximity with goblet and cDC1 within COPD-endobronchial niche (Figs. 5E, G, 6A, S33A-C), we validated their spatial localization using immunofluorescence staining. Compared to controls, COPD airways were characterized by abundant BPIFB1^+^ epithelial cells, cytotoxic T cells, HLA-DR^+^ antigen presenting cells (Fig. 6B, S33D).

To investigate potential cellular interactions between cytotoxic T cells and goblet cells within COPD-endobronchial niche, we performed a receptor ligand analysis on both COPD-endobronchial spots and the integrated scRNA-seq control and COPD dataset. Similar to above, only experimentally validated and spatially colocalised receptor-ligand pairs within NMF_7 (radius of 200 µm) were considered, and only functionally relevant, spatially validated, secreted interactions were visualised, as highlighted by *AREG-EGFR, S100A4–ERBB3, and ST6GAL1-EGFR* interactions (Fig. 6C, 34A). Pathways based on ligands from cytotoxic T cells with receptor and target genes in goblet cells (Fig. S34B) showed enrichment of not only entry into host process but also wound healing (Fig. 6D). Specifically, *EGFR* was identified as wound healing-associated genes, showing higher expression in goblet cells, relative to cytotoxic T cells (Fig. 6D), indicating the potential role of cytotoxic T cells in mediating airway remodelling process.

We further explored the signalling from goblet cells to cytotoxic T cells. Certain ligands such as, *CX3CL1* and *PVR*, were expressed by goblet cells and predicted to bind to *CX3CR1* and *CD226*, respectively (Fig. 6E). Similar to cytotoxic T – goblet cell cross talk, pathway analysis of ligand, receptor, and downstream target genes (Fig. S34C-D) revealed pathways related to immune effector and cytokine production. Goblet–cytotoxic T cell interactions were associated with T cell activation potentially driven by *CX3CR1* binding in cytotoxic T cells (Fig. 6F), suggesting potential paracrine signalling in supporting persistence of cytotoxic T cells by goblet cells in COPD airways. Furthermore, the predicted interactions in milder COPD lungs (GSE173896) validated the enriched receptor-secreted ligand interactions and involved pathways between cytotoxic T cells and goblet cells (Fig. S35).

## Discussion

Our study applies spatially resolved transcriptomics to profile controls and advanced COPD lungs with three major strengths: 1) the identification of specific compartmental niches that would have otherwise remained undetected in bulk analysis, 2) the unbiased discovery of potential crosstalk between cytotoxic T and goblet cells within the airway as well as with AT2 cells in the parenchyma, and 3) the global characterization of disease-associated transcriptional and cellular alterations across major lung compartments.

In the distal airways, we found that the healthy airway niche was driven by club cell-associated genes and antioxidative capacity while COPD-endobronchial niche shifted towards enriched goblet cells, decreased club cells, and a prominent association between cytotoxic T cells and goblet cells. The healthy niche was enriched with genes typical for secretory and progenitor cells [60–62], indicating the maintenance of progenitor capacity in healthy airways. In contrast, the loss of club cells and increased goblet cells in COPD-endobronchial niche, is consistent with previous report [62]. Similarly, the increased ciliated cells may represent a localised abundance of primary, non-motile cells within this niche, as described previously [63–65] suggesting compositional skew and aberrant differentiation associated with small airway remodelling in COPD.

Although immune alterations were observed across all compartments, cytotoxic T cells were prioritized for in-depth investigation because of their consistent involvement in both airway and parenchymal compartments. Both single-cell and in situ technologies have consistently highlighted the expansion and clinical relevance of cytotoxic T cells in COPD [8, 17, 60, 66]. Our spatial transcriptomics reveals how these cells could shape the local tissue environment and provides spatially resolved evidence that cytotoxic T cells actively influence distinct epithelial remodelling programs across specific airway and parenchymal niches. Within the airway, we identified several pathways linking cytotoxic T cells to goblet cell-enriched regions. Among these, EGFR signalling has been associated with mucus hypersecretion and goblet cell hyperplasia [67–69]. A defining feature of the COPD endobronchial niche was the marked enrichment of mucin-interacting proteins, including *SLPI*, Polymeric immunoglobulin receptor (*PIGR*), Tetraspanin 1 (*TSPAN1*), and *BPIFB1*, a mucosal-defence protein increasingly linked to epithelial remodelling in chronic airway disease. *BPIFB1* expression rises across GOLD 2–4 COPD and correlates with reduced lung function and goblet cell expansion [70, 71]. As BPIFB1 is produced by goblet cells [72] and regulates *MUC5AC* and *MUC5B* expression [73], its upregulation may not simply reflect epithelial plasticity, but may also contribute to the persistence of mucus hypersecretion. Whether these intensified interactions between cytotoxic T cells and mucus-producing cells also contribute to mucus plugging, an increasingly recognised driver of COPD pathology [74], remains an important open question. Interestingly, increased spatial interactions between cytotoxic T cells and mucus-producing cells have also been observed in GOLD 2 lungs [66], raising the question whether similar pathways are already at work in milder COPD stages.

Beyond promoting goblet-cell–associated remodelling, cytotoxic T cells may simultaneously impair airway repair by inducing epithelial senescence and reducing club cell abundance through IFN-γ–mediated mechanisms [75]. Our data indicates that the sustained accumulation of immune cells within the COPD endobronchial niche may partly stem from epithelial–immune crosstalk. Loss of club cells, and their secretory product CC16/ SCGB1A1, has been linked to heightened airway inflammation [76], suggesting a cyclical process in which cytotoxic T cell activity exacerbates both epithelial dysfunction and inflammatory tone. Epithelial cells can deliver antigens to DC [77], which, together with antigen presentation by both epithelial cells and dendritic cells, may help to maintain ongoing cytotoxic T cell activation locally. cDC2 are able to promote the differentiation of follicular helper T (Tfh)-like cells from naïve CD4^+^ T cells, implicating a role in tertiary lymphoid organ formation [78]. These structures were prominent especially in GOLD 3–4 parenchyma and peribronchial areas [79, 80], highlighting a potential role of cDC2 in driving lymphocytic infiltration and aggregate formation in COPD airways.

Several of the genes associated with the COPD endobronchial niche may represent possible biomarkers. Increased plasma protein levels of Human Epididymal Protein 4 (HE4/WFDC2), PIGR, Peroxiredoxin-5 (PRDX5), and BPIFB1, correlate inversely with lung function [81–84]. Similarly, elevated sputum levels of SLPI and BPIFB1 are associated with increased disease severity and reduced FEV1% [70, 85–87], while reduced levels of CC16 in serum and sputum have consistently been linked to increased COPD severity and emphysema progression [88–90].

In the parenchyma, the transcriptional landscape showed greater spatial complexity and pronounced differences between COPD and control tissues. Two distinct immune-enriched niches were identified-one enriched humoral immune signatures and another dominated by macrophage gene signatures—that likely correspond to previously described lymphoid follicles [22, 91] and macrophage niche/aggregates [62, 92, 93], respectively. Macrophages within the macrophage-enriched niche displayed activation profiles consistent with increased reactive oxygen species generation, a mechanism known to amplify inflammatory pathways and promote emphysematous tissue injury [94]. Consistent with this, we previously showed that increasing numbers of activated macrophages negatively correlate with oxygenation levels in severe COPD [17]. Together, these data support the concept that the COPD parenchyma contains distinct immune microenvironments that may sustain chronic inflammation and contribute to structural degradation.

The identification of a COPD-specific remodelling–stress areas in the parenchyma, was marked by heightened AP-1 gene activity together with AT2 cells, cytotoxic T cells, and NK cells. AP-1 components, such as FOS and JUN, have previously been noted in COPD lungs and are characteristic of specialised AT2 subsets, including aged inflammatory AT2 cells that secrete chemokines such as CCL2, CXCL1, CXCL2, and CXCL8 [18, 95]. These same chemokines were upregulated in this remodelling–stress region, indicating that AT2 cells here may act as active organisers of immune recruitment rather than passive responders to damage. Consistent with this, AT2-associated transcript may be preferably enriched due to loss of multi-cellular signatures consistent with enriched pathways associated with stress, hypoxia response, and cell killing. The transcriptional profile further recapitulated features of AT2B cells [20], including enhanced stress-response signalling and altered EGR1 activity previously linked to COPD-related epithelial dysregulation. Collectively, these observations point to a stressed, pro-inflammatory AT2 microenvironment that may amplify local immune activation and drive aberrant tissue remodelling in the COPD parenchyma.

AP-1 signalling may influence AT2 cell susceptibility to cytotoxic T cells-mediated pathways. Experimental studies show that c-JUN can upregulate *FAS* transcription, increasing FAS surface expression in epithelial cells [96], The predicted transcriptional regulation of *FAS* by *FOS* and *JUN* in our dataset indicates that AP-1 activity could enhance FAS-dependent signalling in AT2 cells. Given that cytotoxic T cells express *FASL*, this regulation may promote *FASL*–*FAS* interactions within the parenchyma.

In addition, IFN-γ, a key cytokine produced by cytotoxic T cells, can interact with *IFNGR1* receptor on AT2 cells and has been shown to preferentially eliminate specific AT2 subsets [97]. Furthermore, terminally differentiated cytotoxic T cells were predicted to interact with AT2 cells through IFNG signalling [15] and increased IFN-γ production is associated with epithelial injury [98]. Moreover, CD8^+^ cell cytolytic activity is enhanced by IL-18 [99], which in our data may derive from AT2 cells themselves. These observations together support a model in which epithelial stress pathways and cytotoxic T cell-associated signalling are closely interconnected within COPD parenchyma.

Finally, it is tempting to speculate that the COPD-endobronchial and parenchymal remodelling–stress niches identified here may be differentially abundant across known COPD clinical phenotypes (e.g., emphysema-predominant vs. airway-predominant). However, validation in larger and clinically well-characterised cohorts will be required to test this hypothesis.

### Limitations of the study

Several limitations should be considered when interpreting our findings. Firstly, COPD is a spatially and temporally heterogenous disease. While our data from patients with advanced disease provide relevant cross-sectional spatial insights into this disease stage, they may not fully capture the spectrum of COPD subtypes. Although similar processes may have a role in earlier COPD stages, our study design does not allow conclusions regarding causality or dynamics of disease progression. Despite the unbiased design of this study, spatial transcriptomics was necessarily limited to selected tissue regions and may therefore not capture the full spectrum of COPD pathology. Frozen sections were chosen to include all three major lung compartments, enabling compartment-specific analyses. In addition, technical constraints of the Visium protocol required thick (10 µm) fresh-frozen sections. Although this preserved RNA integrity, it can compromise tissue morphology. Future studies with broader representation of COPD pathology and detailed histopathological annotation will be important.

Secondly, our spatial method captures a mini bulk of whole transcriptome profiles, requiring deconvolution to estimate cellular composition within each spot. As deconvolution algorithm can be unstable and potentially generates noisy outputs, we employed a reference matrix derived from relevant control and COPD scRNA-seq datasets [19, 20], applied stringent filtering to retain robust estimates, and prioritized intra-cell type comparisons across disease conditions to minimize estimation bias. Key results were validated by immunofluorescence and flow cytometry. In addition, limited sequencing depth may have reduced detection sensitivity for rare cell populations, while large structural cells, such as airway epithelial and smooth muscle cells, were more likely to dominate transcript capture within individual spots. Accordingly, our conclusions were restricted to the most robustly detected populations, and future studies specifically designed to resolve rare cell types will be important.

Thirdly, niche discovery was performed in a relatively small cohort and, given the unbiased analytical framework, our findings should be considered hypothesis-generating rather than hypothesis-testing. To strengthen robustness, we incorporated an independent and larger spatial transcriptomic cohort [22], which validated the airway-associated transcriptional signatures in greater depth. In contrast, validation within the parenchyma was more limited, likely reflecting greater spatial heterogeneity and differences in spatial resolution between datasets, with the higher spot number in Visium providing increased statistical power and sensitivity for DEG detection [100]. Although we identified potential links between cytotoxic T cells and both AT2 and goblet cells, suggesting roles in tissue remodelling, the associations observed in this cross-sectional study do not establish causality and may reflect confounding or reverse causation. While the *IFNG*–*IFNGR1* interaction was validated using an in vitro co-culture system, additional longitudinal, in vitro, and in vivo studies will be required to validate other predicted interactions and define their mechanistic significance.

Fourthly, information on ancestry, race, and ethnicity was not collected for spatial, immunofluorescence and flow cytometry cohorts, limiting assessment of population diversity and generalizability. However, the study cohort is likely enriched for individuals of White Central European ancestry as stated previously [17, 32]. Sex- and gender-related effects was not systematically evaluated due to limited control sample availability, and future studies in larger and more diverse cohorts will be required to address this. In addition, differences in smoking status and age within the spatial cohort may represent potential confounders. To mitigate this, the presence of airway niches was validated in an independent spatial dataset with comparable smoking-pack years. Nevertheless, our spatial cohort consists of donors who were never-smokers and COPD patients who were ex-smokers and had ceased smoking for at least two years prior to sample collection, consistent with lung transplantation eligibility criteria. Consequently, the transcriptional differences identified in this study cannot be unequivocally attributed to COPD pathology alone, as persistent effects of prior smoking may also contribute. Therefore, these findings as reflecting should be interpreted as a combination of COPD-associated and long-term smoking-related changes, rather than the acute effects of ongoing smoke exposure.

Furthermore, key findings on immune abundance and spatial localisation were validated using flow cytometry and immunofluorescence in independent cohorts with more comparable group age distributions. Receptor–ligand analyses were likewise performed in a larger scRNA-seq dataset with similar age characteristics, and key interactions were further supported in an independent GOLD 1–2 dataset. While these data suggest that some interactions may already be present in milder disease, we note that this cohort may be confounded by co-existing emphysema and underlying lung cancer in COPD patients; therefore, these findings should be interpreted with caution.

## Conclusions

Collectively, we provide a spatially resolved characterization of disease-specific cellular niches across the airway, vasculature, and parenchyma in advanced COPD, identifying compartment-specific immune infiltration and transcriptionally distinct microenvironments that contribute to sustained inflammation and tissue remodelling. We show that cytotoxic T cells are enriched within defined airway and parenchymal niches, where they are predicted to engage in distinct interactions with goblet cells and AT2 cells, respectively, potentially driving epithelial differentiation programmes and cytotoxic stress responses. These insights highlight that spatial relationships are central to the biological interpretation and propose immune–epithelial interactions as actionable targets for context-specific therapeutic intervention in COPD.

## Electronic supplementary material

Below is the link to the electronic supplementary material.


Supplementary Material 1



Supplementary Material 2



Supplementary Material 3


## Data Availability

Data: The datasets used and/or analysed during the current study are available with the following details. Raw data of spatial transcriptomics was deposited into the Gene Expression Omnibus database under accession number GSE318309, currently under embargo until January 04^th^, 2027, and can be securely access at the following URL: https://identifiers.org/geo:GSE318309 with token code ibijegmsprqjvub. Raw data of multiplex immunofluorescence imaging and flow cytometry data are available at Mendeley Data: https://doi.org/10.17632/8nm3w6xvr8.3. The raw data for scRNA-seq are available under GEO: GSE136831 and GSE171541. Raw data of GeoMx spatial transcriptomics is available under GEO: GSE292993.

## Abbreviations

AT: Alveolar epithelial cell Type
BPIFB1: BPI Fold Containing Family B Member 1
CCL: C-C motif chemokine ligand
COPD: Chronic obstructive pulmonary disease
CXCL: C-X-C motif chemokine ligand
DC: Dendritic cell
DEG: Differentially expressed genes
DLCOcSB: Single breath diffusing capacity of the lung for carbon monoxide corrected for haemoglobin
ECM: Extracellular matrix
FAS: Fas Cell Surface Death Receptor
FDR: False Discovery Rate
FEV1: Forced expiratory volume in 1 second
FVC: Dorced vital capacity
H&E: Haematoxylin and eosin
IFNG/IFN-γ: Interferon Gamma
mPAP: Mean pulmonary arterial pressure
MUC: Mucin
NMF: Non-negative matrix factorization
PCA: Principal component analysis
pO2: Capillary partial pressure of oxygen
ROI: Region on interest
scRNA-seq: Single-cell RNA sequencing
SMC: Smooth Muscle Cells
ST: Spatial Transcriptomics
TLC: Total lung capacity
UMAP: Uniform manifold approximation and projection

## Code availability

The R scripts required to recreate the figures in this article are available at: https://github.com/Lung-research-group/Spatial_Transcriptomics_COPD.
