# Supplementary material for: Spatial transcriptomics reveals compartment-specific cytotoxic T cell crosstalk with AT2 and goblet cells in advanced COPD
Source: Biomark Res. 2026 Jun 25;14:101. doi: 10.1186/s40364-026-00960-w (PMC13536628; doi:10.1186/s40364-026-00960-w)
Supplement: Supplementary file 1 [file 40364_2026_960_MOESM1_ESM.pdf]

Supplementary Information

Table S1. Antibodies used for multiplex immunofluorescence staining

Target	Dilution	Company	REF ID	pH	Panel	Label	Sequence
CD19	1:200	Biorad	AB_566586	pH6	Overview	Opal 570	1
CD68	1:100	Santa Cruz	AB_627158	pH9	Overview	Opal 690	2
MPO	1:1000	Abcam	AB_2864724	pH6	Overview	Opal 520	3
CD3	1:100	Dako	AB_2335677	pH9	Overview	Opal 780	4
NKp46	1:100	Thermo Fisher	AB_2852244	pH9	Parenchyma	Opal 690	1
Pro-SPC	1:3000	Milipore Merck	AB_91588	pH9	Parenchyma	Opal 570	2
CD8	1:100	Sigma Aldrich	AB_2675648	pH6	Parenchyma	Opal 520	3
c-FOS	1:100	Santa Cruz	AB_2231996	pH9	Parenchyma	Opal 780	4
HLA-DR	1:100	Abcam	AB_306142	pH9	Airway	Opal 570	1
LPI-A (BPIFB1)	1:100	-	-	pH6	Airway	Opal 520	2
CD8	1:100	Sigma Aldrich	AB_2675648	pH6	Airway	Opal 780	3

Table S2. Antibodies used for flow cytometry, in vitro coculture, and western blot

Panel	Antigen	Label	Dilution	Company	REF ID	Clone
**1**	CD3	FITC	1:20	Immunotools	N/A	MEM-57
	CD56	ef450	1:10	Thermo Fisher	AB_2574021	TULY56
	CD16	PE-Cy7	1:20	BD Biosciences	AB_396850	3G8
	CD19	APC	1:20	BioLegend	AB_314242	HIBI9
	CD45	AF700	1:200	BioLegend	AB_493761	HI30
	HLA-DR	APC-Cy7	1:100	Thermo Fisher	AB_1582284	LN3
	CD14	BV786	1:20	BD Biosciences	AB_2744287	M5E2
**2**	CD3	FITC	1:20	Immunotools	N/A	MEM-57
	gdTCR	PE	1:20	Thermo Fisher	AB_1603300	B1.1
	CD45	PerCp-Cy5.5	1:100	BD Biosciences	AB_2744405	HI30
	CD4	APC-Cy7	1:50	BioLegend	AB_2028481	SK3
	CD8	SB600	1:20	Thermo Fisher	AB_2637470	RPA-T8
**3**	CD169	PE	1:10	Thermo Fisher	AB_10598796	7-239
	CD206	PerCp-Cy5.5	1:100	BioLegend	AB_10899411	15-2
	CD45	APC	1:100	BioLegend	AB_314400	HI30
	HLA-DR	APC-Cy7	1:100	Thermo Fisher	AB_1963603	LN3
**4**	FcER1	FITC	1:20	Thermo Fisher	AB_10732835	AER-37
	CD123	PE-Cy7	1:20	Thermo Fisher	AB_1257136	6H6
	CD117	APC	1:10	Thermo Fisher	AB_10596820	YB5.B8
	CD16	AF700	1:100	BioLegend	AB_2278418	3G8
	CD3	ef450	1:20	Thermo Fisher	AB_1518799	UCHT1
	CD14	SB600/BV605	1:10	BD Biosciences	AB_2687593	M5E2
	HLA-DR	APC-Cy7	1:100	Thermo Fisher	AB_10718248	LN3
**5**	IFN-γ	AF488	1:20	BioLegend	AB_493029	4S.B3
**6**	pSTAT1	PE	1:20	BioLegend	AB_2734526	A17012A
	CD45	PerCp-Cy5.5	1:100	BD Biosciences	AB_2744405	HI30
	IFN-γ	N/A	10 µg/mL	BioLegend	AB_315435	B27
	IgG1 control	N/A	10 µg/mL	Abcam	AB_2722537	MGI-45
**7**	pSTAT1	N/A	1:1000	Cell Signalling	AB_561284	58D6
	STAT1	N/A	1: 1000	Cell Signalling	AB_2198300	N/A
	GAPDH	N/A	1:5000	Cloud-Clone Corp.	N/A	N/A

Table S3. Cell populations and respective markers as identified by flow cytometry.

Lineage		Cell type	Label	FSC	SSC	Surface Markers					
Lymphoid		T helper cells	T_Helper	Low	Low	$CD45^+$	$CD3^+$	$CD4^+$	$CD8^-$		
		Cytotoxic T cells	T_Cytotoxic	Low	Low	$CD45^+$	$CD3^+$	$CD4^-$	$CD8^+$	γδTCR-	
		γδ T cells	gDT	Low	Low	$CD45^+$	$CD3^+$	$CD4^-$	$CD8^-$	$γδTCR^+$	
		NKT cells	NKT	Low	Low	$CD45^+$	$CD3^+$				$CD56^+$
		NK cells	NK	Low	Low	$CD45^+$	$CD3^-$				$CD56^+$
		B cells	B	Low	Low	$CD45^+$	$CD3^-$			$CD19^+$	
Myeloid	Monocytes	Classical monocytes	cMonocyte	Med	Med	$CD45^{high}$		$CD14^{hi}$	$CD16^{neg}$		
		Intermediate monocytes	IntMonocyte	Med	Med	$CD45^{high}$		$CD14^{hi}$	CD16med		
		Non-classical monocytes	ncMonocyte	Med	Med	$CD45^{high}$		$CD14^{med}$	$CD16^{hi}$		
	Macrophages	Interstitial Macrophage	Macrophage	High	High	$CD45^{high}$	$HLA\text{-}DR^+$		CD206-	CD169-	
		Alveolar Macrophage	Mac_Alveolar	High	High	$CD45^{high}$	$HLA\text{-}DR^+$		CD206+		
	Dendritic cells (DC)	Plasmacytoid dendritic cells	pDC	Low	Low	$CD45^{high}$	$HLA\text{-}DR^+$	$CD14^-$	CD3-	$CD123^+$	FcER1-
	Granulocytes	Basophils	Basophil	Low	Low	$CD45^+$	$HLA\text{-}DR^-$	CD14-	CD3-	$CD123^+$	FcER1+
		Neutrophils	neutrophils	Med	Med	$CD45^+$	$HLA\text{-}DR^-$	$CD14^-$	$CD16^+$		
		Mast cells	Mast	Med	Med	$CD45^+$	CD16-	CD14-	CD3-	$CD193^-$	$CD117^+$

FSC: Forward Scatter; SSC: Side Scatter

Table S4. Patient characteristics of the GeoMx Nanostring spatial transcriptomics cohort

**Parameters**	**Control (n=16)**	**COPD (n=10)**
**Age**	69.7 [23-84] (16)	66.4 [55-77] (10)
**Sex (F/M)**	6 (38)/10 (62)	2 (20)/8 (80)
**Pack years**	33.8 [9-80] (9)	41.2 [15-100] (10)
**Quit years**	N/A	N/A
**FEV1, %predicted**	89.5 [65-122] (14)	31.6 [19-46] (10)
**FEV1/FVC, %predicted**	68.3 [61-92] (14)	25.6 [19-47] (7)
**Whole lung %LAA950**	1.1 [0-3.4] (15)	23.0 [5-47] (10)

Data are presented as n (%), mean [range with n indicating the number of individuals from whom data were obtained. Abbreviations: F/M, female/male; pack years, packs of cigarettes smoked per year; N/A, data not available; quit years, number of years since smoking cessation; FEV1, forced expiratory volume in 1 second; %predicted, percent predicted; FVC, forced vital capacity;
