# Supplementary material for: Spatial transcriptomics reveals compartment-specific cytotoxic T cell crosstalk with AT2 and goblet cells in advanced COPD
Source: Biomark Res. 2026 Jun 25;14:101. doi: 10.1186/s40364-026-00960-w (PMC13536628; doi:10.1186/s40364-026-00960-w)
Supplement: Supplementary file 2 [file 40364_2026_960_MOESM2_ESM.pdf]

Supplementary Figure S1

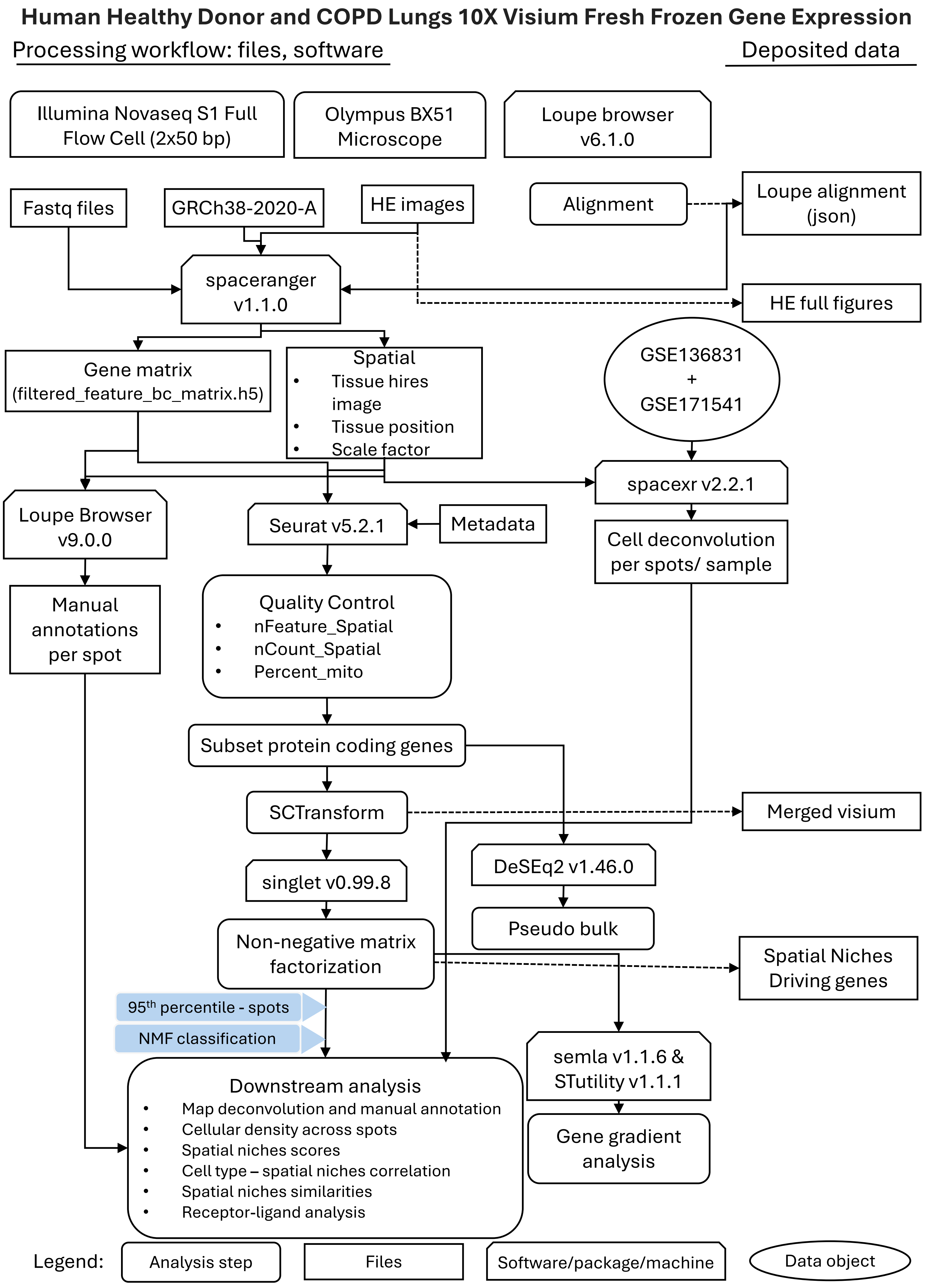

Figure S1: Workflow of Visium analysis

Supplementary Figure S2

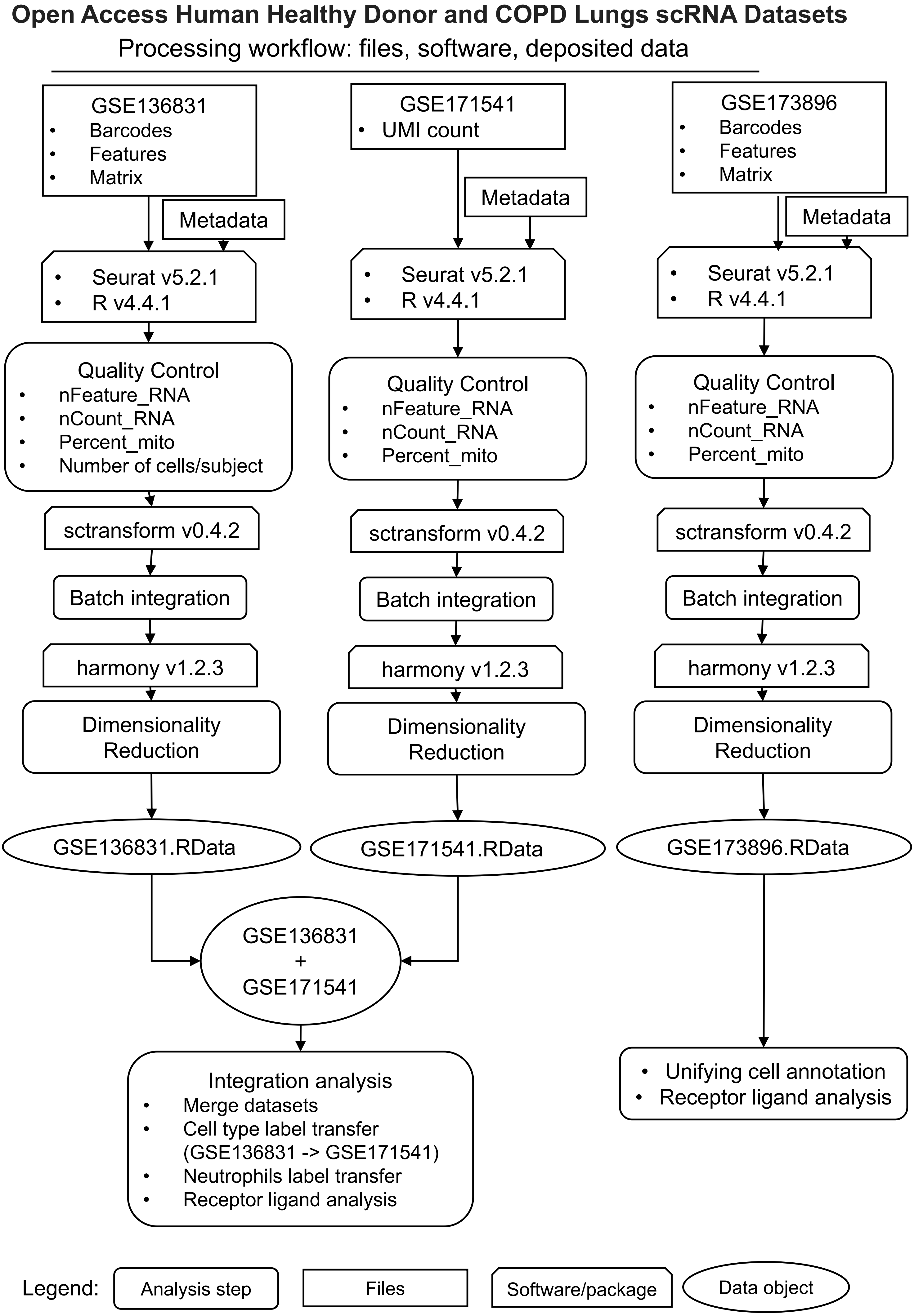

Figure S2: Workflow of integrated scRNA-seq analysis

Supplementary Figure S3

Open Access Human Non-disease Control and COPD Lungs GeoMx Nanostring Spatial Transcriptomic Dataset

Processing workflow: files, software, deposited data

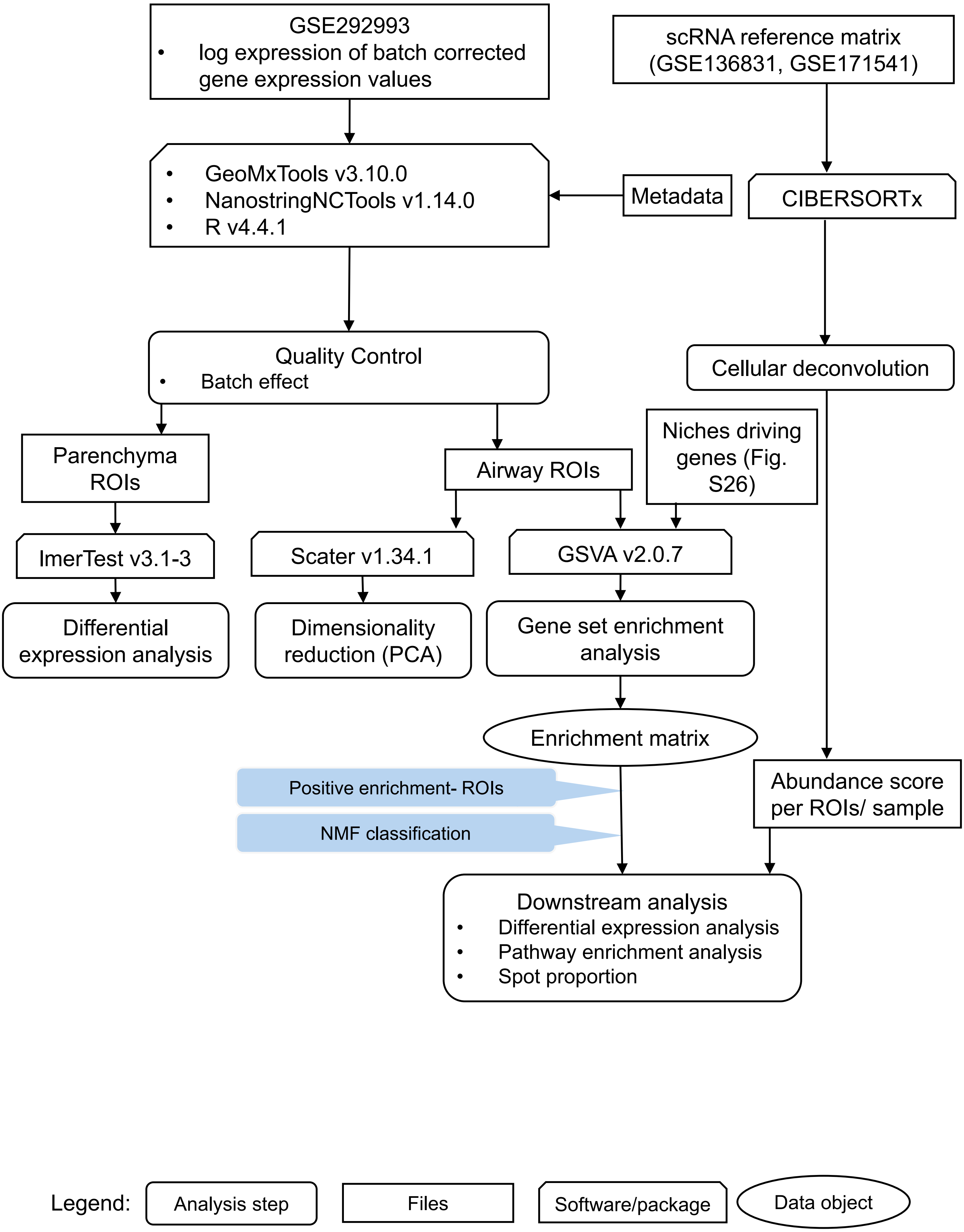

Figure S3: Workflow of GeoMx Nanostring Spatial Transcriptomic analysis

Supplementary Figure S4

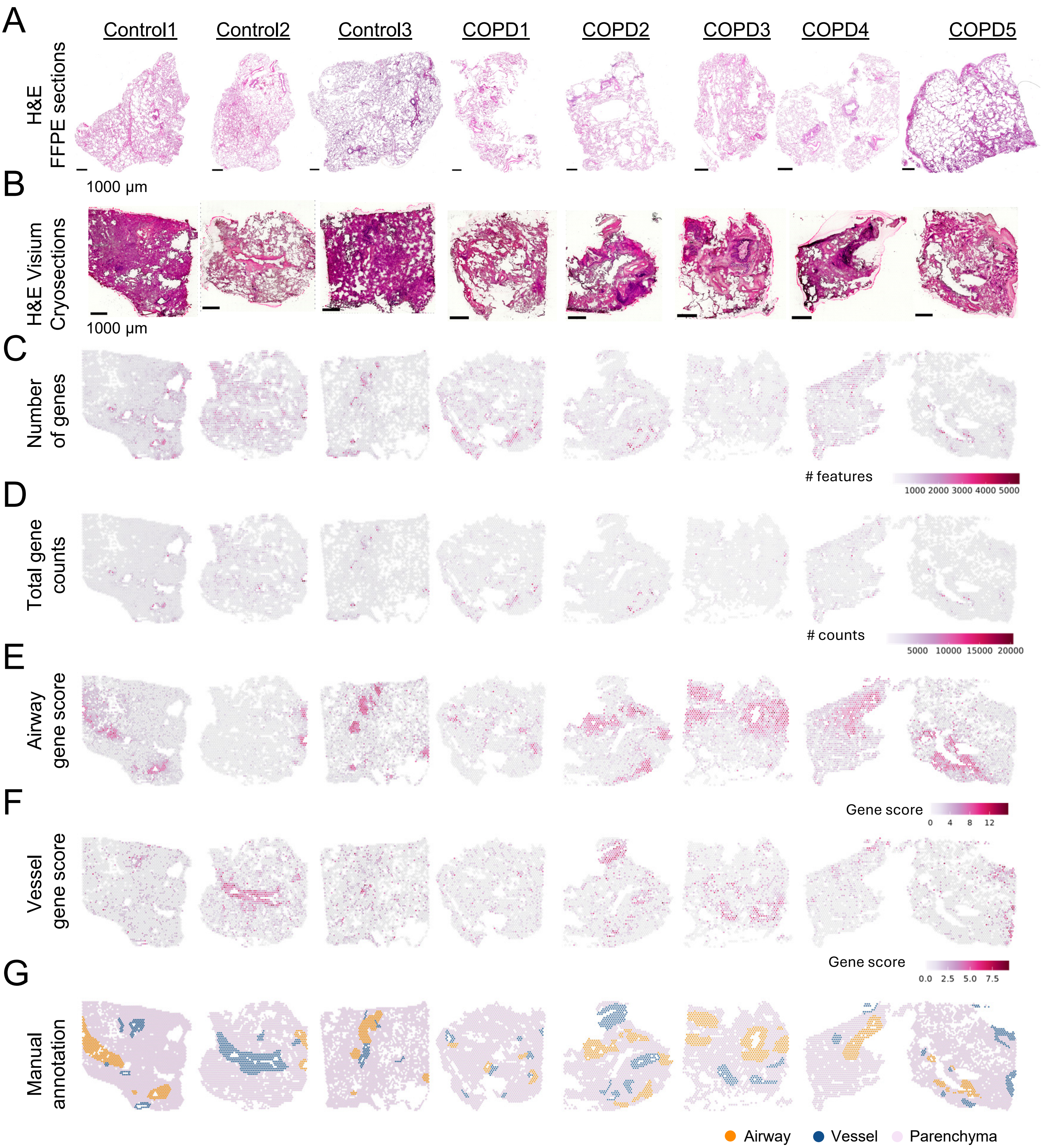

Figure S4: Spatial map across control and COPD samples

(A) Representative H&E images of FFPE lung sections obtained from the same lobe used for Visium spatial transcriptomics analysis of control and COPD samples. (B) H&E images of Visium spatial transcriptomics control and COPD samples. (C) Number of expressed genes detected per spot across control and COPD samples. (D) Library size, shown as total gene counts per spot, across control and COPD samples. (E) Spatial distribution of the airway gene signature, calculated as the average expression of KRT17, CAPS, SCGB3A2, and BPIFB1, and (F) vessel gene signature, calculated as the average expression of ACTA2, SFRP2, and ENPP2, across control and COPD samples. (G) Manual tissue annotation based on combined macroscopic assessment of lung compartments and compartment-specific gene signature scores.

Supplementary Figure S5

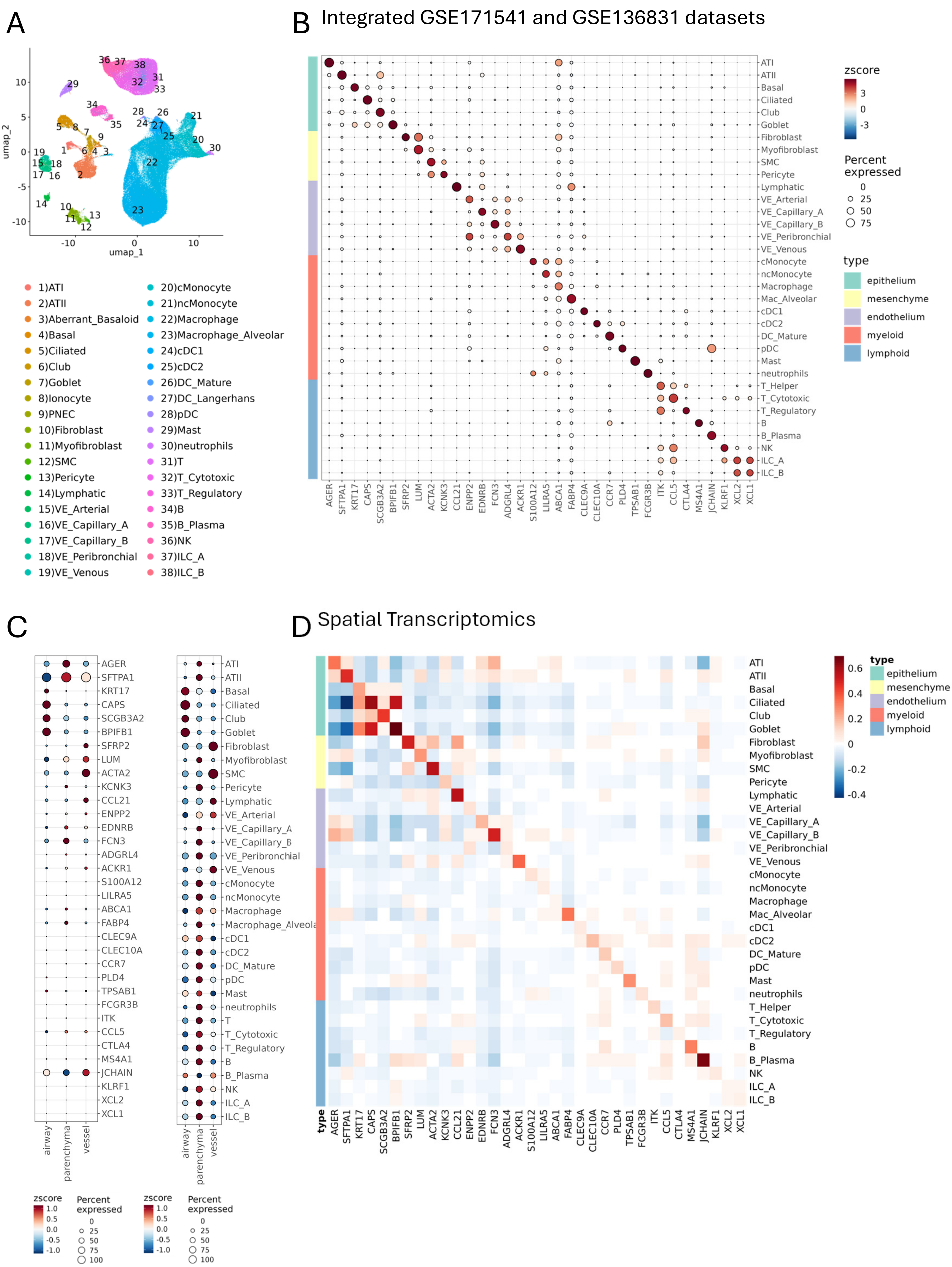

Figure S5: Cell type gene markers and spatial deconvolution

(A) UMAP showing cell type annotated in integrated scRNA (GSE171541, GSE136831) datasets. (B) Dot plots of gene marker expression across immune and structural cell types in scRNA dataset. Colour represents the expression level and size corresponds to the percentage of cells expressing the gene. Cell types are annotated with bar plot based on their broad category. (C) Dot plots of gene marker expression (left panel) and estimated cellular proportion (right panel) within manually annotated compartments in spatial transcriptomics dataset. Colour represents the expression level and size corresponds to the percentage of cells expressing the gene. (D) Correlation heatmap of gene marker expression and deconvoluted cell types. Colour indicates the strength and directionality of the correlation, calculated with pearson method. Rows represent cell types and columns represents genes.

Supplementary Figure S6

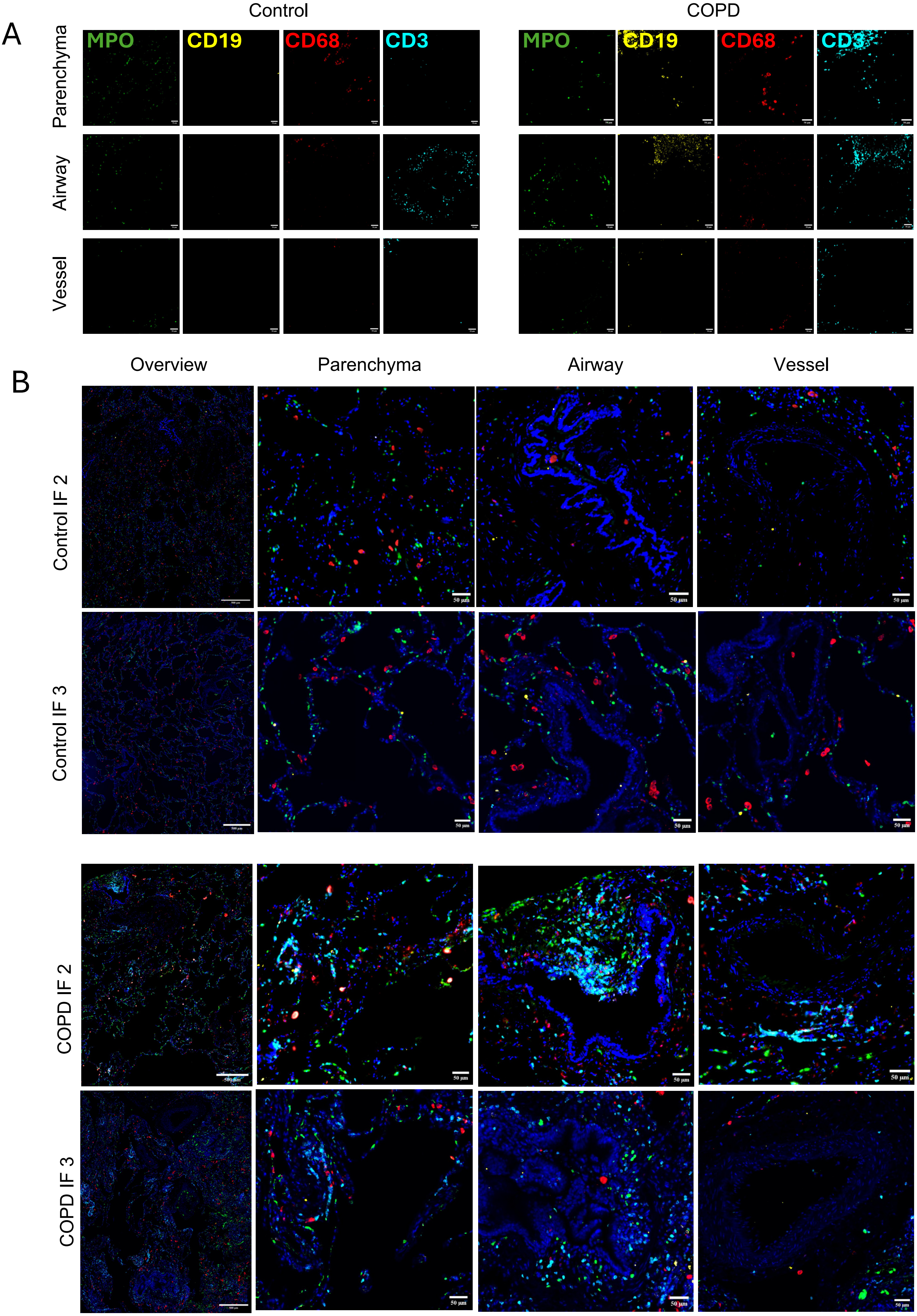

Figure S6: Validation of immune cell signatures of lung compartments with immunofluorescence

(A) Single colour stainings of neutrophils (MPO^{+}, green), B cells ($CD19^{+}$, yellow), T cells ($CD3^{+}$, turquoise), macrophages ($CD68^{+}$, red) on control and COPD lungs shown in Figure 1G. Scale bar is 50 µm. (B) Multiplex immunofluorescence stainings of neutrophils (MPO^{+}, green), B cells ($CD19^{+}$, yellow), T cells ($CD3^{+}$, turquoise), macrophages ($CD68^{+}$, red) on two control and two COPD lungs across three compartments; Scale bar is 50 µm.

Supplementary Figure S7

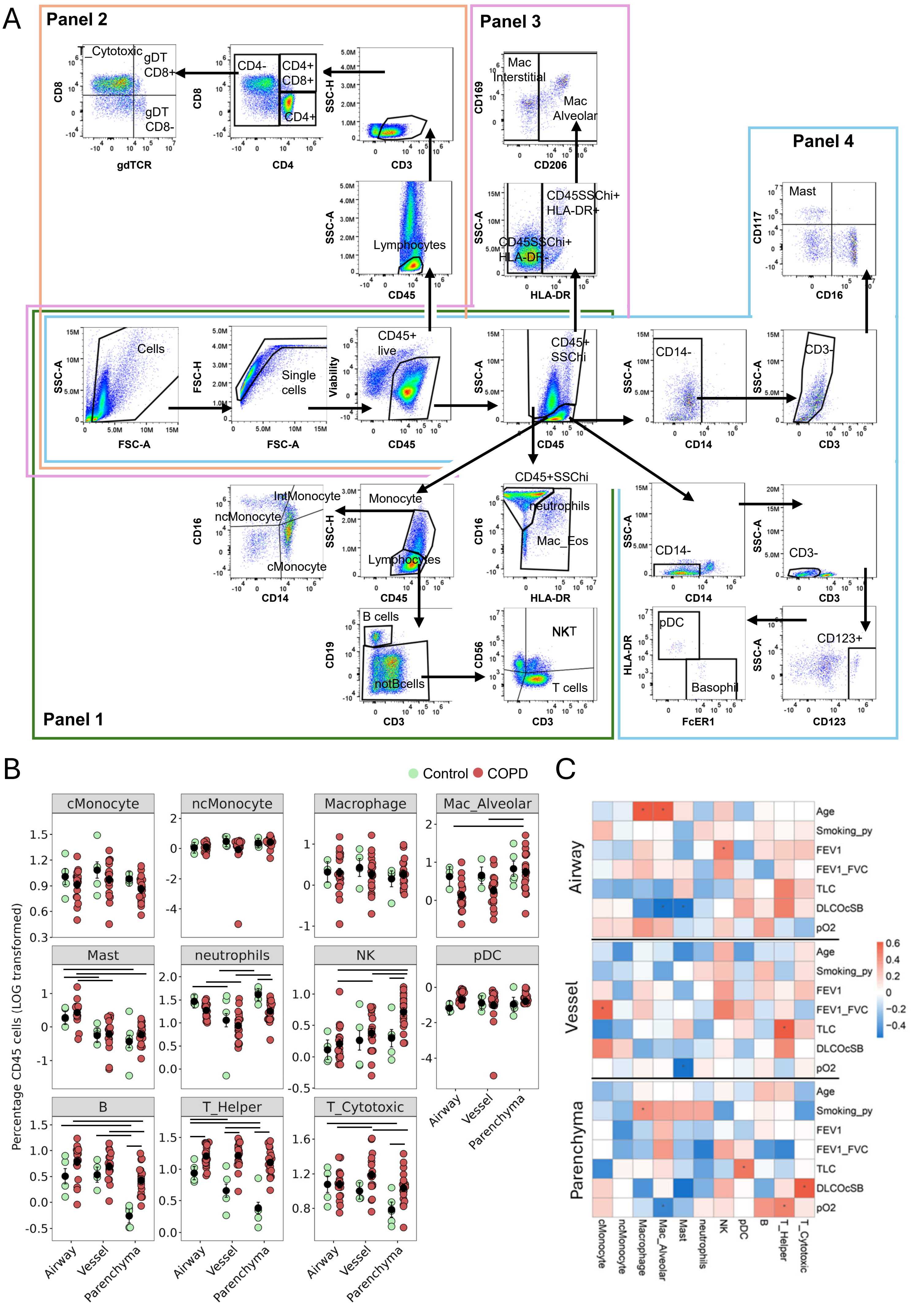

Figure S7: Validation of immune cell signatures of lung compartments with flow cytometry

(A) Flow cytometry gating strategy applied to identify immune cell populations from freshly isolated lung compartments of control and COPD. (B) Comparisons of log-transformed relative immune cell proportion, derived from percentage of $CD45^+$ populations, between COPD and controls across different lung compartments. Significance were calculated using linear mixed model adjusting for individual lungs as random effect, compartment and diagnosis as fixed effects; lines indicate $p_{adj} \leq 0.1$. Black dots and error bars represent estimated means ± standard errors, respectively. (C) Correlation between immune cells in COPD airways, (upper panel) vessels (middle), parenchyma (lower panel) and age, smoking pack-years (smoking_py), forced expiratory volume in 1s (FEV1), ratio of FEV1 and forced vital capacity (FEV1_FVC), capillary partial pressure of oxygen (pO2), total lung capacity (TLC), and single breath diffusing capacity of the lung for carbon monoxide corrected for hemoglobin (DLCOcSB). Correlation coefficient was calculated with spearman method (*$p \leq 0.05$).

Supplementary Figure S8

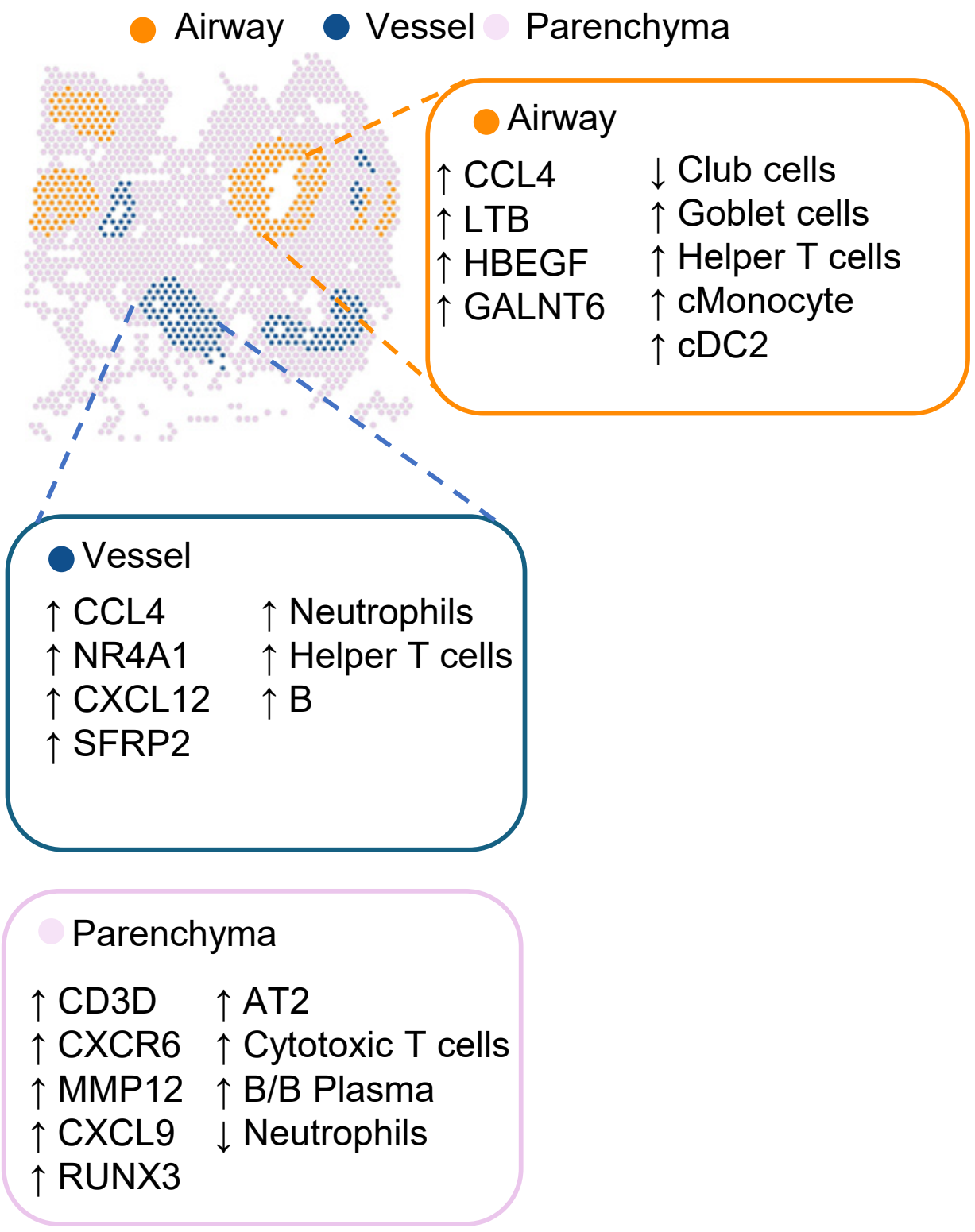

Figure S8: Schematic summary of differentially expressed genes and differentially abundant cell types across lung compartments.

Supplementary Figure S9

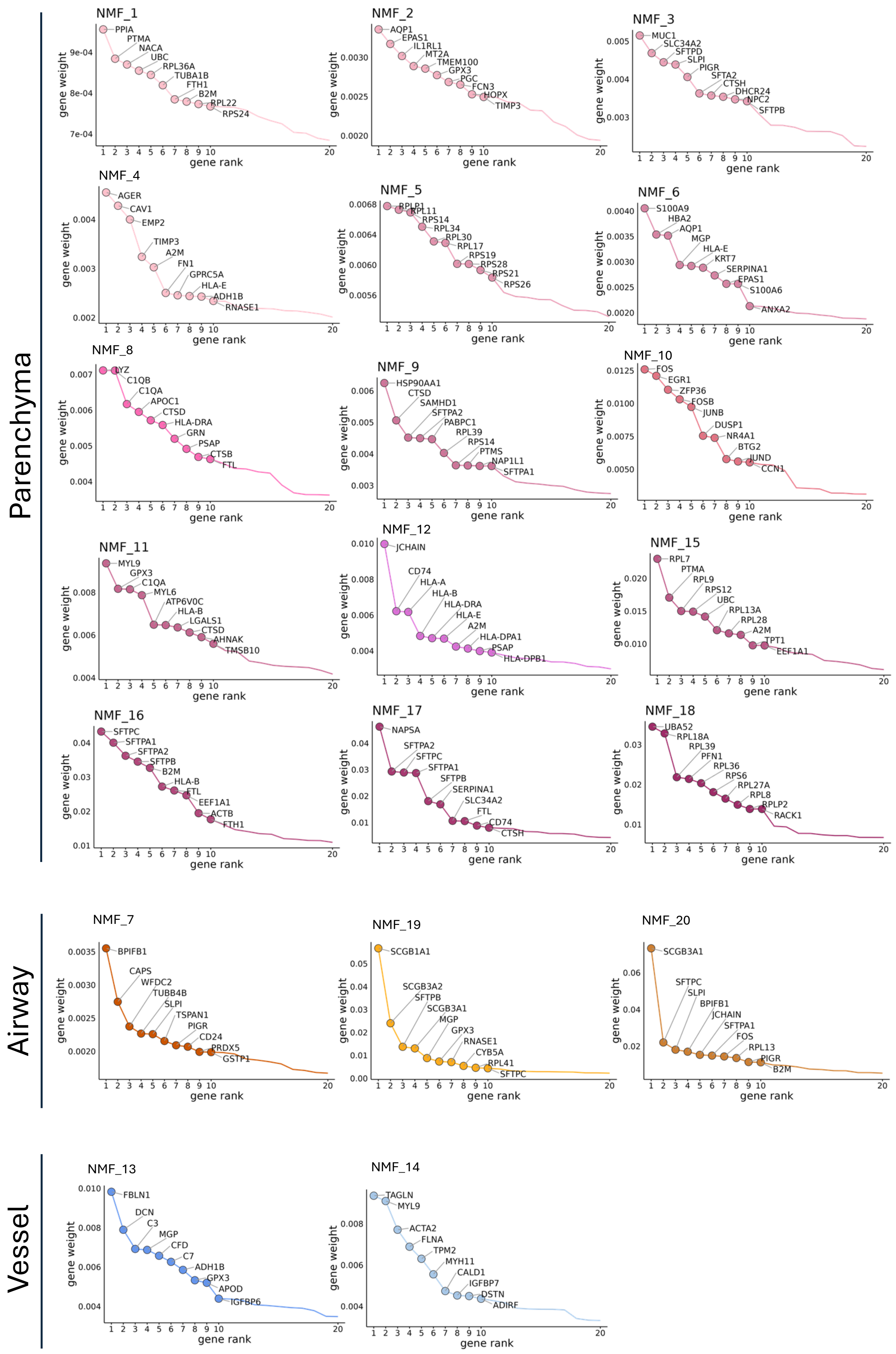

Figure S9: Driving genes of spatial niches

Top 10 driving genes of the 20 spatial niches corresponding to the lung compartments. Y axis represent the gene score, and x axis represents gene ranks within each niche.

Supplementary Figure S10

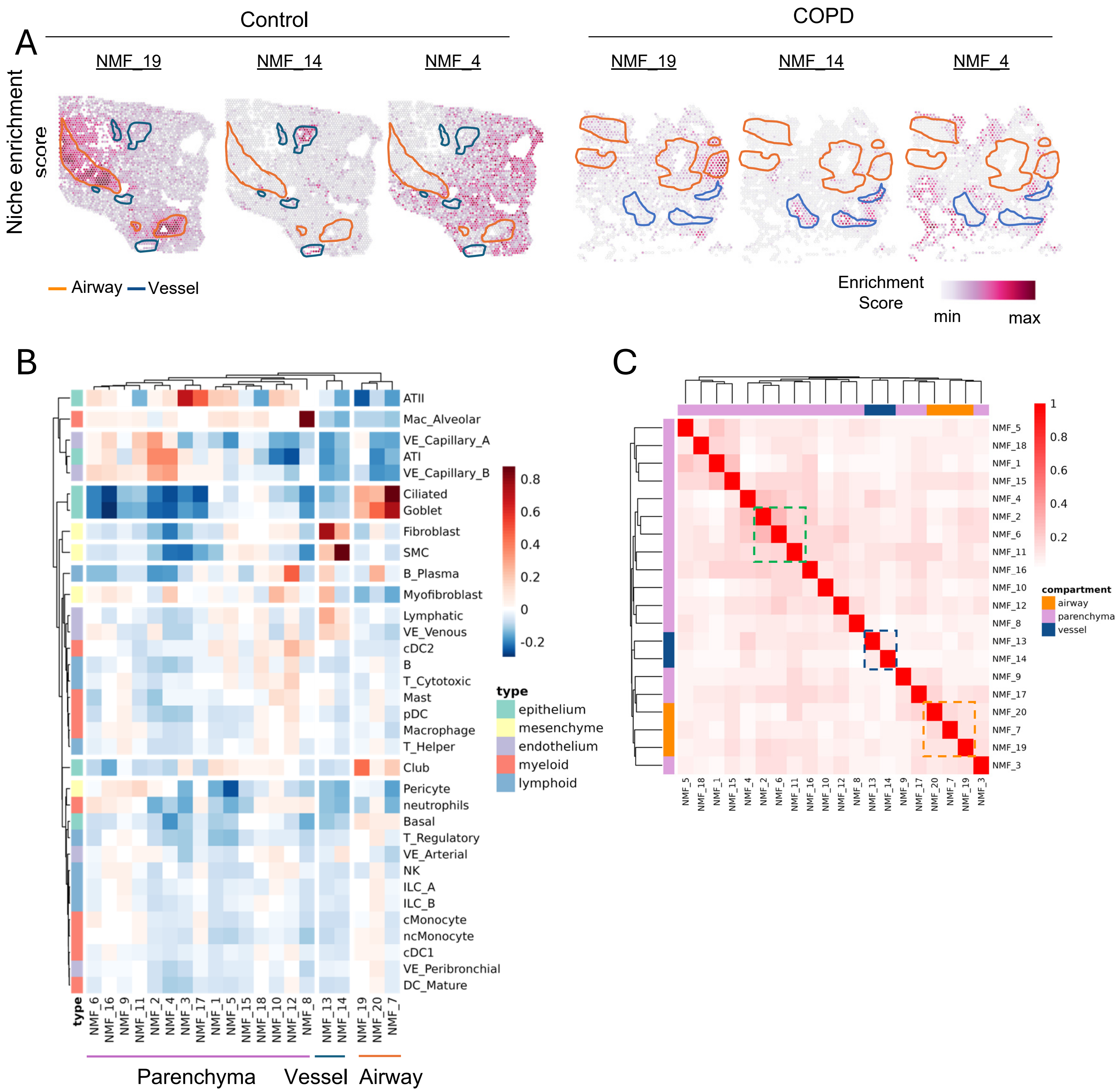

Figure S10: Unbiased discovery of spatial niches associated with lung compartments

(A) Spatial niches aligning with lung compartments; NMF_19 for airway, NMF_4 for parenchyma, and NMF_14 for vessels in selected control and COPD; colour represents niche enrichment score, airway and vessel areas were marked in orange and blue, respectively. (B) Correlation heatmap between deconvoluted cell type abundance and niche raw enrichment scores. Rows represent cell types and columns represents niches, colour indicating the strength and directionality of the correlation. (C) Heatmap showing Jaccard similarities across niches, calculated from top 100 driving genes, excluding ribosomal genes, on each niche. Rows and columns represent niches, annotated by compartments, with color indicating the degree of similarity.

Supplementary Figure S11

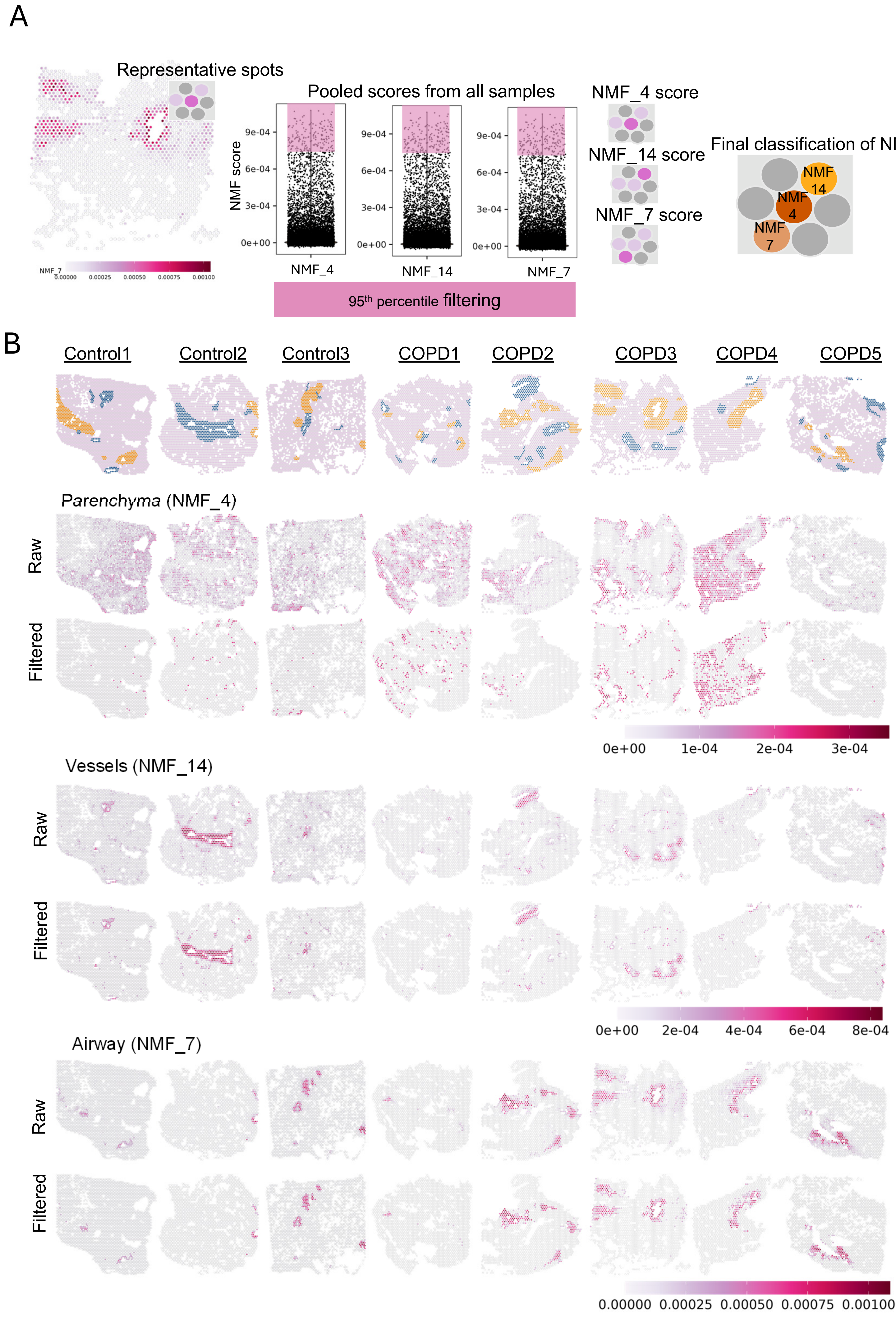

Figure S11: Pre-processing steps of spatial niche

(A) Schematics showing application of 95th percentile-filtering to determine enriched niches within a spot. Raw enrichment scores of a given niche from all spots were pooled and those were higher than 95th percentile were further compared with other niches. Certain spot was assigned to one niche based on the niche it highly expressed. (B) Spatial map of manually annotated compartment areas. Spatial map showing examples of raw and filtered niche scores of selected parenchyma (NMF_4), vessel (NMF_14), and airway (NMF_7) niche.

Supplementary Figure S12

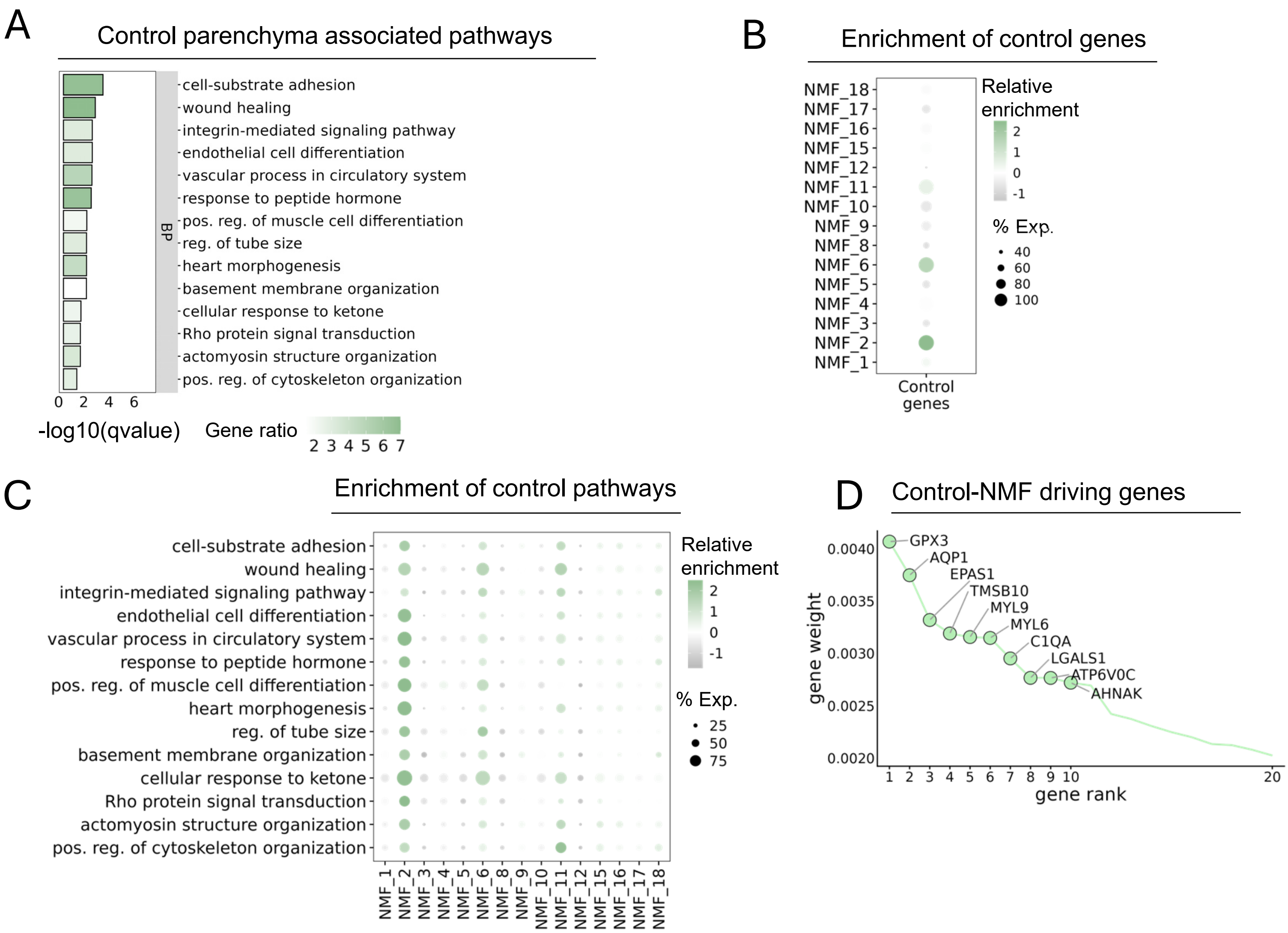

Figure S12: Control associated transcriptional changes and niches in parenchyma

(A) Enriched pathways in control parenchyma. Pathways were calculated using significantly downregulated genes (p_{adj} ≤0.1 & log2foldchange>0) in control parenchyma compared to COPD using clusterProfiler R package. Only the most significant pathways (qvalue ≤ 0.1) were shown; BP: Gene Ontology Biological Process. (B) Enrichment of control-associated genes across parenchymal niches. Scores for upregulated genes in control parenchyma were calculated using *AddModuleScore()* in Seurat. Dot size indicates the proportion of niche spots expressing the score, and colour represents relative enrichment scores across parenchymal niches. (C) Enrichment of control-associated pathways across parenchymal niches. Genes within each pathways were used as input to calculate the enrichment using *AddModuleScore()* in Seurat. Dot size indicates the proportion of niche spots expressing the score, and colour represents relative enrichment scores across parenchymal niches. (D) Top10 driving genes of Control-NMF, rank was defined based on the average scores of driving genes from NMF_2/6/11.

Supplementary Figure S13

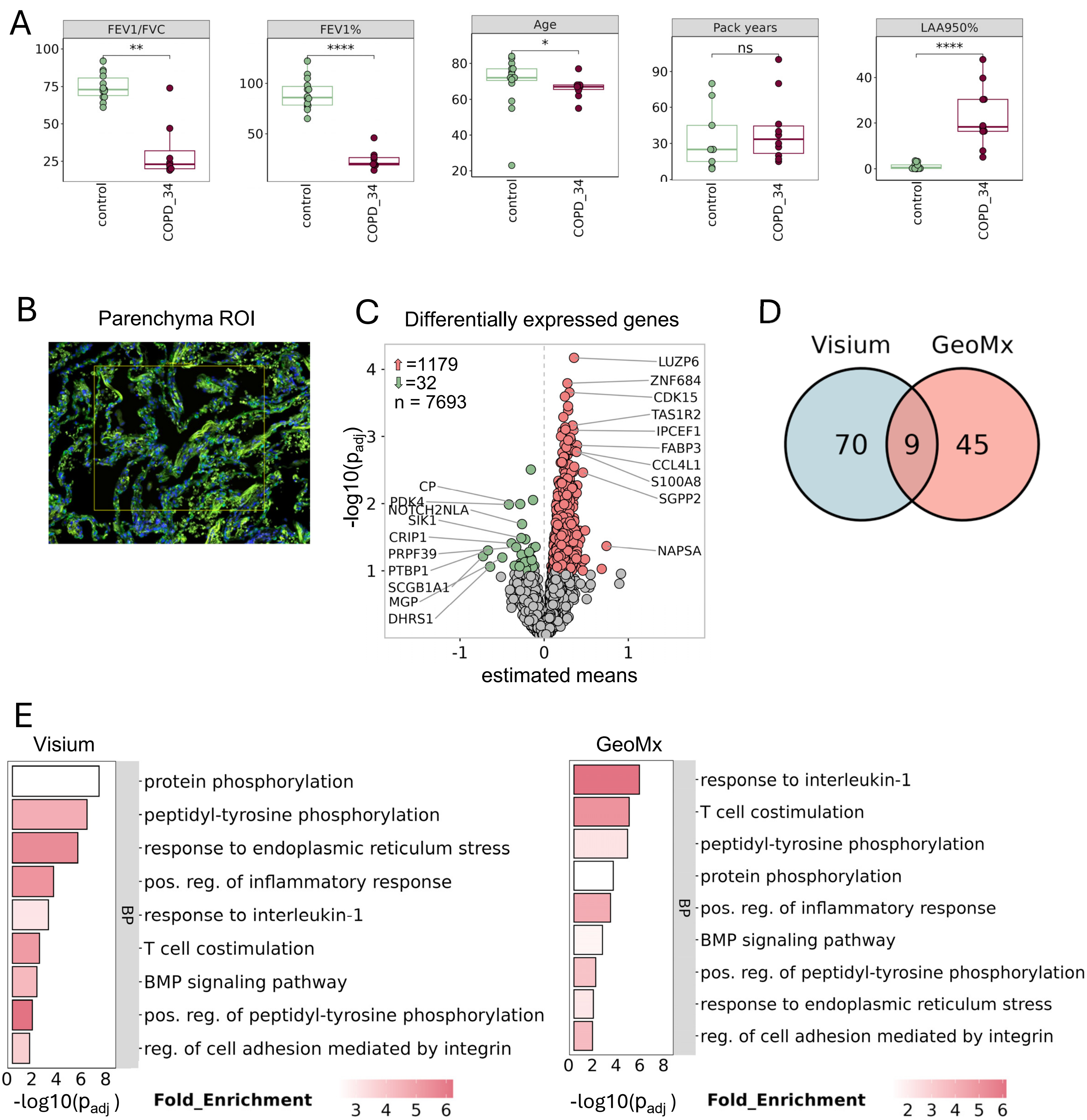

Figure S13: Independent validation of COPD signature pathways in parenchyma

(A) Comparison of clinical parameters (FEV1/FVC ratio, FEV1%, Age, smoking pack-years, LAA950%) between control and GOLD 3-4 COPD groups in GeoMx Nanostring Spatial Transcriptomic dataset; significance was calculated using Wilcoxon text (*p_{adj} ≤ 0.05, ** p_{adj} ≤ 0.01, *** p_{adj} ≤ 0.001, **** p_{adj} ≤ 0.0001). (B) Immunofluorescence staining showing a representative parenchyma region of interest (ROI) in GeoMx Nanostring spatial transcriptomics dataset; blue: Syto13, green: PanCK, adapted from [Bordag et al., iScience, 2025], licensed under CC BY (C) Differentially expressed genes between COPD and control parenchyma ROIs. Differential expression analysis was performed using linear mixed model with individual sample as random effect and disease as fixed effect. Only significant genes (p_{adj} ≤0.1), adjusted by fdr, were colored and top 10 genes based on significance and log2foldchange were labelled. (D) Venn diagrams and (E) bar plot showing shared deregulated pathways between Visium spatial transcriptomics and GeoMx dataset. Pathways were calculated using significantly deregulated genes (p_{adj} ≤0.1) between COPD and controls independently on Visium (left panel) and GeoMx dataset (right panel) using PathfindR package. Only the shared significant pathways (p_{adj} ≤ 0.1) were shown; BP: Gene Ontology Biological Process.

Supplementary Figure S14

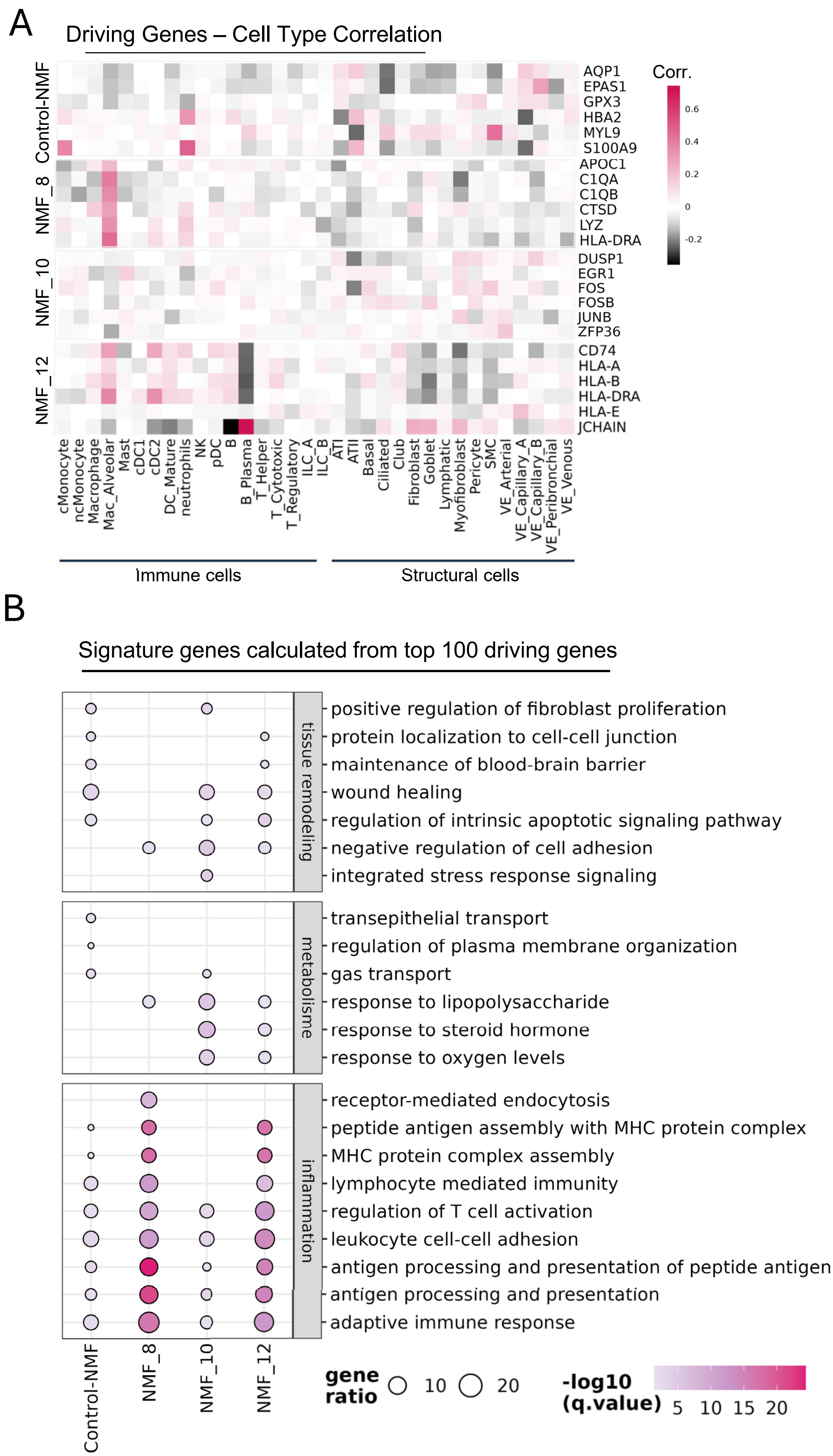

Figure S14: Driving genes and pathways of parenchymal niches

(A) Correlation heatmap between driving gene (columns) expressions and cell type (rows) abundance within parenchyma niches, colour indicates the strength and directionality of the correlation, calculated with pearson method (B) Parenchyma niches exhibited shared and distinct biological processes. Selected most significantly enriched pathways, were calculated from top 100 driving genes, excluding ribosomal genes, using overrepresentation analysis in clusterProfiler R package. Only significant pathways ($p_{adj} \leq 0.05$, calculated with Benjamini-Hochberg) were shown. Colour indicates the significance level and dot size represents the ratio between enriched genes and gene set.

Supplementary Figure S15

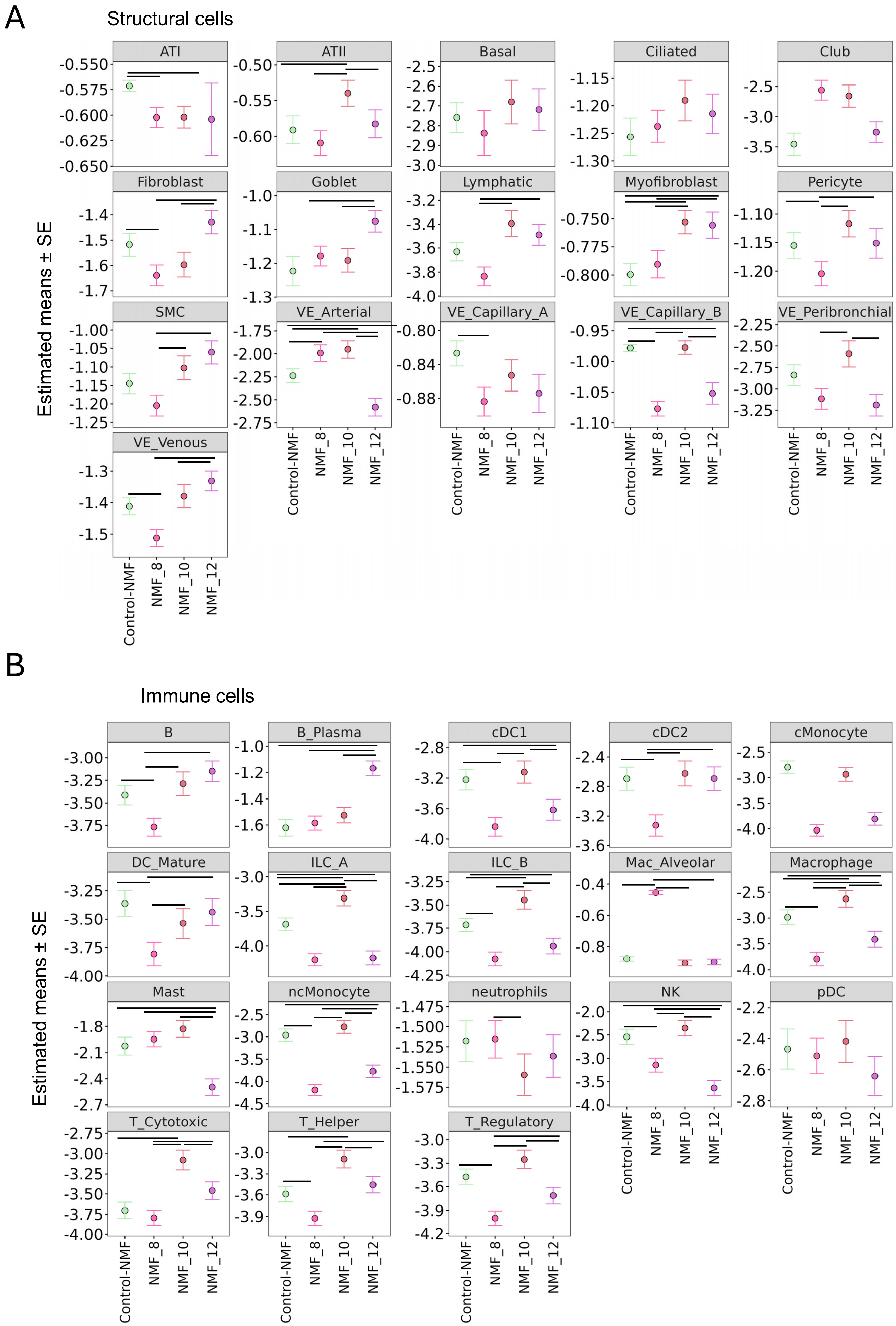

Figure S15: Cellular signatures across control and COPD-associated parenchyma niches

(A) Quantification of structural and (B) immune cells across pooled control niches (NMF_2/6/11, Control-NMF) and COPD abundant niches (NMF_8/10/12). Significance was calculated with linear mixed model with individual sample as a random effect, niche as a fixed effect. P-values were adjusted with fdr; lines indicate $p_{adj} \leq 0.1$. Data was represented with estimated means ± standard errors.

Supplementary Figure S16

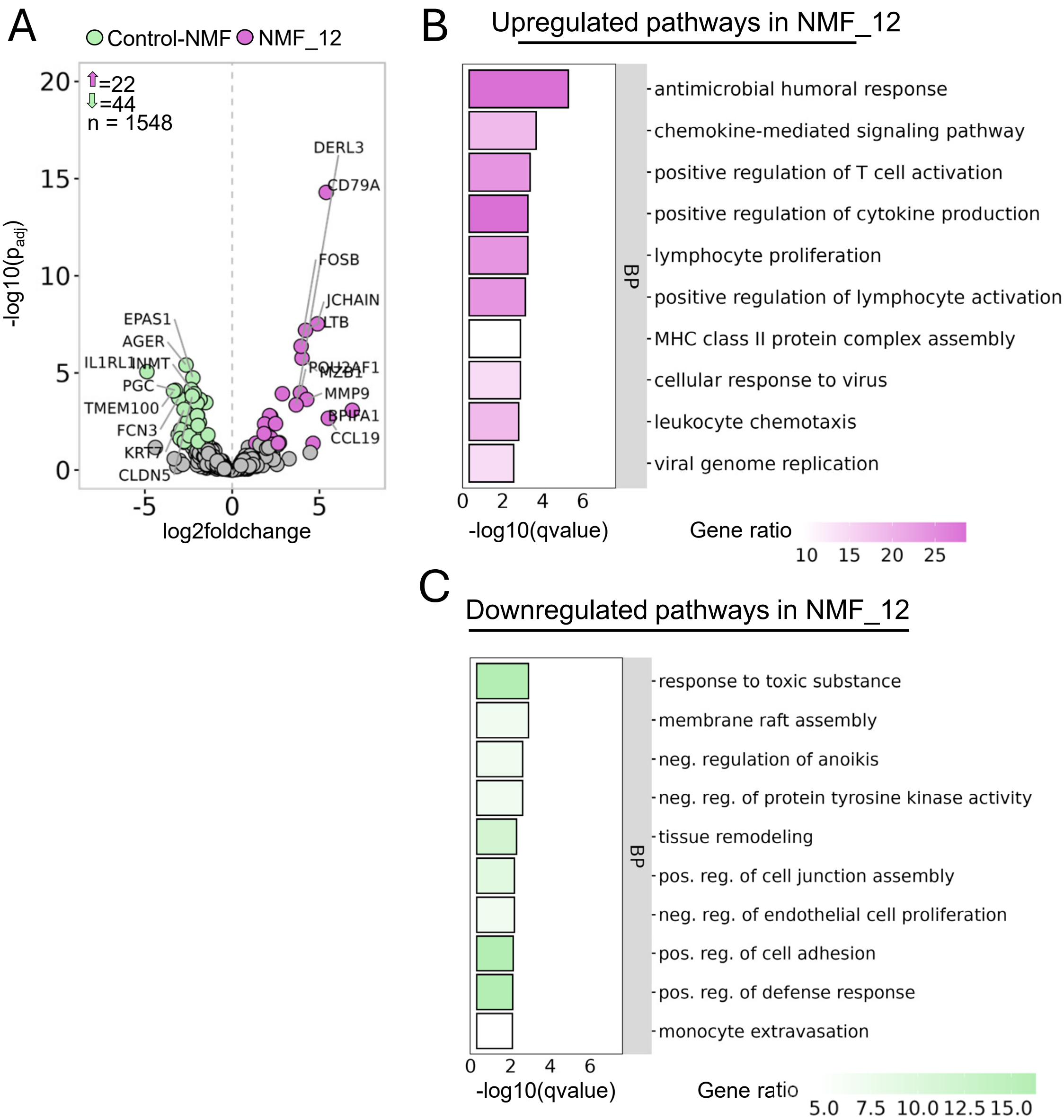

Figure S16: Disease associated transcriptional changes in humoral immune niche

(A) Differentially expressed genes between COPD and controls in humoral immune niche (NMF_12). (A) Volcano plot showing differentially expressed genes between NMF_12 and Control-NMF, calculated using DESeq2 R package. Only significant genes (p_{adj} ≤0.05) were colored and top 10 genes based on significance and log2foldchange level were labelled. (B) Bar plot of selected most significantly enriched pathways (qvalue ≤0.05) calculated from upregulated genes (p_{adj} ≤0.05 & log2foldchange>0) and (C) downregulated genes (p_{adj} ≤0.05 & log2foldchange<0). X axis represents the significance level and colour of the bar represents the ratio between expressed genes and gene set size; BP: Gene Ontology Biological Process.

Supplementary Figure S17

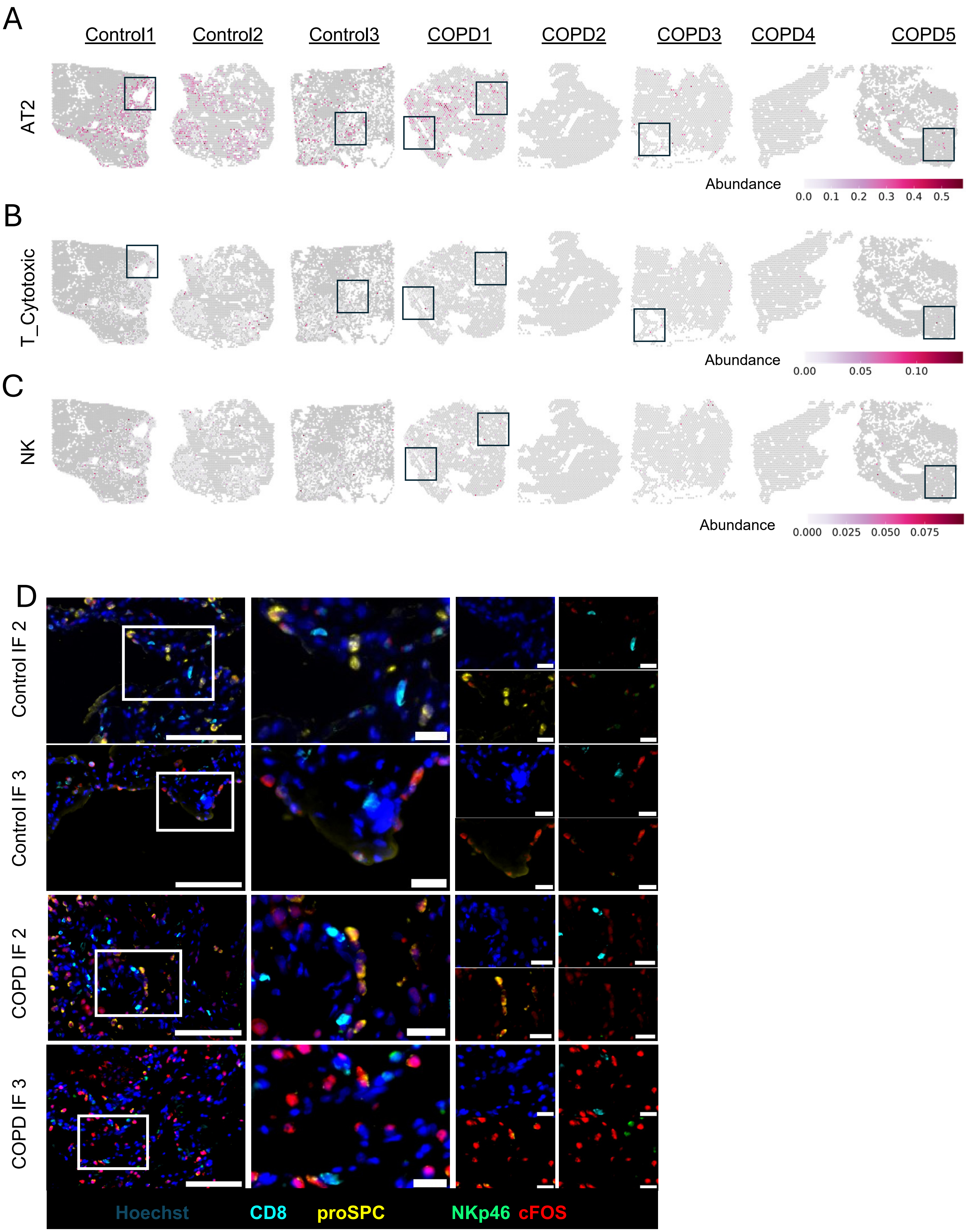

Figure S17: Spatial colocalization between AT2, NK, and cytotoxic T cells within control-NMF and remodelling-stress niche.

(A) Spatial map showing spot proportion of deconvoluted AT2, (B) NK, and (C) cytotoxic T cells within control-NMF and remodelling-stress niche (NMF_10) across control and COPD samples. Colocalization was shown in selected areas (squared boxes). Colour represents cell type ssociated transcript abundance. (D) Multiplex immunofluorescence stainings of FOS (cFOS^{+}, red) on AT2 cells (ProSPC^{+}, yellow), cytotoxic T cells (CD8^{+}, turquoise), and NK cells (NKp46^{+}, green) within control and COPD parenchyma. Scale bars: 100 µm (overview panels), 20 µm (zoomed panels).

Supplementary Figure S18

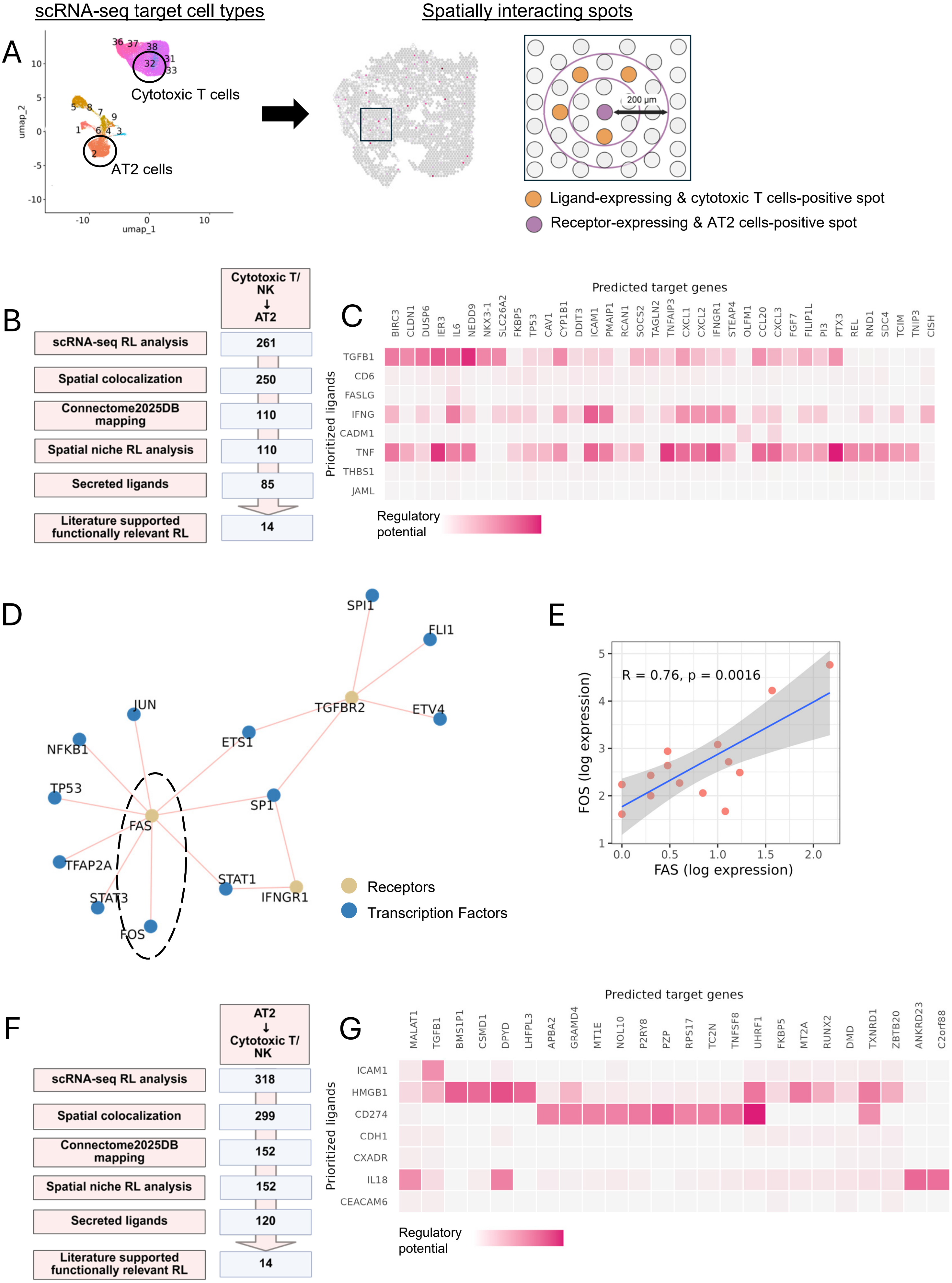

Figure S18: Predicted downstream targets and transcription factor networks in AT2–NK/cytotoxic T cell interactions

(A) Schematics showing workflow of scRNA-seq based ligand receptor analysis followed by identification of spatially interacting spots. Ligand-receptor gene pairs were filtered based on the spatial proximity (200 μm from ligand expressing spot). (B) Workflow of identification target receptor-ligand interactions between cytotoxic T cells or NK and AT2 cells. (C) Heatmap showing the regulatory potential of functionally relevant ligands on downstream target genes as a results of ligand and receptor interactions in AT2 cells. Target genes were calculated on integrated scRNA dataset (GSE136831, GSE171541) and predicted based on differentially expressed genes between COPD and controls in AT2 cells and known protein-protein interaction database applied in nichenetr R package. (D) Transcription factor (TF) analysis showed regulatory network of prioritized receptor genes based on Figure 3C in AT2 cells. Edge represents connection between TF and target genes, and blue nodes correspond to TF while brown nodes represent receptor genes. FAS-FOS connection was shown in dotted line. (E) Dot plot showing correlation on aggregated FAS and FOS gene expressions in AT2 cells of COPD scRNA datasets. Correlation was calculated with pearson method. (F) Workflow of identification target receptor-ligand interactions between AT2 cells and cytotoxic T cells or NK. (G) Heatmap showing the regulatory potential of functionally relevant ligands on downstream target genes as a results of ligand and receptor interactions in cytotoxic T and NK cells. Target genes were calculated on integrated scRNA-seq dataset (GSE136831, GSE171541) and predicted based on differentially expressed genes between COPD and controls in cytotoxic T and NK cells and known protein-protein interaction database applied in nichenetr R package.

Supplementary Figure S19

A IFN-γ production on CD8⁺ cytotoxic T cells

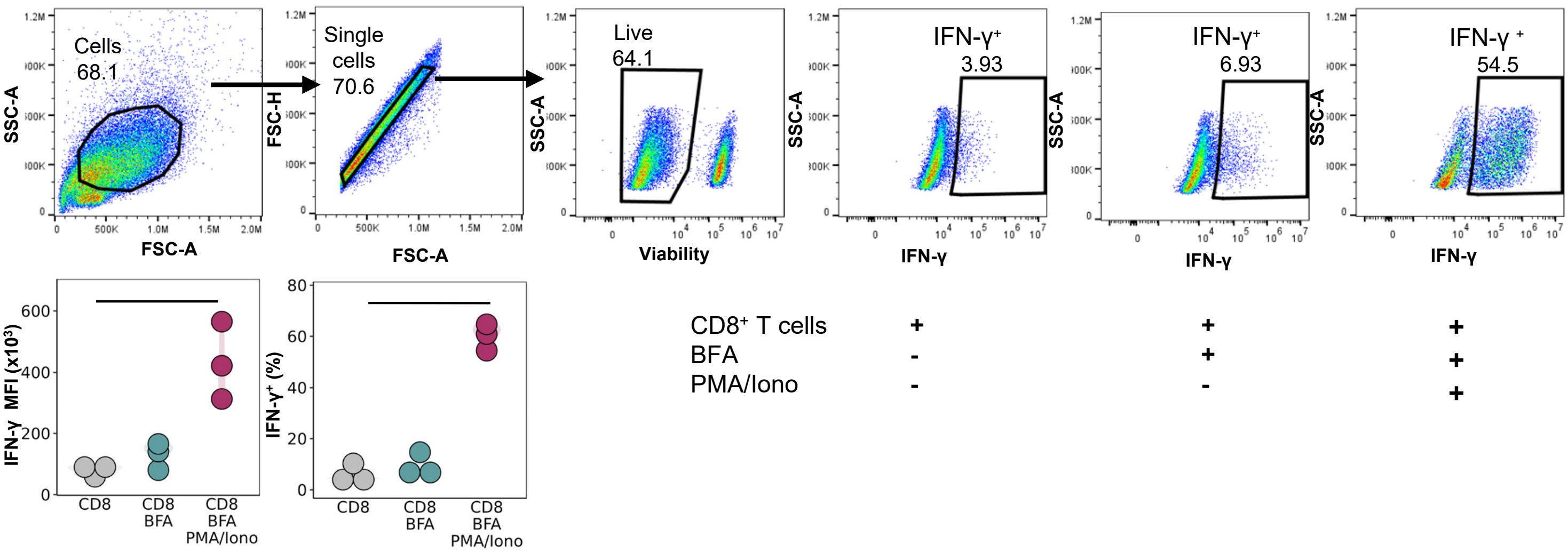

B Epithelial pSTAT1 activation

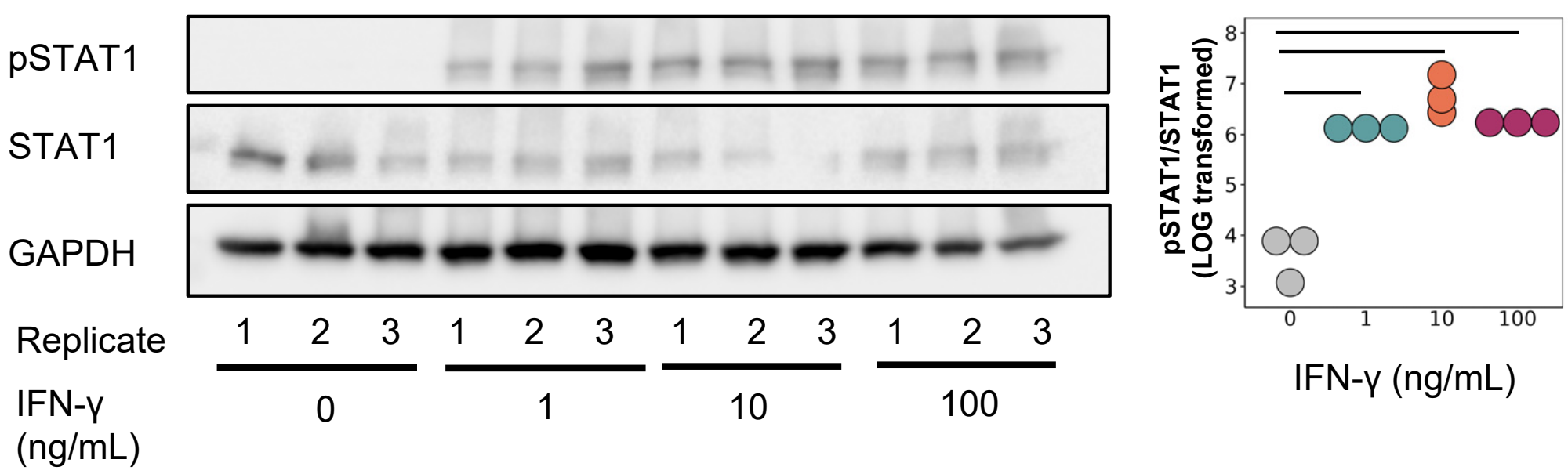

C Epithelial pSTAT1 activation cultured with CD8⁺ T cells

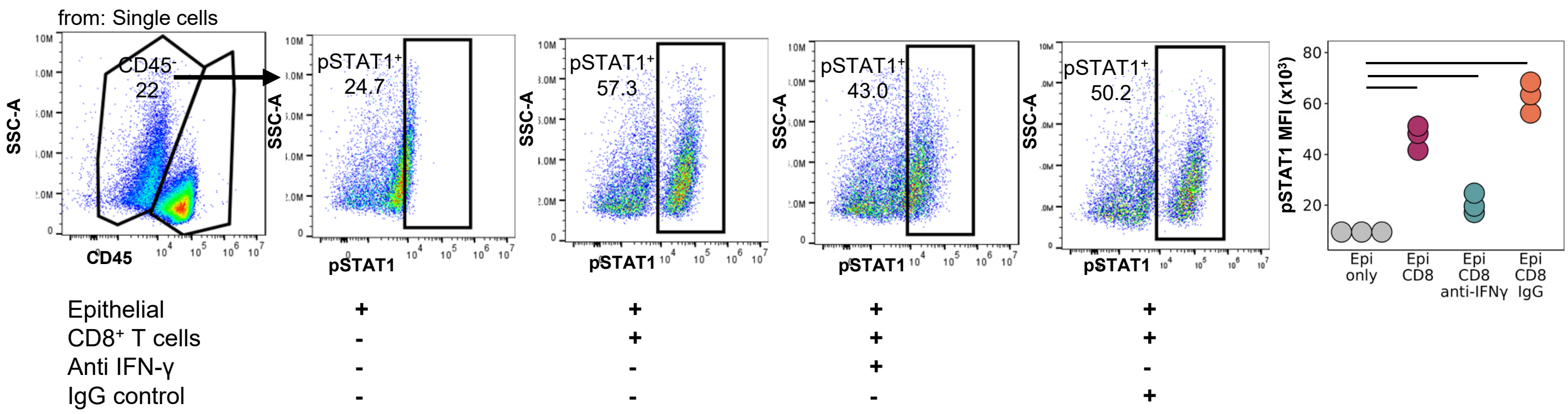

Figure S19: In vitro validation of IFNG-IFNGR1 interaction

(A) Gating strategy (top panel) to identify IFN-γ producing $CD8^+$ cytotoxic T cells from human blood (n=3), after 4-day activation with anti CD3/CD28 followed by 4.5h incubation with PMA/Ionomycin and an addition of protein transport inhibitor (BFA) after 2 h or BFA only after 2 h. Dot plot (bottom panel) showing median fluorescence of intensity (MFI) of IFN-γ in IFN-$\gamma^+$$CD8^+$ cytotoxic T cells and percentage positive population across different treatment groups. Statistical significance was one-way ANOVA followed by Dunnett's multiple-comparison test, lines indicate p_{adj} ≤0.05.(B) Phosphorylation of STAT1, in epithelial cells (A549 cells) following stimulation with IFN-γ (0,1,10,100 ng/mL) for 15 mins. Dot plot (right panel) showing log-transformed optical densities of pSTAT1 protein bands in arbitrary units. Statistical significance was one-way ANOVA followed by Dunnett's multiple-comparison test, lines indicate p_{adj} ≤0.05. (C) Gating (left panel) to to identify activation STAT1 ($pSTAT1^+$) in epithelial cells (A549 cells), following co-culture with pre-activated $CD8^+$ cytotoxic T cells (n=3) for 0.5 h, with or with or without additionally blocking with the presence anti IFN-γ (10 µg/mL) or IgG control (10 µg/mL). Dot plot (right panel) showing MFI of STAT1 in $pSTAT1^+$epithelial cells. Statistical significance was one-way ANOVA followed by Dunnett's multiple-comparison test, lines indicate p_{adj} ≤0.05.

Supplementary Figure S20

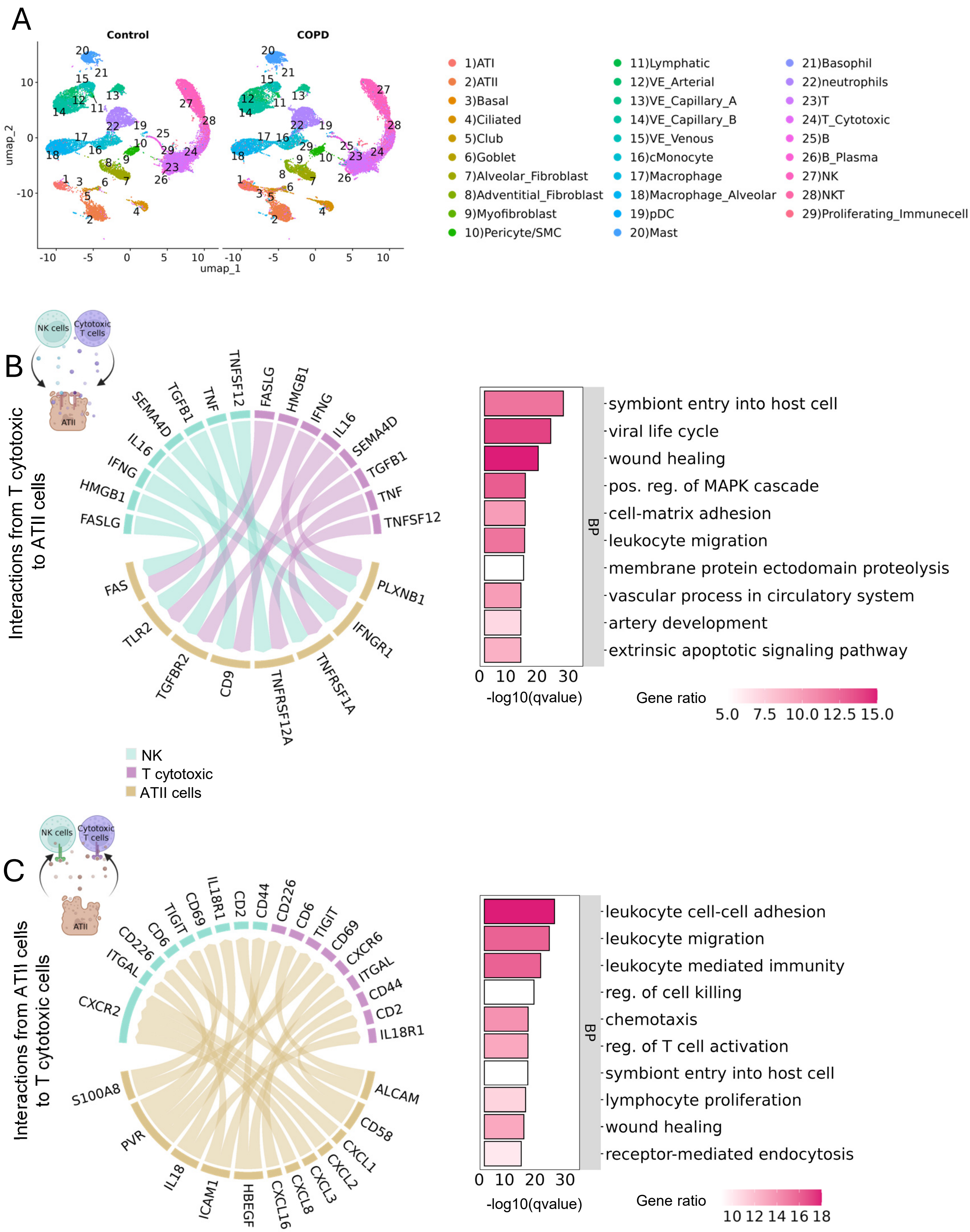

Figure S20: Cytotoxic T cells are predicted to interact with AT2 cells in GOLD1-2 COPD lungs

(A) UMAP showing cell type annotated in scRNA-seq (GSE173896) dataset. (B) Ligand–receptor interactions inferred using the nichenetr R package on control and GOLD1-2 COPD scRNA-seq datasets (GSE173896). Circos plot showing prioritised ligands expressed by NK or cytotoxic T cells and their corresponding receptors on AT2 cells. Ligand–receptor pairs were filtered to retain those shown in Fig. 3C-D. Colours indicate cell types; directed edges represent signalling direction from sender to receiver. Bar plot of significantly enriched pathways (qvalue ≤ 0.05) derived from all identified receptors and target genes. Colour represents the ratio of expressed genes to gene set size, and the x-axis indicates significance. BP denotes Gene Ontology Biological Process. Violin plots show RNA expression of selected ligand or receptor genes across AT2 and cytotoxic T cells in COPD samples. Significance was assessed using Wilcoxon tests with FDR correction (* $p_{adj} \leq 0.05$, ** $p_{adj} \leq 0.01$, *** $p_{adj} \leq 0.001$, **** $p_{adj} \leq 0.0001$). (C) Circos plot of ligand–receptor interactions with AT2 cells as signal senders and NK or cytotoxic T cells as receivers, inferred using nichenetr and were filtered to retain those shown in Fig. 3E-F. Enriched pathways (qvalue ≤ 0.05) derived from all detected receptors and target genes, with corresponding violin plots showing expression of selected genes across AT2 and cytotoxic T cells in COPD.

Supplementary Figure S21

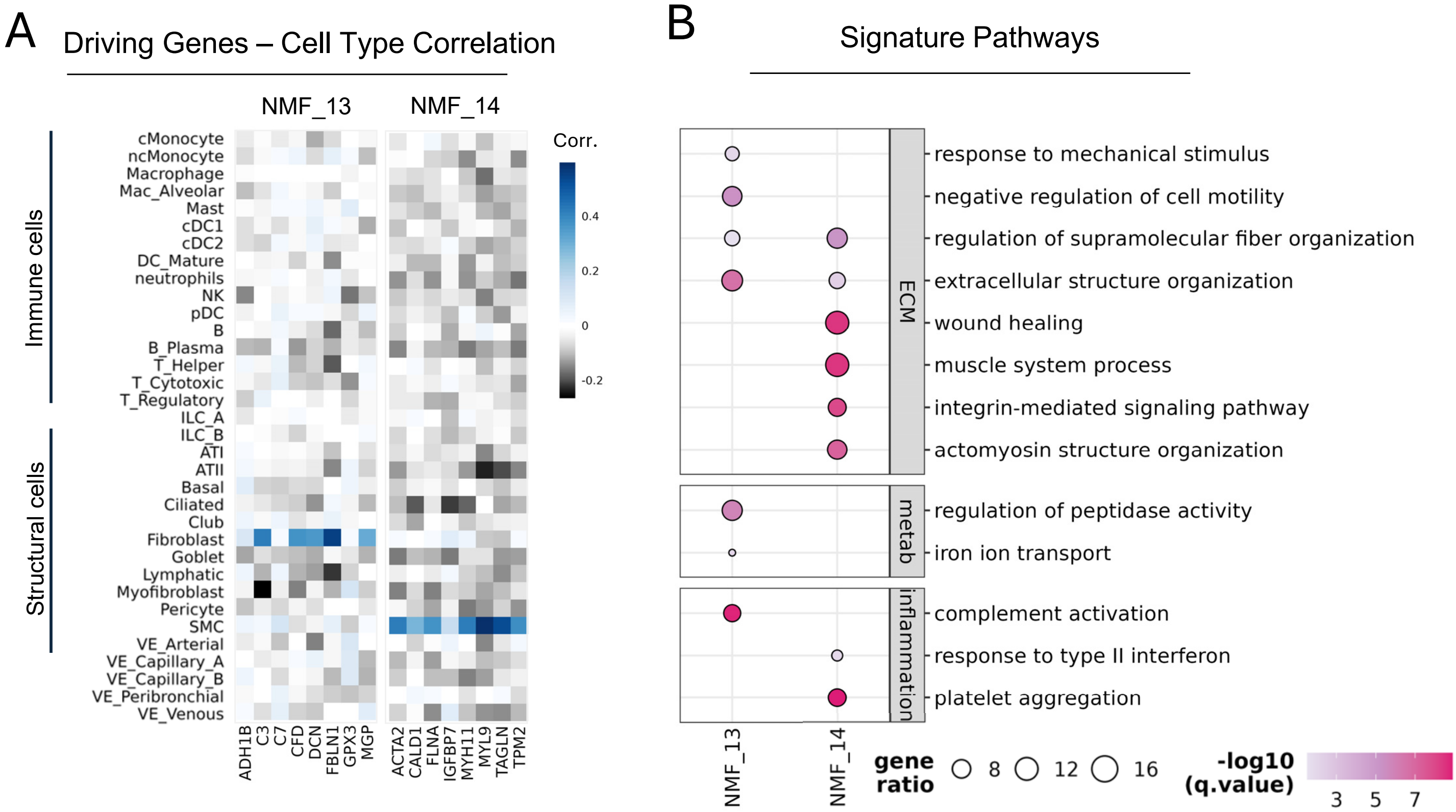

Figure S21: Driving genes and pathways of vessel niches

(A) Correlation heatmap between driving gene (columns) expressions and cell type (rows) abundance within vessel niches, colour indicates the strength and directionality of the correlation, calculated with pearson method (B) Vessel niches exhibited shared and distinct biological processes. Selected most significantly enriched pathways, were calculated from top 100 driving genes, excluding ribosomal genes, using overrepresentation analysis in clusterProfiler R package. Only significant pathways (qvalue ≤0.05, calculated with Benjamini-Hochberg) were shown. Colour indicates the significance level and dot size represents the ratio between enriched genes and gene set; BP: Gene Ontology Biological Process; metab: Metabolism.

Supplementary Figure S22

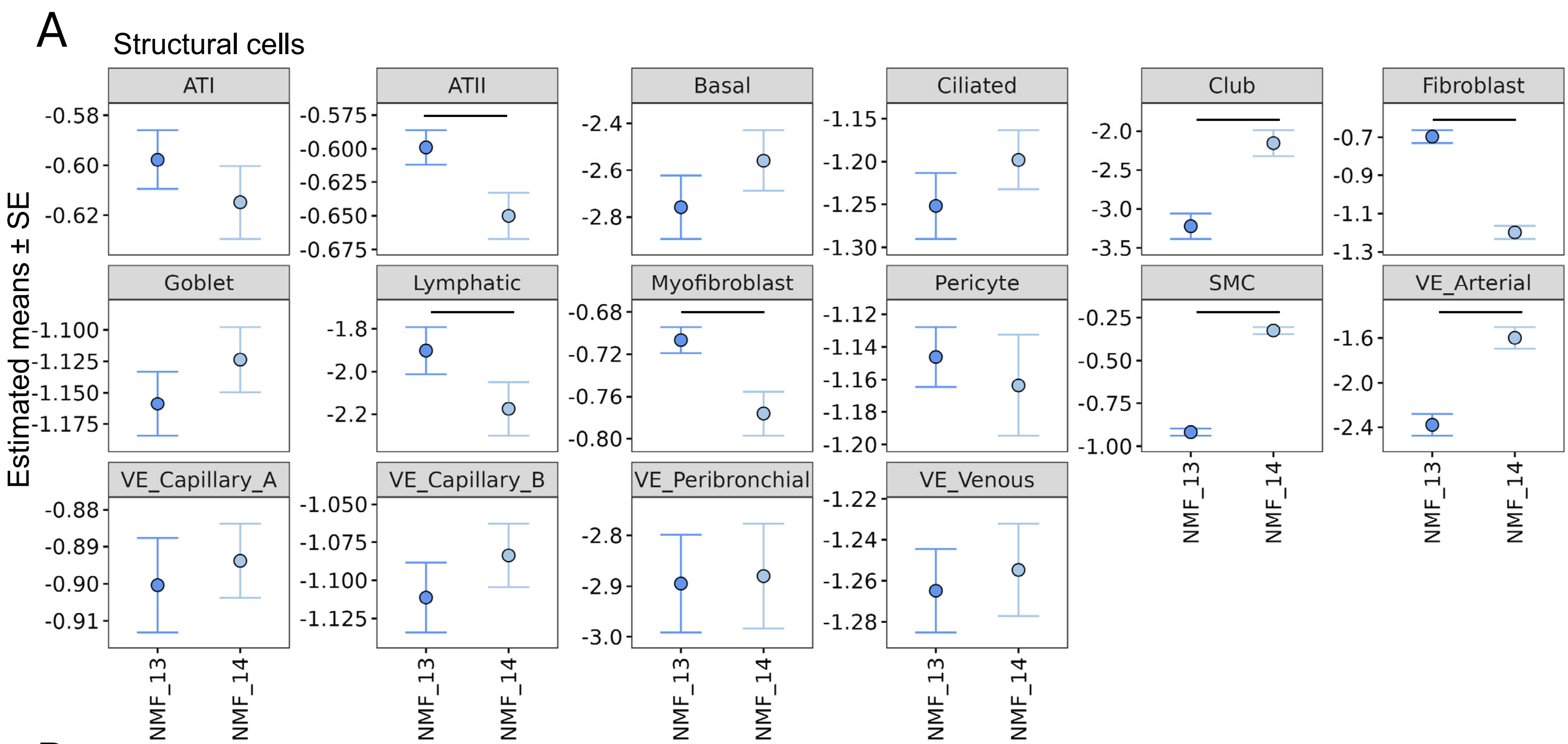

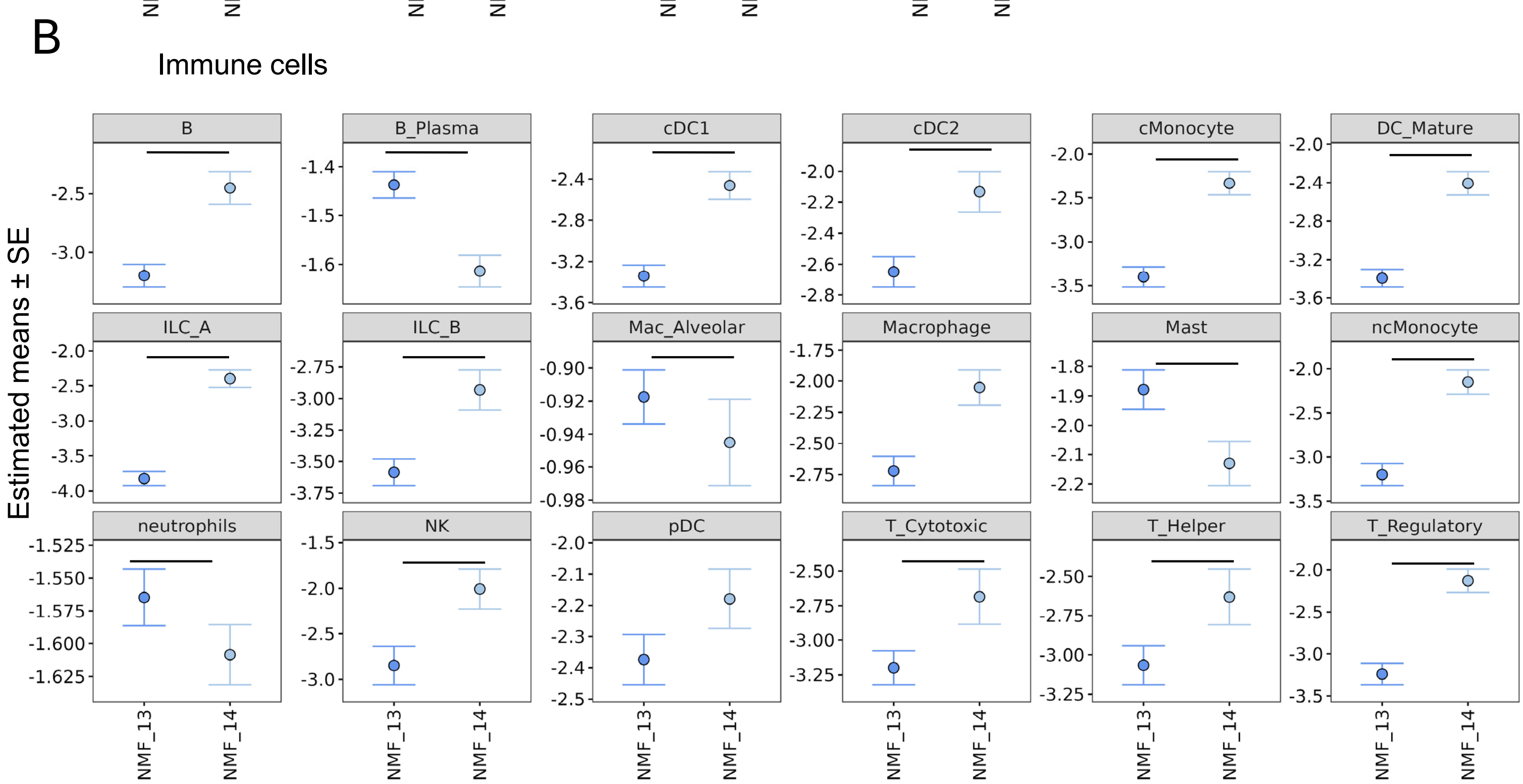

Figure S22: Cellular signatures across vessel niches

(A) Quantification of structural and (B) immune cells across perivascular (NMF_13) and vascular niches (NMF_14). Significance was calculated with linear mixed model with individual sample as a random effect, niche as a fixed effect. P-values were adjusted with fdr; lines indicate p_{adj} ≤0.05. Data was represented with estimated means ± standard errors.

Supplementary Figure S23

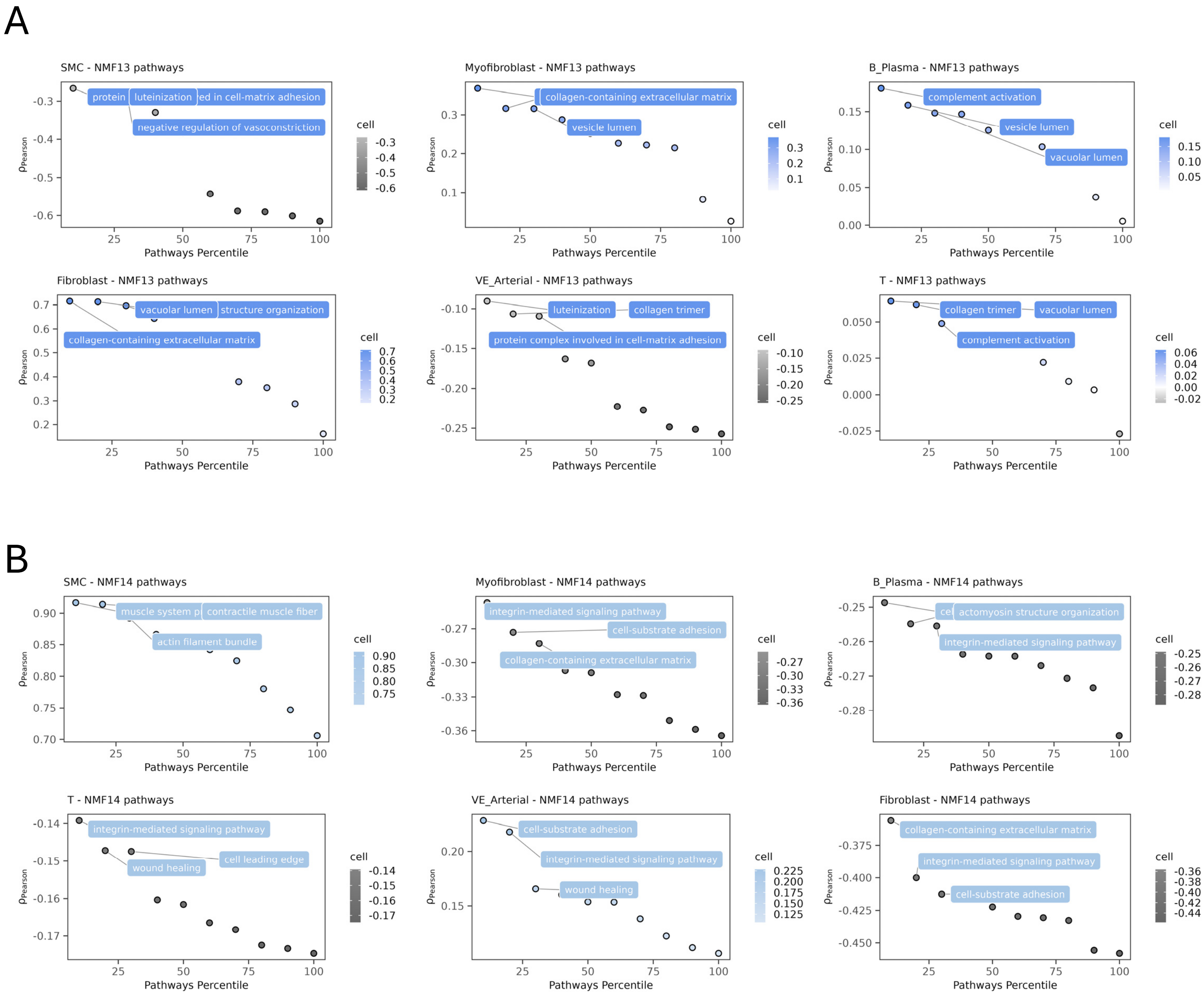

Figure S23: Cell type associateion with signature pathways across vessel niches

(A) Correlation between deconvolution scores of selected cell types with enrichment scores of vascular (NMF_13) and (B) perivascular (NMF_14) niches pathways. Pathway scores were computed using *Addmodulescore()* function from Seurat package based on genes identified within each corresponding pathway in Figure S14B. Correlation was calculated using pearson method on pooled vessel niches spots.

Supplementary Figure S24

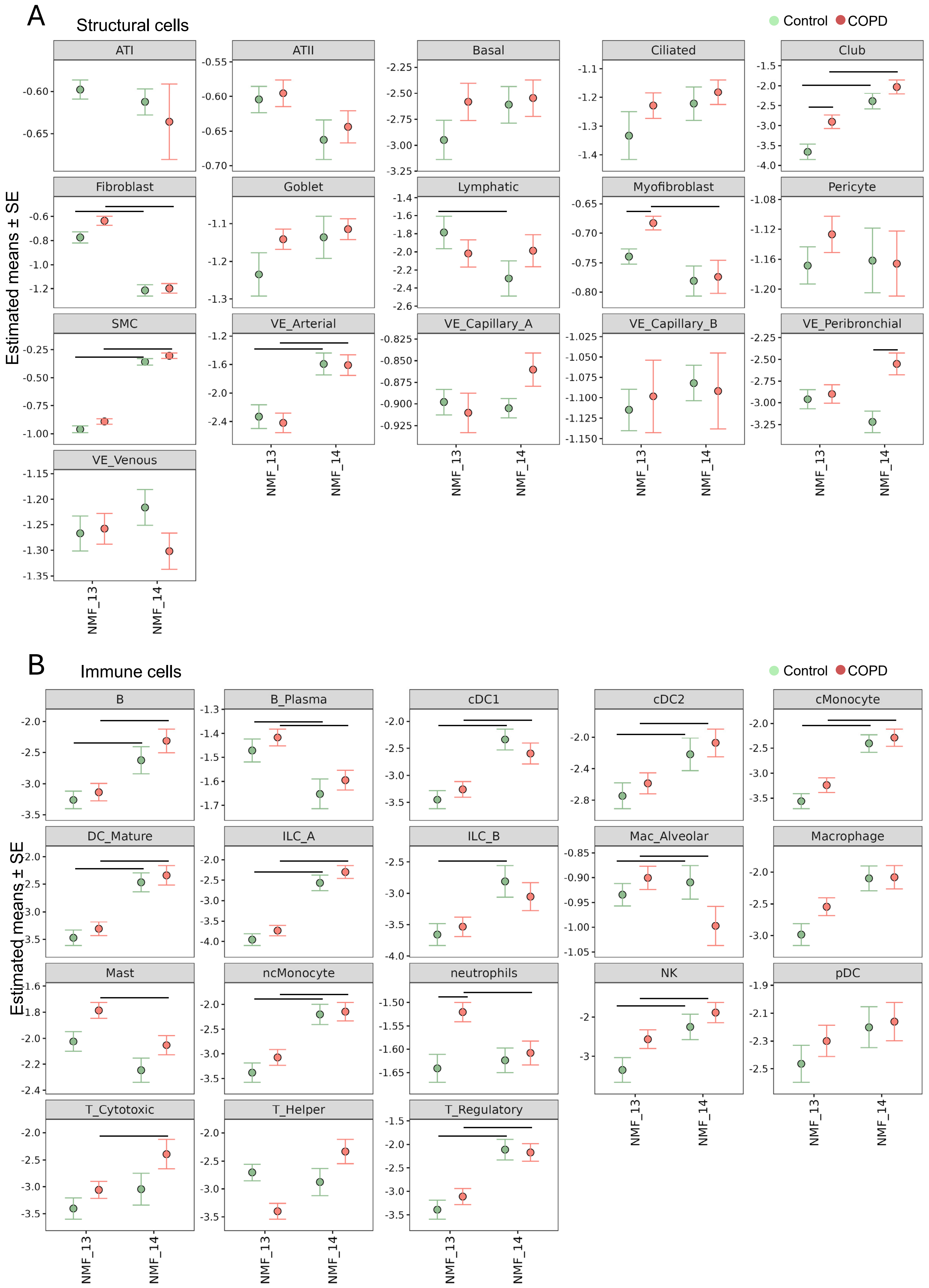

Figure S24: Disease associated deregulated cell populations across vessel niches

(A) Quantification of selected structural and (B) immune cells in control and COPD across perivascular (NMF_13) and vascular (NMF_14) niches. Significance was calculated with linear mixed model with individual sample as a random effect, diagnosis and niches as fixed effects. P-values were adjusted with fdr; lines indicate $p_{adj} \leq 0.1$. Data was represented with estimated means ± standard errors.

Supplementary Figure S25

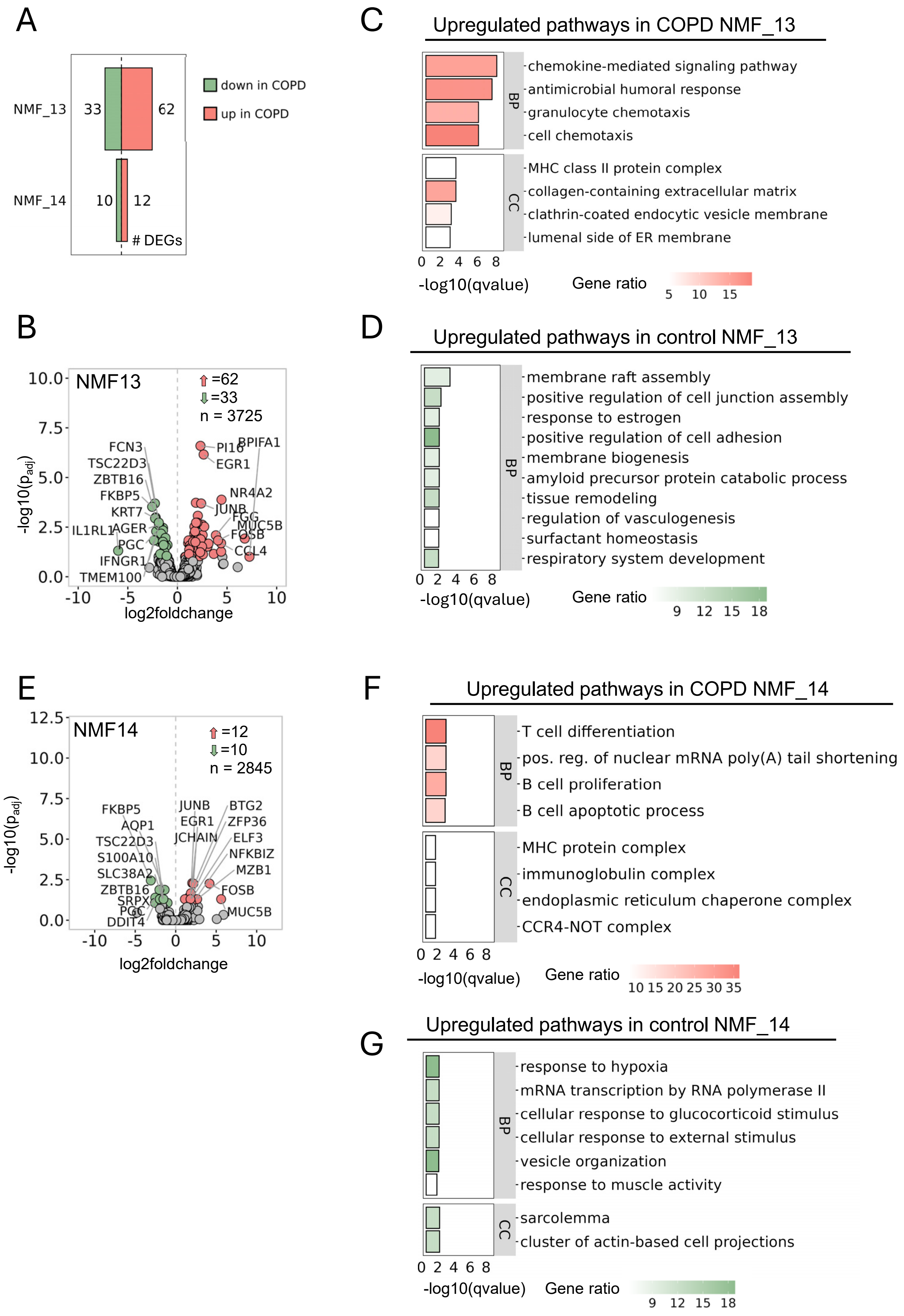

Figure S25: Disease associated transcriptional changes across vessel niches

(A) Bar chart showing number of differentially expressed genes between control and COPD across perivascular (NMF_13) and vascular (NMF_14) niches. Colour represents the direction of regulation. Differentially expressed genes (p_{adj} ≤0.1) between COPD and controls, calculated independently in NMF_13 and NMF_14. (B) Volcano plot showing differentially expressed genes between control and COPD in NMF_13, calculated using DESeq2 R package. Only significant genes (p_{adj} ≤0.1, adjusted by Benjamini–Hochberg method) were coloured and top 10 genes based on significance and log2foldchange level were labelled. (C) Bar plot of selected most significantly enriched pathways (qvalue ≤0.1) calculated from significantly upregulated genes (p_{adj} ≤0.1 & log2foldchange >0) (D) and significantly downregulated genes (p_{adj} ≤0.1 & log2foldchange >0) in NMF_13. Colour represents the ratio between expressed genes and gene set size and x-axis represents the significance level. BP: Gene Ontology Biological Process, CC: Gene Ontology Cellular Component. (E) Volcano plot showing differentially expressed genes between control and COPD in NMF_14, calculated using DESeq2 R package. Only significant genes (p_{adj} ≤0.1, adjusted by Benjamini–Hochberg method) were coloured and top 10 genes based on significance and log2foldchange level were labelled. (F) Bar plot of selected most significantly enriched pathways (qvalue ≤0.1) calculated from significantly upregulated genes (p_{adj} ≤0.1 & log2foldchange >0) (G) and significantly downregulated genes (p_{adj} ≤0.1 & log2foldchange >0) in NMF_14. Colour represents the ratio between expressed genes and gene set size and x-axis corresponds to the significance level.

Supplementary Figure S26

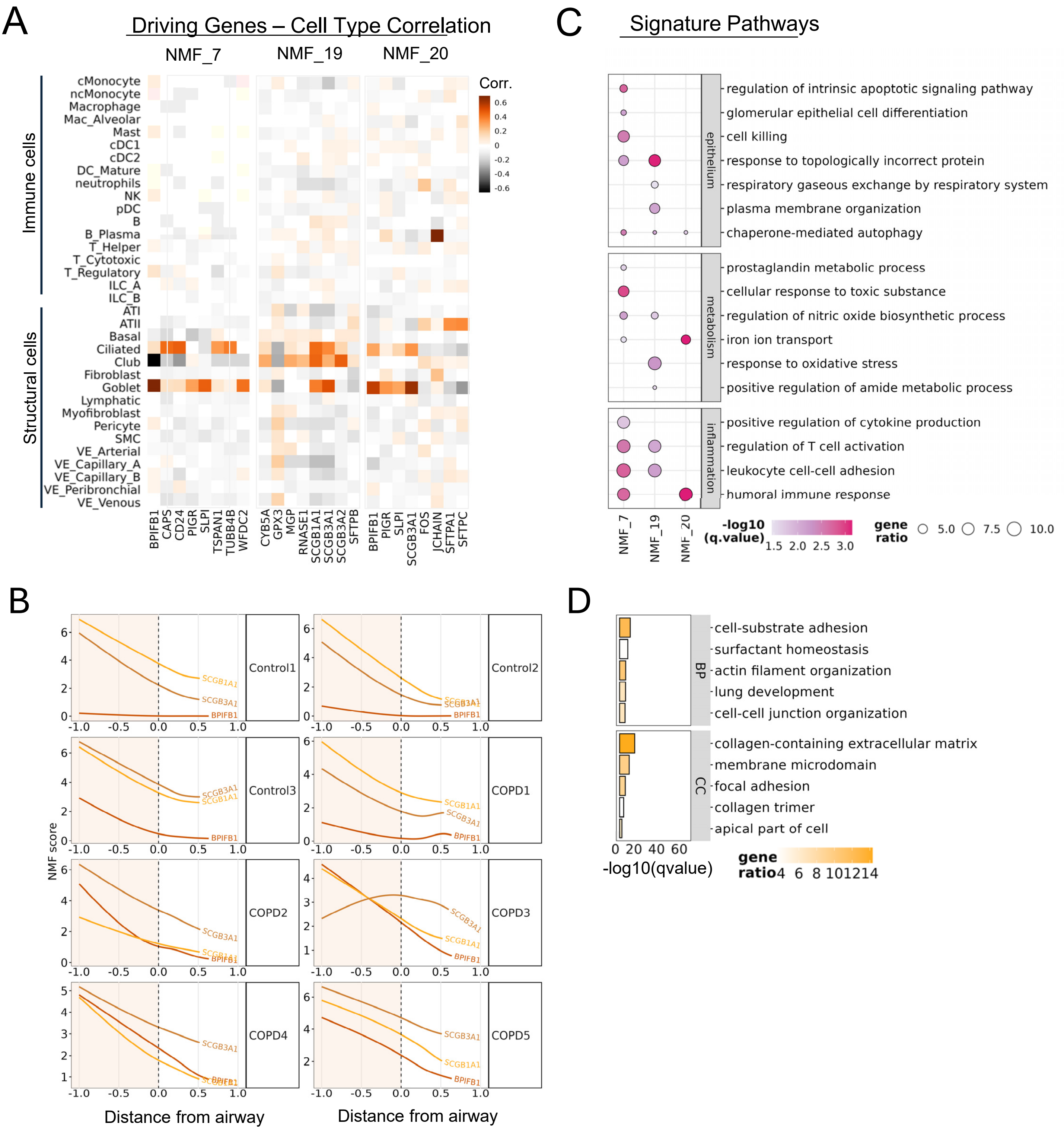

Figure S26: Signature genes and pathways of airway niches

(A) Correlation heatmap between top driving gene (columns) expressions and cell type (rows) abundance within COPD-endobronchial (NMF_7), control-endobronchial (NMF_19), and COPD-peribronchial (NMF_20) niches, color indicates the strength and directionality of the correlation, which was calculated using pearson method (B) Dot plot showing selected significantly enriched pathways (p_{adj} ≤0.05), calculated from top 100 driving genes excluding ribosomal genes, within each airway niche using clusterProfiler R package. Colour indicates the significance level and dot size represents the ratio between enriched genes and total number of genes in a given gene set. (C) Expression levels of selected top driving genes from inside to outside manually annotated airway areas across control and COPD samples. Negative values represent airway spots, positive values correspond to the neighboring spots (radius of 2.5 mm), and zero values represents the border spots between airway and non-airway areas. (D) Bar chart of selected significantly enriched pathways (p_{adj} ≤0.05) of control-endobronchial niche. Pathway enrichment analysis was performed on downregulated genes (p_{adj} ≤0.05 & log2foldchange<0) in Figure 5D using clusterProfiler R package; BP: Gene Ontology Biological Process, CC: Gene Ontology Cellular Component.

Supplementary Figure S27

A

Structural cells

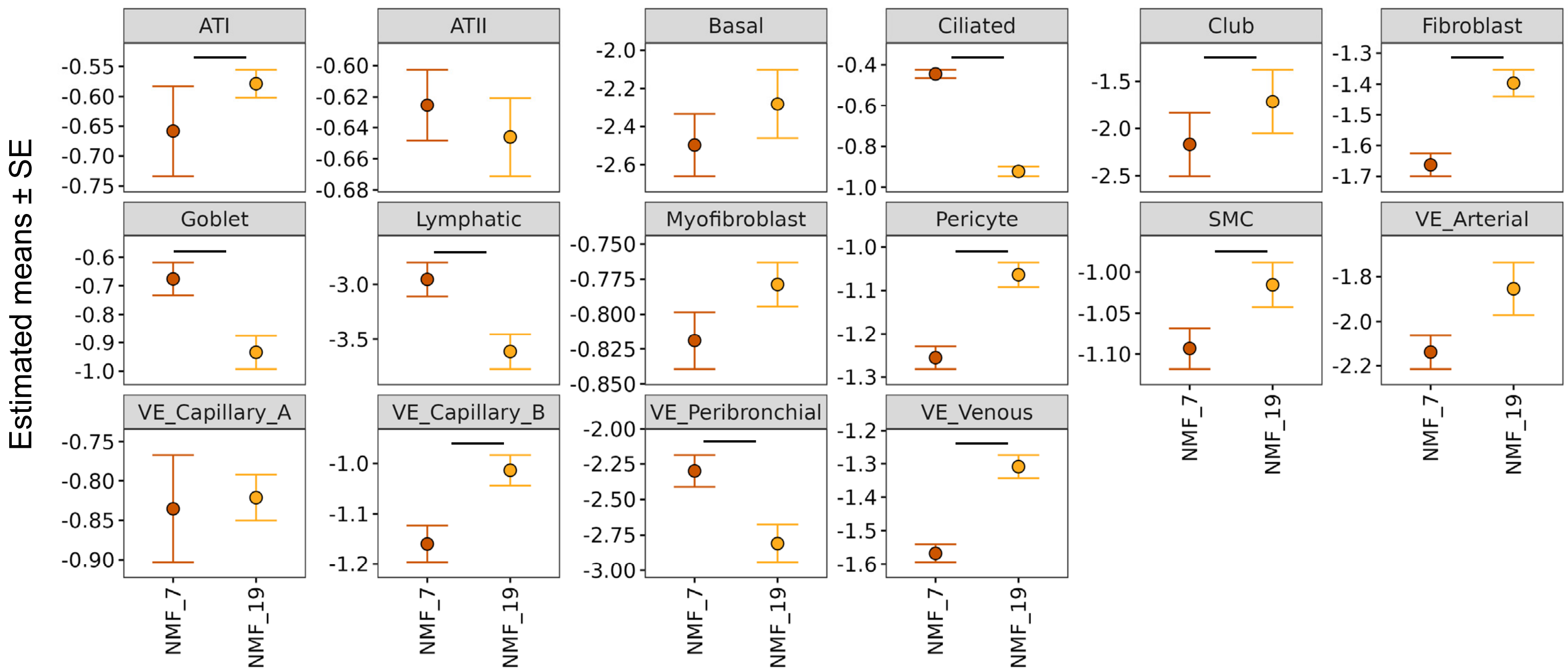

B

Immune cells

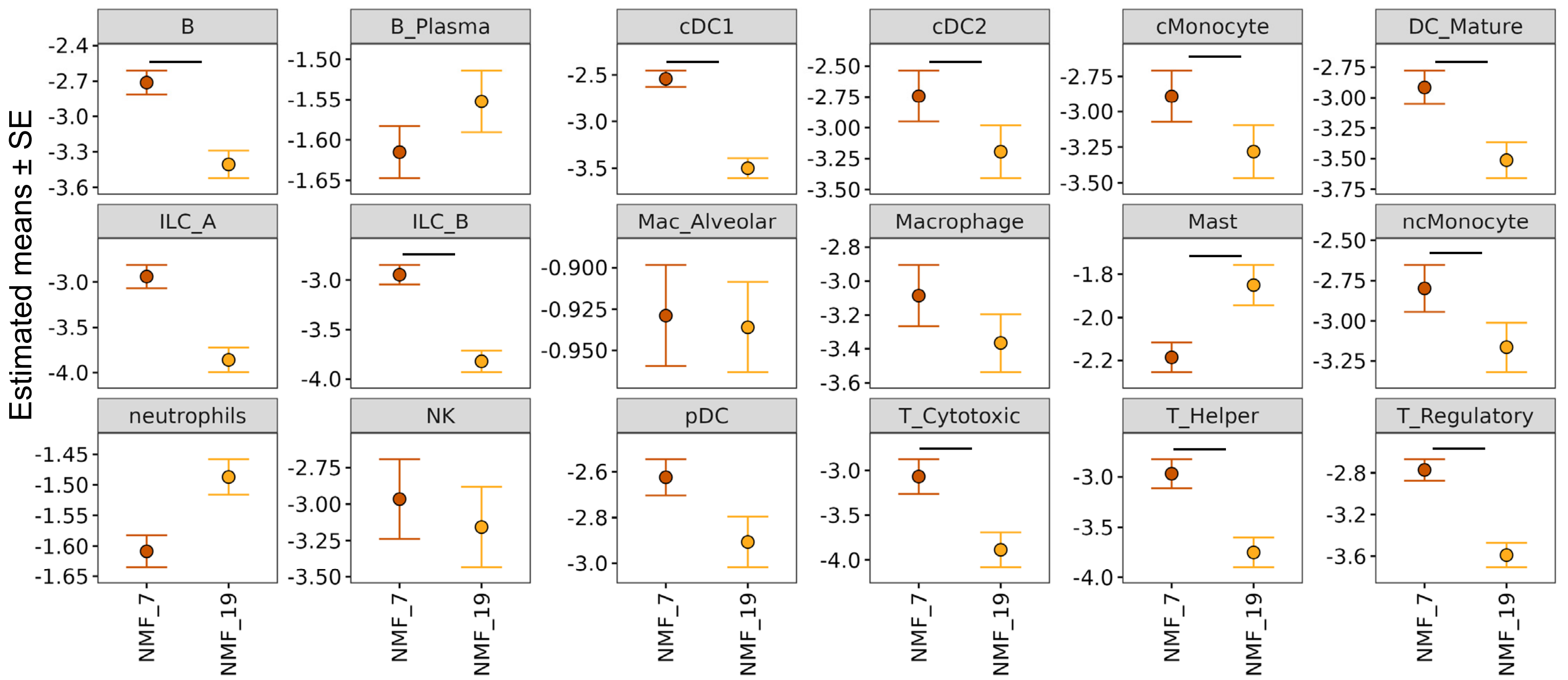

Figure S27: Cellular signatures across COPD-endobronchial and control-endobronchial niches

(A) Quantification of structural and (B) immune cells across COPD-endobronchial (NMF_7) and control-endobronchial niches (NMF_19). Significance was calculated with linear mixed model with individual sample as a random effect, niche as a fixed effect. P-values were adjusted with fdr; lines indicate $p_{adj} \leq 0.05$. Data was represented with estimated means ± standard errors.

Supplementary Figure S28

A Cell type associated with NMF_7 pathways

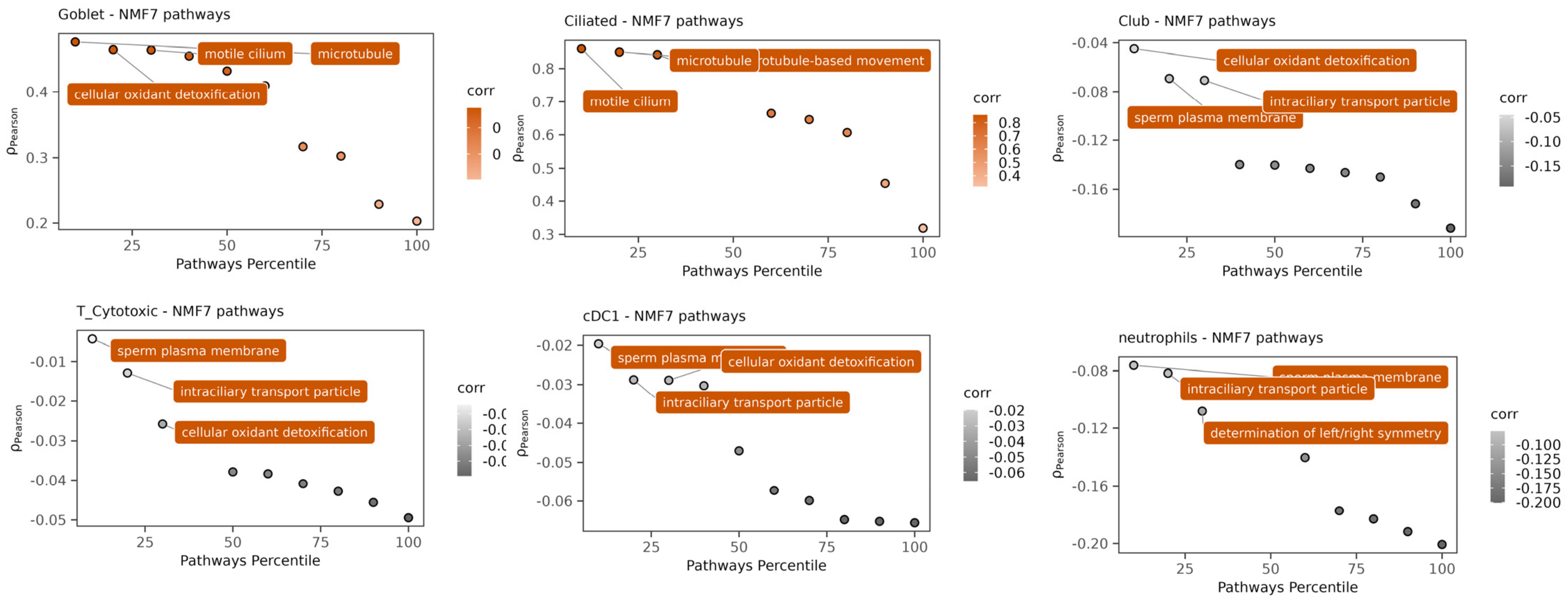

B Cell type associated with NMF_19 pathways

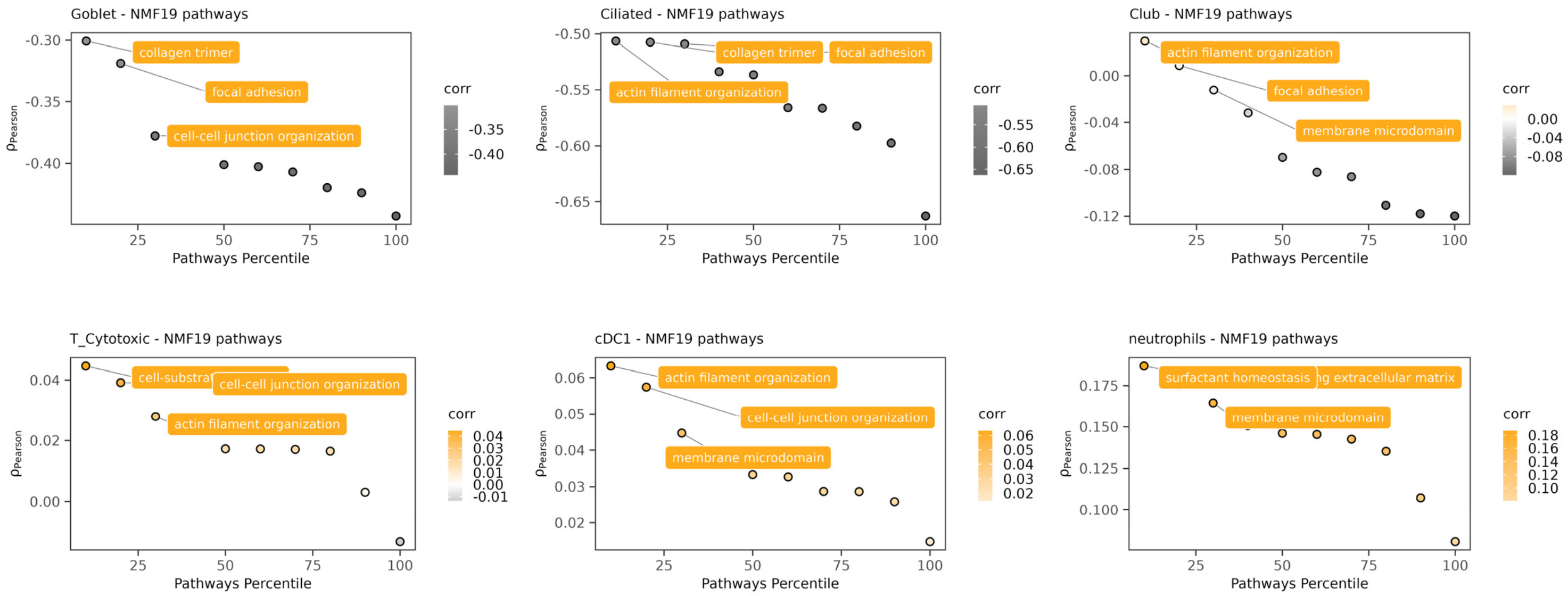

Figure S28: Certain cell types associate with signature pathways across COPD-endobronchial and control-endobronchial niches

(A) Correlation between deconvolution scores of selected cell types with enrichment scores of COPD-endobronchial (NMF_7) and (B) control-endobronchial (NMF_19) niches pathways. Pathway scores were computed using *Addmodulescore()* function from Seurat package based on genes identified within each corresponding pathway in Figure S19C. Correlation was calculated using pearson method on pooled airway niches spots.

Supplementary Figure S29

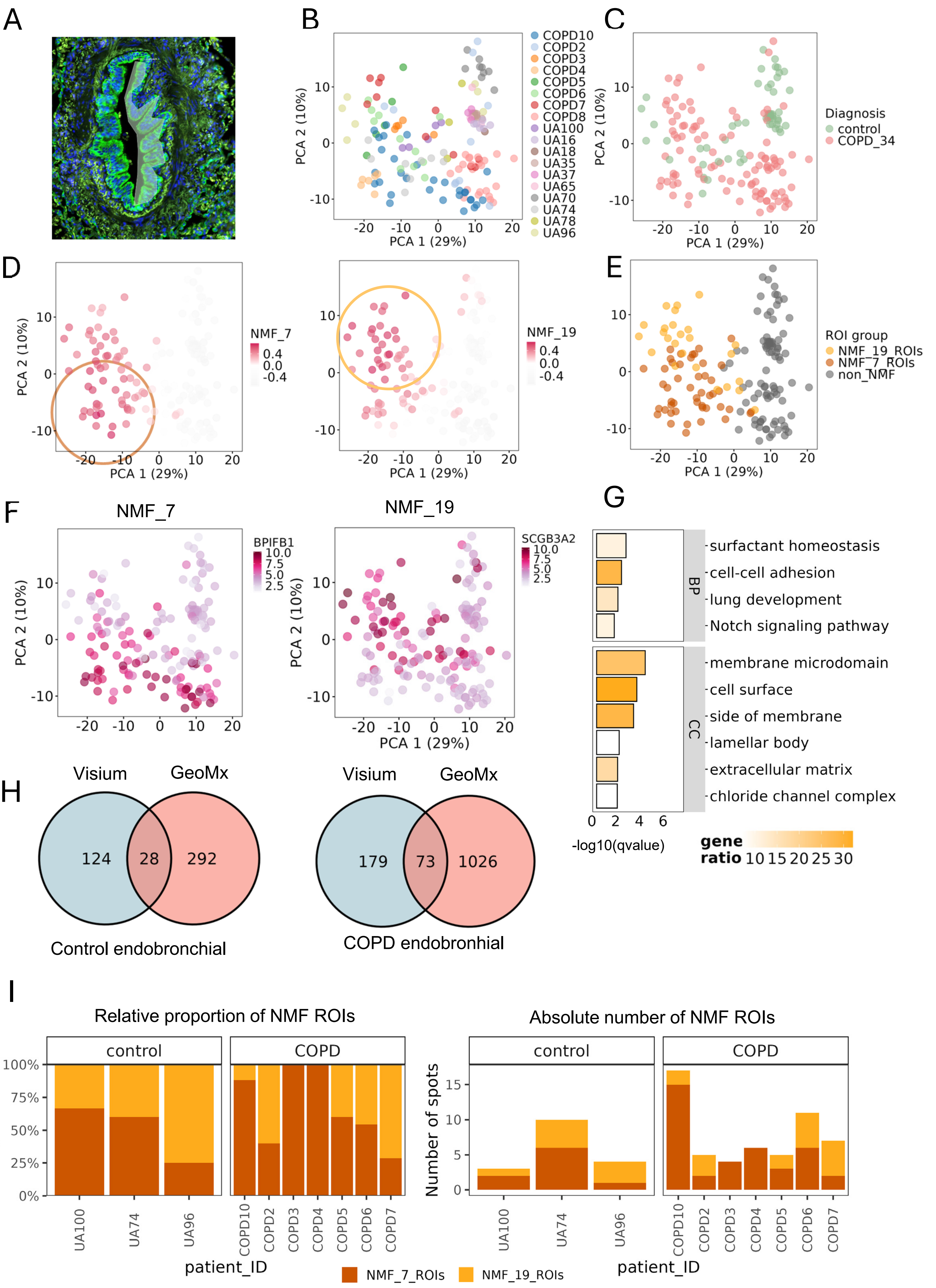

Figure S29: Identification of spatial niches associated with lung compartments

(A) Immunofluorescence staining showing a representative airway region of interest (ROI) in GeoMx Nanostring spatial transcriptomics dataset; blue: Syto13, green: PanCK. (A) Principal component analysis (PCA) was performed on all airway ROIs from controls and COPD lungs; each dot represents one airway ROI and was coloured by (B) individual patients and (C) disease. (D) PCA coloured by enrichment scores of COPD-endobronchial (NMF_7) and control-endobronchial (NMF_19) showing subsets of airway with high NMF_7 (dark orange circle) or NMF_19 scores (light orange circle). Enrichment score of COPD-endobronchial (NMF_7) and control-endobronchial (NMF_19) was calculated using GSVA R package using top 100 driving genes, excluding ribosome genes and non-detected genes. (E) Final classification of airway ROI groups. Airway ROIs with positive enrichment scores were classified as COPD-endobronchial ROIs (NMF_7_ROIs) or control-endobronchial ROIs (NMF_19_ROIs). (F) PCA exhibited subsets of airway ROIs with pronounced expression of selected top driving genes of COPD-endobronchial and control-endobronchial niches. (G) Bar chart of selected significantly enriched pathways (p_{adj} ≤0.05) of control-endobronchial ROIs. Pathway enrichment analysis was performed on downregulated genes (p_{adj} ≤0.05 & log2foldchange<0) in Figure 5F using clusterProfiler R package; BP: Gene Ontology Biological Process, CC: Gene Ontology Cellular Component. (H) Venn diagrams showing number of shared control-endobronchial (left) and COPD-endobronchial genes (right) between Visium spatial transcriptomics and GeoMx dataset. Differentially expressed genes (p_{adj} ≤0.05) between COPD-endobronchial and control-endobronchial niches in the Visium dataset was calculated by aggregating and grouping individual samples and niches, using DESeq2 R package and the significance was adjusted by Benjamini-Hochberg method. Differentially expressed genes between airway ROIs with high COPD-endobronchial signatures (NMF_7_ROIs) and airway ROIs with high control-endobronchial signatures (NMF_19_ROIs) in the GeoMx dataset was performed using linear mixed model with individual sample as random effect and airway groups as fixed effect and the significance was adjusted by fdr method. (I) Bar chart showing the relative proportion (left) and absolute number (right) of COPD-endobronchial and control-endobronchial ROIs across individual control and COPD samples relative to total number of airway ROIs in a given sample, color represents ROIs group.

Supplementary Figure S30

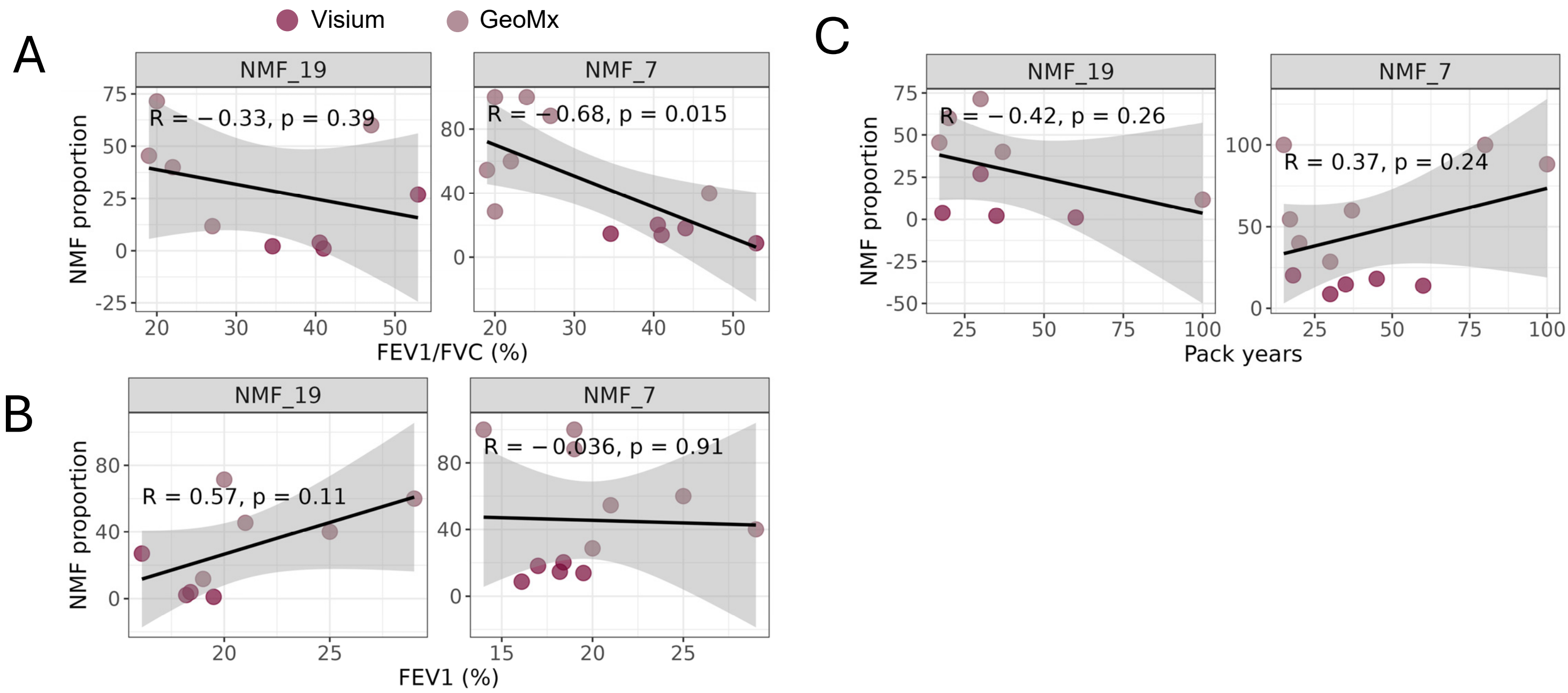

Figure S30: Clinical correlations of niche composition in Visium and GeoMx Nanostring spatial dataset

(A) correlation between relative airway niche proportion in the Visium and GeoMx spatial dataset and parameters of airflow limitation, FEV1/FVC ratio and (B) FEV1% as well as (C) smoking history. Correlation was calculated with pearson method.

Supplementary Figure S31

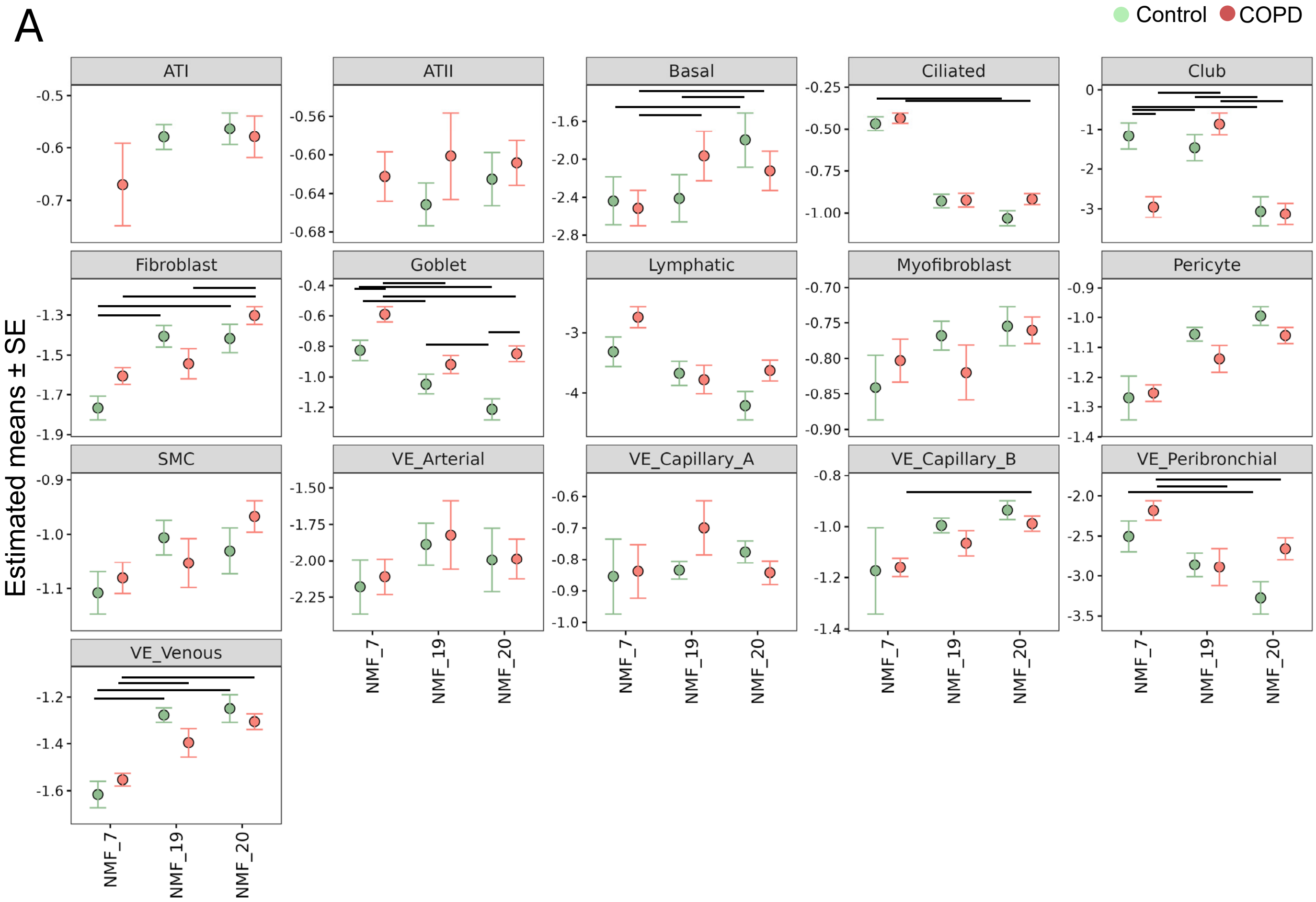

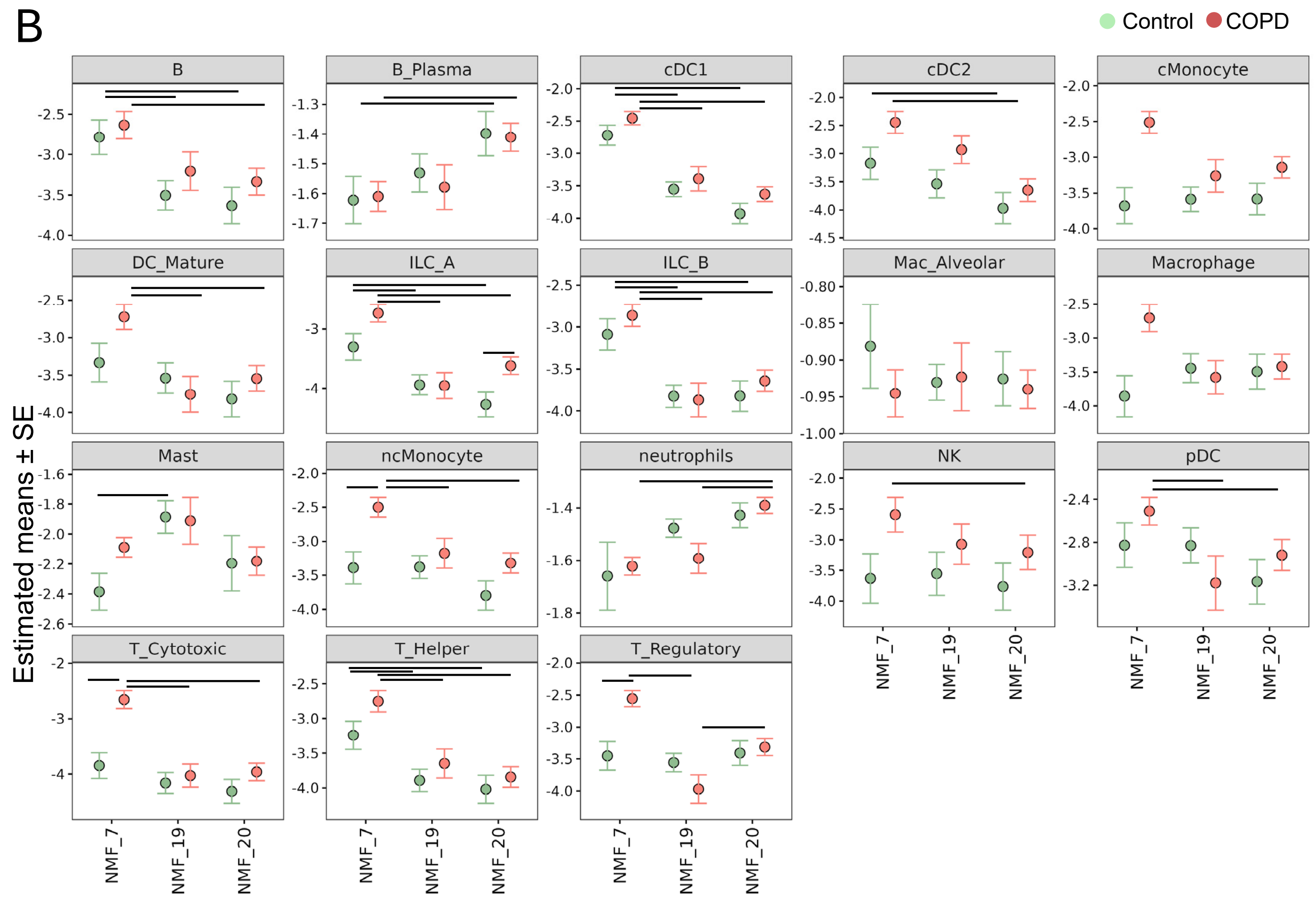

Figure S31: Disease associated deregulated cell populations across airway niches

(A) Quantification of selected structural and (B) immune cells in control and COPD across COPD-endobronchial (NMF_7), control-endobronchial (NMF_19), and COPD-peribronchial (NMF_14) niches. Significance was calculated with linear mixed model with individual sample as a random effect, diagnosis and niches as fixed effects. P-values were adjusted with fdr; lines indicate p_{adj} ≤0.1. Data was represented with estimated means ± standard errors.

Supplementary Figure S32

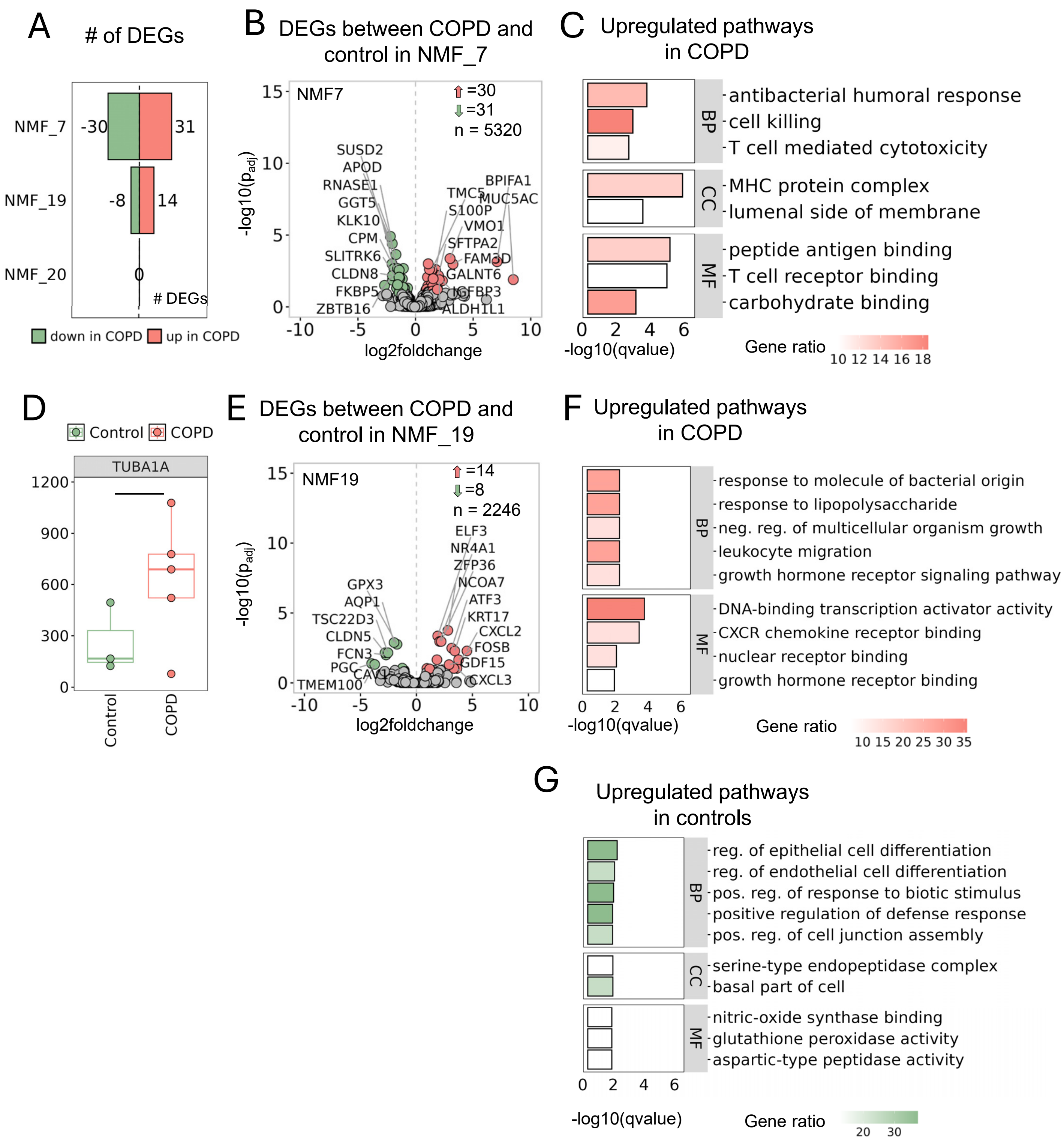

Figure S32: Disease associated transcriptional changes across airway niches

(A) Bar chart showing number of differentially expressed genes between control and COPD across COPD-endobronchial (NMF_7), control-endobronchial (NMF_19), and COPD-peribronchial (NMF_20) niches. Color represents the direction of regulation. Differentially expressed genes (p_{adj} ≤0.1) between COPD and controls, calculated independently in each niche. (B) Volcano plot showing differentially expressed genes between control and COPD in NMF_7, calculated using DESeq2 R package. Only significant genes (p_{adj} ≤0.1, adjusted by Benjamini–Hochberg method) were coloured and top 10 genes based on significance and log2foldchange level were labelled. (C) Bar plot of selected most significantly enriched pathways (qvalue ≤0.1) calculated from significantly upregulated genes (p_{adj} ≤0.1 & log2foldchange >0) in NMF_7. Colour represents the ratio between expressed genes and gene set size and x-axis corresponds to significance level. BP: Gene Ontology Biological Process, CC: Gene Ontology Cellular Component, MF: Gene Ontology Molecular Function. (D) TUBA1A gene expression between control and COPD within NMF_7, line indicate p_{adj} ≤0.05 (E) Volcano plot showing differentially expressed genes between control and COPD in NMF_19, calculated using DESeq2 R package. Only significant genes (p_{adj} ≤0.1, adjusted by Benjamini–Hochberg method) were colored and top 10 genes based on significance and log2foldchange level were labelled. (F) Bar plot of selected most significantly enriched pathways (qvalue ≤0.1) calculated from significantly upregulated genes (p_{adj} ≤0.1 & log2foldchange >0) and (G) significantly downregulated genes (p_{adj} ≤0.1 & log2foldchange >0) in NMF_19. Colour represents the ratio between expressed genes and gene set size and x-axis corresponds to significance level.

Supplementary Figure S33

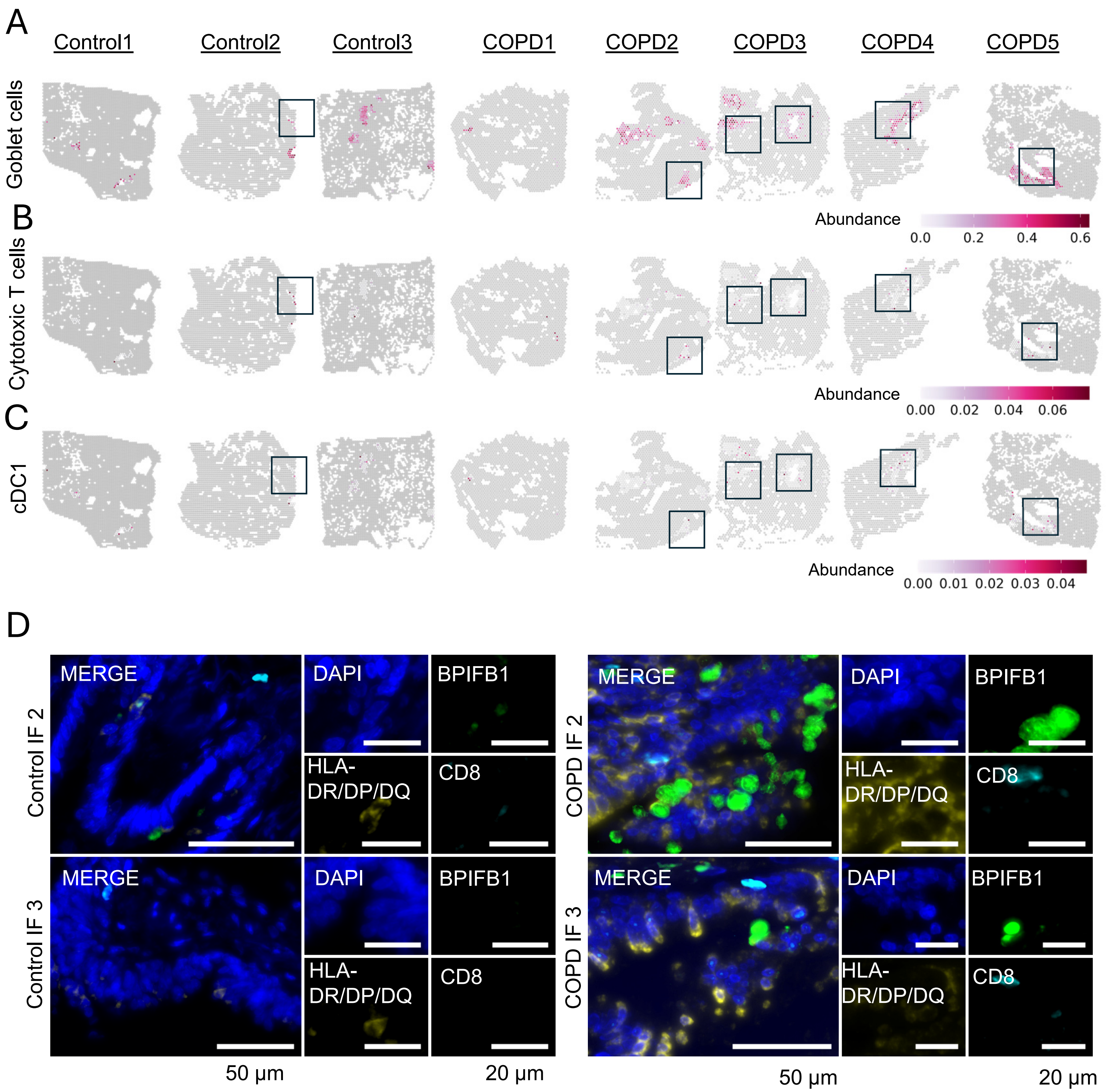

Figure S33: Spatial colocalization between goblet, cytotoxic T cells, and cDC1 within COPD-endobronchial niche.

(A) Spatial map showing spot proportion of deconvoluted goblet cells, (B) cytotoxic T cells, and (C) cDC1 within COPD-endobronchial niche (NMF_7) across control and COPD samples. Color represents deconvolution scores; cells colocalizations were indicated in squared boxes. (D) Multiplex immunofluorescence stainings on goblet cells ($BPIFB1^+$, green), cytotoxic T cells ($CD8^+$, turquoise), and antigen presenting cells ($HLA\text{-}DR/DP/DQ^+$, yellow) within airways of control and COPD. Scale bar is 50 µm.

Supplementary Figure S34

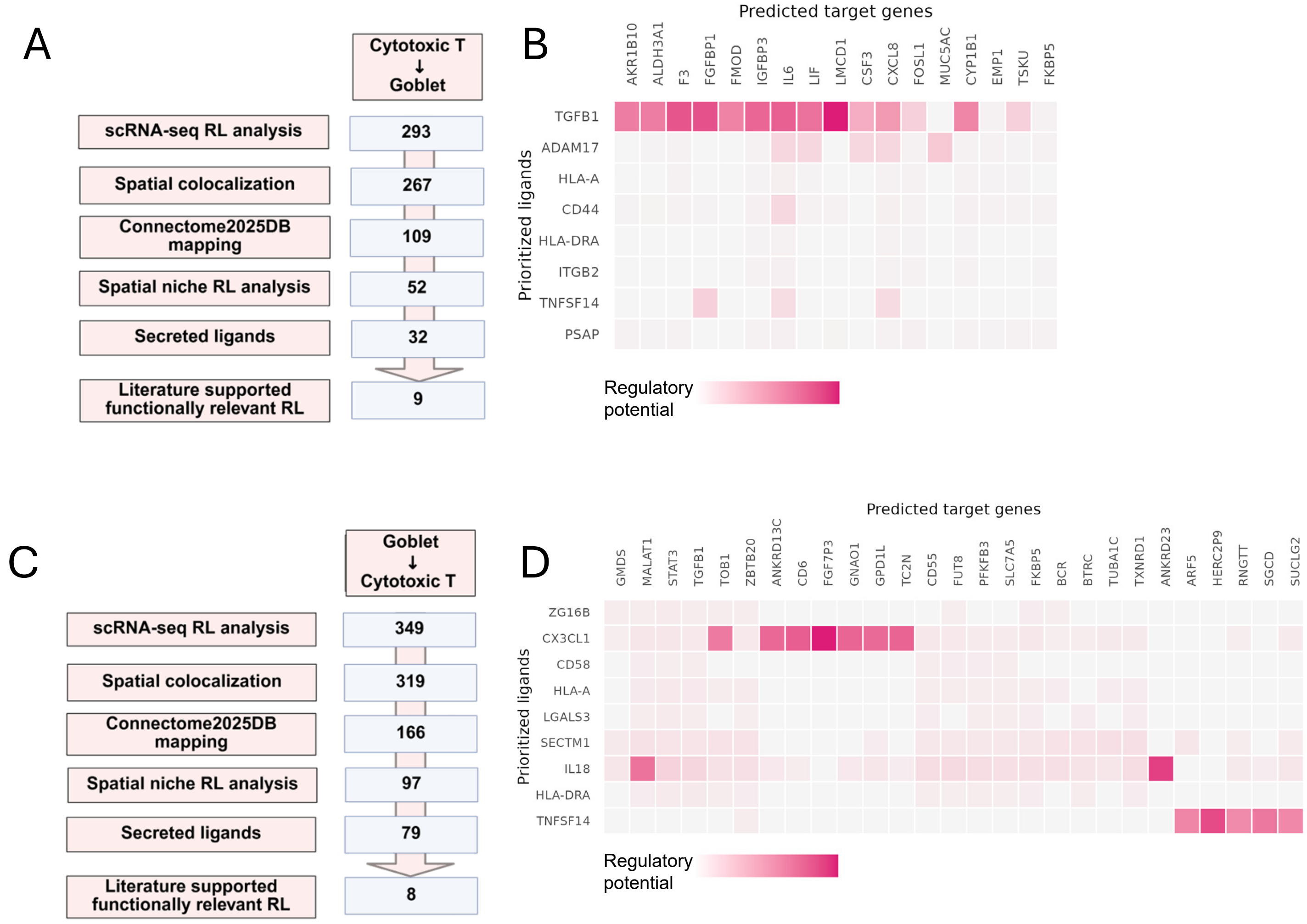

Figure S34: Predicted downstream target genes in receptor-expressing cells mediating goblet cell–cytotoxic T cell interactions signalling pathways.

(A) Workflow of identification target receptor-ligand interactions between cytotoxic T cells and goblet cells. (B) Heatmap showing the regulatory potential of selected ligands on downstream target genes as a results of ligand and receptor interactions in goblet cells. Target genes were calculated on integrated scRNA dataset (GSE136831, GSE171541) and predicted based on differentially expressed genes between COPD and controls in goblet cells and known protein-protein interaction database applied in nichenetr R package. (C) Workflow of identification target receptor-ligand interactions between goblet cells and cytotoxic T cells. (D) Heatmap showing the regulatory potential of selected ligands on downstream target genes as a results of ligand and receptor interactions in cytotoxic T cells. Target genes were calculated on integrated scRNA dataset (GSE136831, GSE171541) and predicted based on differentially expressed genes between COPD and controls in cytotoxic T cells and known protein-protein interaction database applied in nichenetr R package.

Supplementary Figure S35

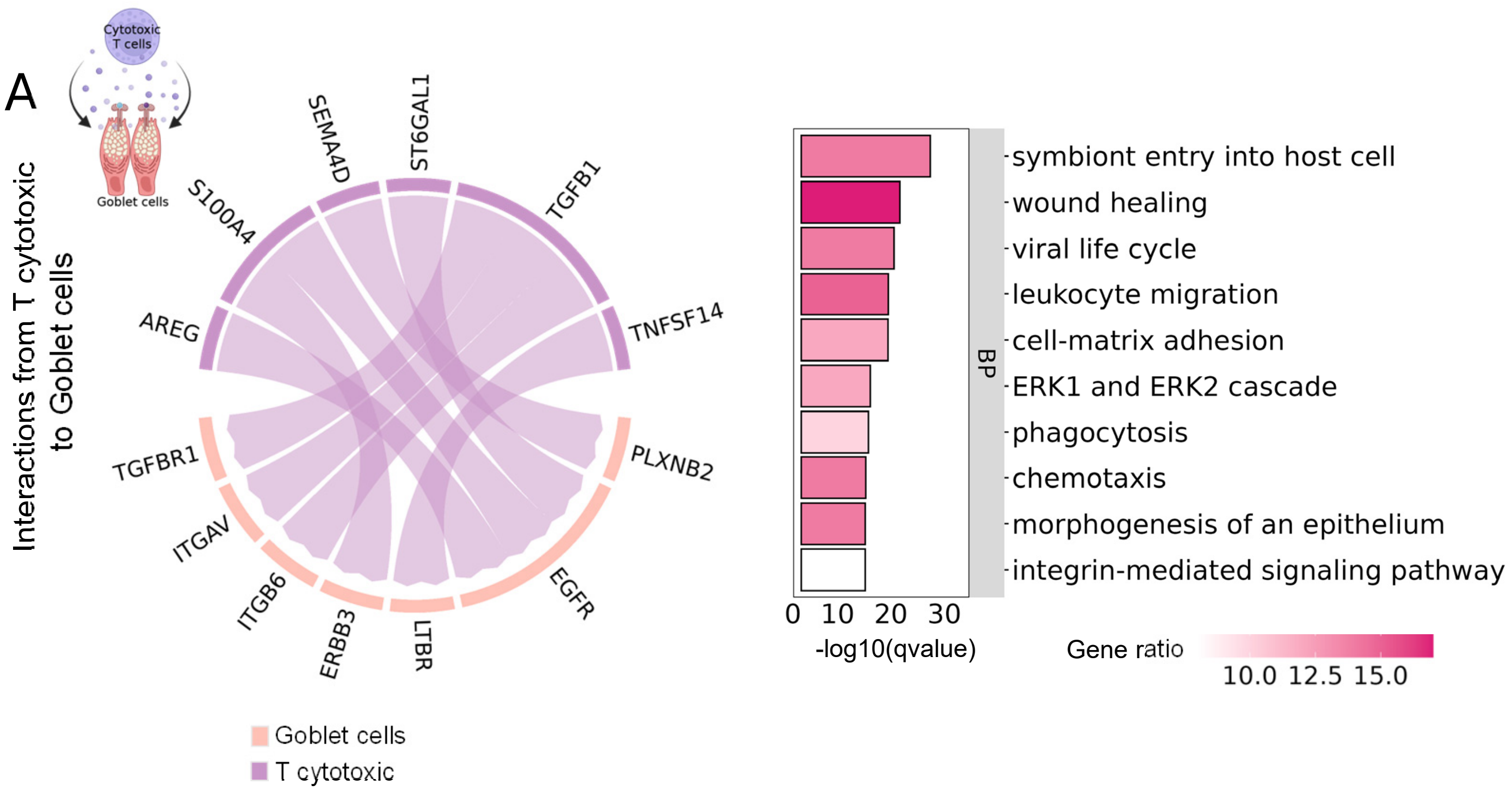

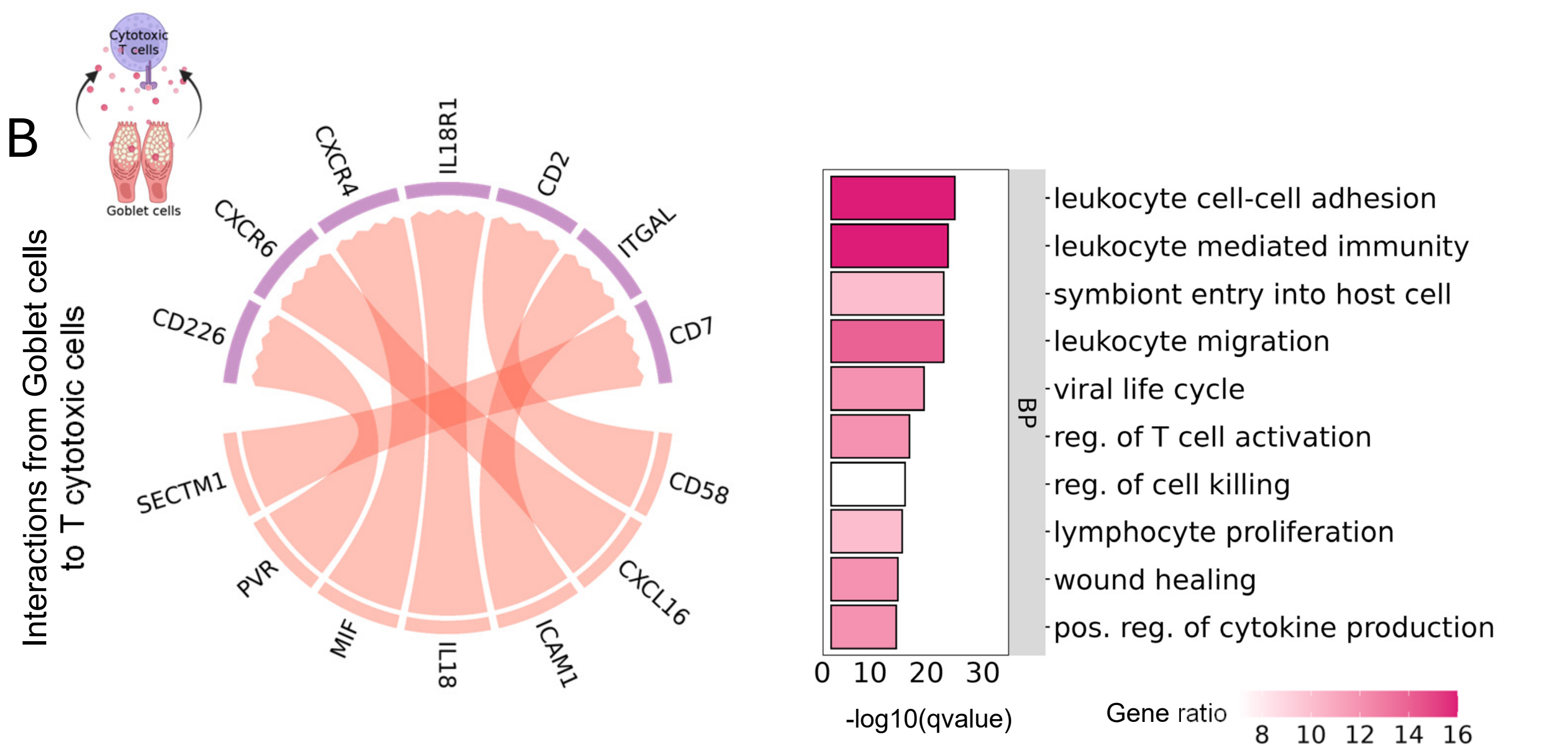

Figure S35: Cytotoxic T cells are predicted to interact with AT2 cells in the mild-moderate COPD lungs

(A) Ligand–receptor interactions inferred using the nichenetr R package on control and GOLD1-2 COPD scRNA-seq datasets (GSE173896). Circos plot showing prioritised ligands expressed by NK or cytotoxic T cells and their corresponding receptors on AT2 cells. Ligand–receptor pairs were filtered to retain those shown in Fig. 3C-D. Colours indicate cell types; directed edges represent signalling direction from sender to receiver. Bar plot of significantly enriched pathways (qvalue ≤ 0.05) derived from all identified receptors and target genes. Colour represents the ratio of expressed genes to gene set size, and the x-axis indicates significance. BP denotes Gene Ontology Biological Process. Violin plots show RNA expression of selected ligand or receptor genes across AT2 and cytotoxic T cells in COPD samples. Significance was assessed using Wilcoxon tests with FDR correction (* $p_{adj} \leq 0.05$, ** $p_{adj} \leq 0.01$, *** $p_{adj} \leq 0.001$, **** $p_{adj} \leq 0.0001$). (B) Circos plot of ligand–receptor interactions with AT2 cells as signal senders and NK or cytotoxic T cells as receivers, inferred using nichenetr and were filtered to retain those shown in Fig. 3E-F. Enriched pathways (qvalue ≤ 0.05) derived from all detected receptors and target genes, with corresponding violin plots showing expression of selected genes across AT2 and cytotoxic T cells in COPD.
